# TurboID Identification of Evolutionarily Divergent Components of the Nuclear Pore Complex in the Malaria Model Plasmodium berghei

**DOI:** 10.1128/mbio.01815-22

**Published:** 2022-08-30

**Authors:** Sushma V. Ambekar, Josh R. Beck, Gunnar R. Mair

**Affiliations:** a Iowa State University, Biomedical Sciences, Ames, Iowa, USA; NIAID/NIH

**Keywords:** *Plasmodium*, malaria, proximity labeling, BioID, TurboID, nuclear pore complex, nucleoporins, Nup

## Abstract

Twenty years since the publication of the Plasmodium falciparum and P. berghei genomes one-third of their protein-coding genes still lack functional annotation. In the absence of sequence and structural homology, protein-protein interactions can facilitate functional prediction of such orphan genes by mapping protein complexes in their natural cellular environment. The Plasmodium nuclear pore complex (NPC) is a case in point: it remains poorly defined; its constituents lack conservation with the 30+ proteins described in the NPC of many opisthokonts, a clade of eukaryotes that includes fungi and animals, but not Plasmodium. Here, we developed a labeling methodology based on TurboID fusion proteins, which allows visualization of the P. berghei NPC and facilitates the identification of its components. Following affinity purification and mass spectrometry, we identified 4 known nucleoporins (Nups) (138, 205, 221, and the bait 313), and verify interaction with the putative phenylalanine-glycine (FG) Nup637; we assigned 5 proteins lacking annotation (and therefore meaningful homology with proteins outside the genus) to the NPC, which is confirmed by green fluorescent protein (GFP) tagging. Based on gene deletion attempts, all new Nups — Nup176, 269, 335, 390, and 434 — are essential to parasite survival. They lack primary sequence homology with proteins outside the Plasmodium genus; albeit 2 incorporate short domains with structural homology to human Nup155 and yeast Nup157, and the condensin SMC (Structural Maintenance Of Chromosomes 4). The protocols developed here showcase the power of proximity labeling for elucidating protein complex composition and annotation of taxonomically restricted genes in Plasmodium. It opens the door to exploring the function of the Plasmodium NPC and understanding its evolutionary position.

## INTRODUCTION

Proteins that lack homologs in other species are referred to as orphan or taxonomically restricted proteins, coded by orphan genes or taxonomically restricted genes. They arise or diverge from pre-existing genes, or emerge *de novo* from non-genic sequences, and provide a mechanism for an organism to adapt, survive and thrive in its environment (1, 2). With few recognizable sequence features, proteins that build the nuclear pore complex (NPC) of Plasmodium parasites may represent such examples; the NPC may be composed of orphan proteins or have undergone substantial evolutionary divergence. Typically, the eukaryotic NPC is a megadalton assembly of multiple copies of 30+ nuclear pore proteins or Nups, which are embedded between the inner and outer nuclear membrane and line the nuclear pore. While the core scaffold and transmembrane Nups maintain the structure of the NPC, phenylalanine-glycine (FG)-Nups are natively disordered and characterized by multiple FG repeat domains that line the center of the pore and interact with cargo (3–6); a total of 552 proteins contribute to a single yeast NPC (7). The NPC carries out selective bi-directional nucleocytoplasmic transport of RNA and proteins while possessing numerous non-transport functions, including gene regulation. There appears to be considerable genetic diversity in the Plasmodium NPC make-up compared to other eukaryotes; this has so far prevented identification by amino acid sequence but also structural homology through bioinformatics even from relatively closely related species such as Toxoplasma gondii, a fellow member of the Apicomplexa (4, 8–13). To date, a mere 4 FG-nucleoporins (Nups) and Sec13 proteins are identified across the genus (12, 14–18), while P. falciparum Nup116 exhibits a low degree of conservation in rodent malaria parasites (19). The Plasmodium NPC remains, therefore, poorly defined. This parallels the absence of protein function annotation for many Plasmodium proteins. At present, 1,726 or 33% of the 5,302 protein-coding genes in the P. falciparum 3D7 genome are still annotated with an unknown function; in the rodent malaria model parasite, P. berghei, that number is 1,494 or 30% of 4945 protein-coding genes (PlasmoDB) (20).

An alternative to identifying the function of orphan proteins is to probe protein-protein interactions *in situ*. This has traditionally been achieved by identifying proteins co-immunoprecipitating with a known target, for example, mRNA storage bodies in P. berghei and P. yoelii gametocytes (21, 22). More recently, the emergence of proximity labeling (PL) approaches for discovering novel protein complex components or the constituents of entire subcellular compartments has greatly accelerated such efforts (23–26). Typically, these methodologies leverage the expression of a fusion protein that biochemically modifies proximal proteins, enabling their purification and identification by mass spectrometry. The original proximity-dependent biotin identification (BioID) system utilizes a biotin ligase (BirA) from Escherichia coli mutated (BirA*) to render it substrate-promiscuous (27). PL can also be achieved with modified soybean ascorbate peroxidase (APEX) and its derivatives, which require biotin phenol and hydrogen peroxide for biotinylation (28). Biotinylated proteins are subsequently isolated using a streptavidin affinity column and identified by mass spectrometry. Since its development, BioID has been employed in many organisms ranging from mammalian cells to plants and protozoans (26, 29). In Plasmodium, for example, BioID has revealed protein residents of secretory vesicles that are required for parasite egress from the infected erythrocyte and transmission (30); allowed the discovery of proteins in the parasitophorous vacuole that constitutes the host cell-parasite interface (31–34); generated a comprehensive proteome of the apicoplast (35); and defined the components of the inner membrane complex (36).

Here, we explored PL of the P. berghei nuclear pore complex. We produced fusion proteins of the smallest FG-repeats containing nuclear pore protein Nup138 with different biotin ligases — BioID/BirA*, BioID2, Linker-BioID2, TurboID, and mini-TurboID — to determine their suitability for the visualization of the NPC in the blood stages of P. berghei. We used streptavidin labeling as a rapid, 2-h read-out for the correct targeting and labeling efficiency. We demonstrate that *in vivo* biotinylation can be leveraged as an efficient visualization tool for studying the localization of proteins of interest as we observed consistent labeling at the nuclear periphery using TurboID at endogenous biotin levels present in mouse blood. We extended this study to all known FG-Nups (Nup205, Nup221, Nup313) and, for the first time, Nup637. We performed affinity purification of biotinylated proteins from a mutant expressing Nup313::TurboID, the only P. berghei FG-Nup so far confirmed independently in P. falciparum (15, 16). Importantly, mass spectrometric analysis of affinity-purified proteins from *nup313::turboID* resulted in the identification of 5 novel and unique Nups, all previously annotated as proteins of unknown function. Reciprocal PL with one of these candidate-Nups, Nup434, confirmed these interactions. Remarkably, all novel NPC components lack apparent homology to NPC components from other organisms, including related protozoans of the apicomplexan phylum, revealing an extraordinary evolutionary divergence of the NPC in this early-branching eukaryote.

## RESULTS

### TurboID biotinylation facilitates visualization of the P. berghei nuclear pore complex at physiological biotin concentrations in mouse blood.

Since the adaptation of BioID/BirA* to label and reveal protein-protein interactions, there have been continued efforts to improve this tool's efficiency. To reduce nonspecific interactions (for example, caused by erroneous targeting in the cell due to the large size of BioID) and improve the kinetics for biotinylation, a smaller second-generation system known as BioID2 was engineered from the bacterial Aquifex aeolicus biotin ligase (37). This protein is 27 kDa in size (as it lacks the DNA-binding domain), requires lower biotin concentrations, and performs well in mammalian cell culture (37) and Plasmodium systems (33) at 37°C. Since the inherent labeling radius of BioID2 is only about 10 nm, the inclusion of a 25-nm linker consisting of 13 repeats of GGGGS (G glycine, S serine) between bait protein and biotin ligase allows for a greater radius of labeling (37); this facilitates the detection of factors that may interact transiently or are beyond the range of the original system. Further evolution in BioID methodologies is represented by TurboID (38), a 35 kDa protein with 15 mutations compared to wild-type BirA; the protein produces efficient labeling of proteins in 10 min in the presence of 50 μm of biotin in mammalian cell culture. A smaller, 28 kDa miniTurboID version was achieved by deleting the N-terminal DNA binding domain.

**FIG 1:**
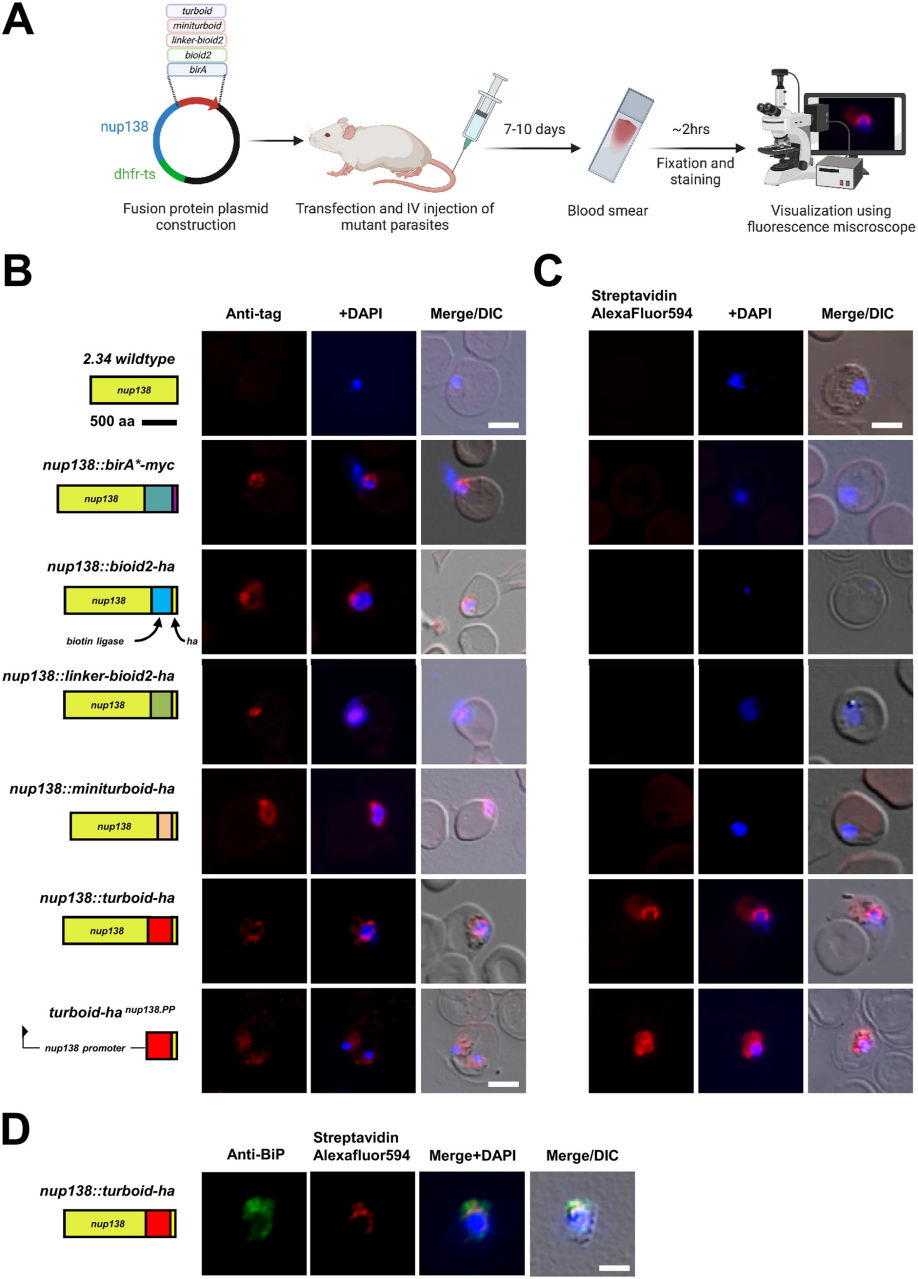
Nup138::TurboID facilitates visualization of the nuclear pore complex with biotin-binding Streptavidin Alexa Fluor 594 in blood-stage parasites. (A) Schematic diagram outlining the methodology used for the generation of transgenic *nup138* mutants and the visualization of various Nup138 fusion proteins. (B) Immunofluorescence assays with antibodies against 3X-HA and Myc epitope tags. DAPI is used to detect nuclear DNA (blue). Diagrams of Nup138 fusion proteins are drawn to scale; 0.5 cm = 500 amino acids. Scale bar = 5 μm. (C) Streptavidin Alexa Fluor 594 labeling of blood smear parasites. DAPI is used to detect nuclear DNA (blue). Scale bar = 5 μm. (D) Co-staining with Streptavidin Alexa Fluor 594 and anti-BiP (PBANKA_0818900, endoplasmic reticulum marker, ER) reveals distinct localization of ER and Nup138::turboID signals. DAPI is used to detect nuclear DNA (blue). All images are representative of three biological replicates with 10 randomly selected fields imaged. Scale bar = 5 μm.

To compare the labeling efficiencies of these various biotin ligases *in vivo*, we constructed mutant parasites expressing a range of carboxy-terminal fusion proteins with the endogenous P. berghei Nup138 (methodology outline) (Fig. 1A and Fig. S1A). Nup138 (the number defines the predicted molecular mass in kDa as in most Nups) was recently identified as an FG repeat nucleoporin; the protein is characterized by 14 dispersed FG repeats across its central region and is the smallest of the known FG-Nups conserved across the Plasmodium genus (12). Each fusion protein contains a triple hemagglutinin (HA) or Myc tag at the very C-terminus allowing protein localization in the absence of biotinylation to be verified by immunofluorescence. Protein expression in transgenic parasites is exclusively under the control of the endogenous promoter in the haploid blood stage of the parasite life cycle, which was achieved by introducing individual, linearized tagging plasmid into the parental locus. Wild-type parasites and a line expressing a non-fused, cytosolic TurboID under the control of the endogenous *nup138* promoter served as controls. Table S1 lists all cell lines established during this study.

10.1128/mbio.01815-22.1 Figure S1 Electrophoresis gel images of PCR genotyping of BioID and GFP-tagged parasite lines used in this study. (A) Schematic overview of the *in situ* tagging strategy for all parasite lines generated in this study and indication of mutant-specific PCRs. sm = selection marker. (B) PCR gel images for integration of biotin ligases fused to Nup138. Lanes 1 and 2 indicate integration at 5’ and 3’ sites. Lane 3 is a control for the selection marker gene and lane 4 is a genomic PCR control. All primers used are indicated below along with the expected amplicon size and ladder sizes are shown on the left in kb. (C) PCR gel images for integration of TurboID fused to FG-Nups. Lanes 1 and 2 indicate integration at 5’ and 3’ sites. Lane 3 is a plasmid control for the selection marker gene and lane 4 is a genomic control. All primers used are indicated below along with the expected amplicon size and ladder sizes are shown on the left in kb. (D) PCR genotyping for GFP and TurboID-tagged novel Nups. Lanes 1 and 2 indicate integration at 5’ and 3’ sites. Lane 3 is a plasmid control for the selection marker gene and lane 4 is a genomic control. All primers used are indicated below along with the expected amplicon size and ladder sizes are shown on the left in kb. Download FIG S1, PDF file, 0.4 MB. Copyright © 2022 Ambekar et al. 2022 Ambekar et al. https://creativecommons.org/licenses/by/4.0/ This content is distributed under the terms of the Creative Commons Attribution 4.0 International license.

10.1128/mbio.01815-22.7 TABLE S1 Mutant Plasmodium berghei genotypes of this study. Download Table S1, DOCX file, 0.01 MB. Copyright © 2022 Ambekar et al. 2022 Ambekar et al. https://creativecommons.org/licenses/by/4.0/ This content is distributed under the terms of the Creative Commons Attribution 4.0 International license.

Following confirmation of genomic plasmid integration (Fig. S1B), we first carried out immunofluorescence assays probing for the 3X-HA or Myc tag to verify the stable expression and correct localization of all Nup138 fusion proteins. All anti-tag assays detected the various fusion proteins at the nuclear periphery except for the wild-type control and the *turboid-ha ^nup138.PP^* mutant. The data provide evidence for the correct targeting of the tagged 138 nucleoporins (Fig. 1B).

Next, we evaluated the biotinylation signal using a streptavidin-conjugated Alexa Fluor probe. This method does not require primary and secondary antibodies but exploits the strong binding affinity between the probe and biotin or biotinylated proteins, hence reducing background signal levels and the incubation time; blood smear to the microscope is typically achieved within 2 h. In the intraerythrocytic stage, biotin is dispensable for parasite growth and is not conjugated to any parasite proteins (39). As expected, the parental wild-type P. berghei clone 2.34 revealed only a nuclear DAPI signal but no streptavidin labeling (Fig. 1C). Among all mutant lines, only the *nup138::turboid* parasite highlighted a distinct streptavidin signal close to the nuclear DNA consistent with earlier observations, when using green fluroescent protein (GFP), for Nup138::GFP (12). TurboID is thus the sole biotin ligase that labels the NPC at physiological biotin levels present in mouse serum, ranging between 6 to 15 ng/mL (40). Ninety four percent of examined *nup138::turboid-ha* parasites displayed perinuclear labeling indicating a high integration efficiency in this parasite population typical of genetically engineered P. berghei (41). In contrast, the negative control, TurboID expressed under the control of the *nup138* promoter, revealed no focal staining of the nuclear periphery, producing a diffuse cytoplasmic signal instead. Staining for the ER marker, BiP revealed the distinct and separate nature of the streptavidin NPC signal in *nup138::turboid-ha* parasites (Fig. 1D).

To evaluate the labeling performance of the various biotin ligase fusions that did not produce obvious labeling at physiological biotin levels, parasitized blood was incubated *ex vivo* overnight at 37°C with 200 μM biotin in standard RPMI culture medium. Under these conditions the parasite lines expressing Nup138 tagged with birA*, bioID2, linker-bioID2, and miniturboID all exhibited streptavidin labeling which could be observed by microscopy and Western blotting (Fig. S2), consistent with the relatively low expression level of most Plasmodium Nups and the superior kinetics of TurboID. In summary, while standard P. berghei *in vitro* culture conditions with biotin supplementation can be used to produce labeling with all biotin ligase variants, only the Nup138::turboID fusion exhibits sufficiently robust labeling kinetics under physiological mouse biotin serum levels to reliably visualize the P. berghei NPC.

10.1128/mbio.01815-22.2 FIG S2 Streptavidin labeling following *in vitro* culture with exogenous biotin. (A) Streptavidin blots of all Nup138::biotin ligase fusion proteins cultured overnight without or with 200 μM biotin. Bands indicated by an asterisks are host biotinylated proteins. The untagged, wild-type control on the left shows no biotinylation beyond biotinylated host proteins, whereas parasites lines with biotin ligase fusions to Nup138 show substantial biotinylation in the presence of exogenous biotin. (B) Streptavidin AlexaFluor 594 labeling of methanol:acetone fixed parasites following incubation overnight in regular media (RPMI+FBS) without or with 200 μM biotin. Parasites shown are in the late schizont stage or clusters of recently egressed merozoites. The prominent single focus of labeling is typical of Nup138 distribution at this stage. Images are representative of two independent experiments with 10 fields imaged. DAPI is used to detect nuclear DNA (blue). Scale bar = 5 μm. Download FIG S2, PDF file, 0.3 MB. Copyright © 2022 Ambekar et al. 2022 Ambekar et al. https://creativecommons.org/licenses/by/4.0/ This content is distributed under the terms of the Creative Commons Attribution 4.0 International license.

### TurboID tagging of FG-Nups 205, 221, 313, and authentication of Nup637 as a nuclear pore protein.

**FIG 2:**
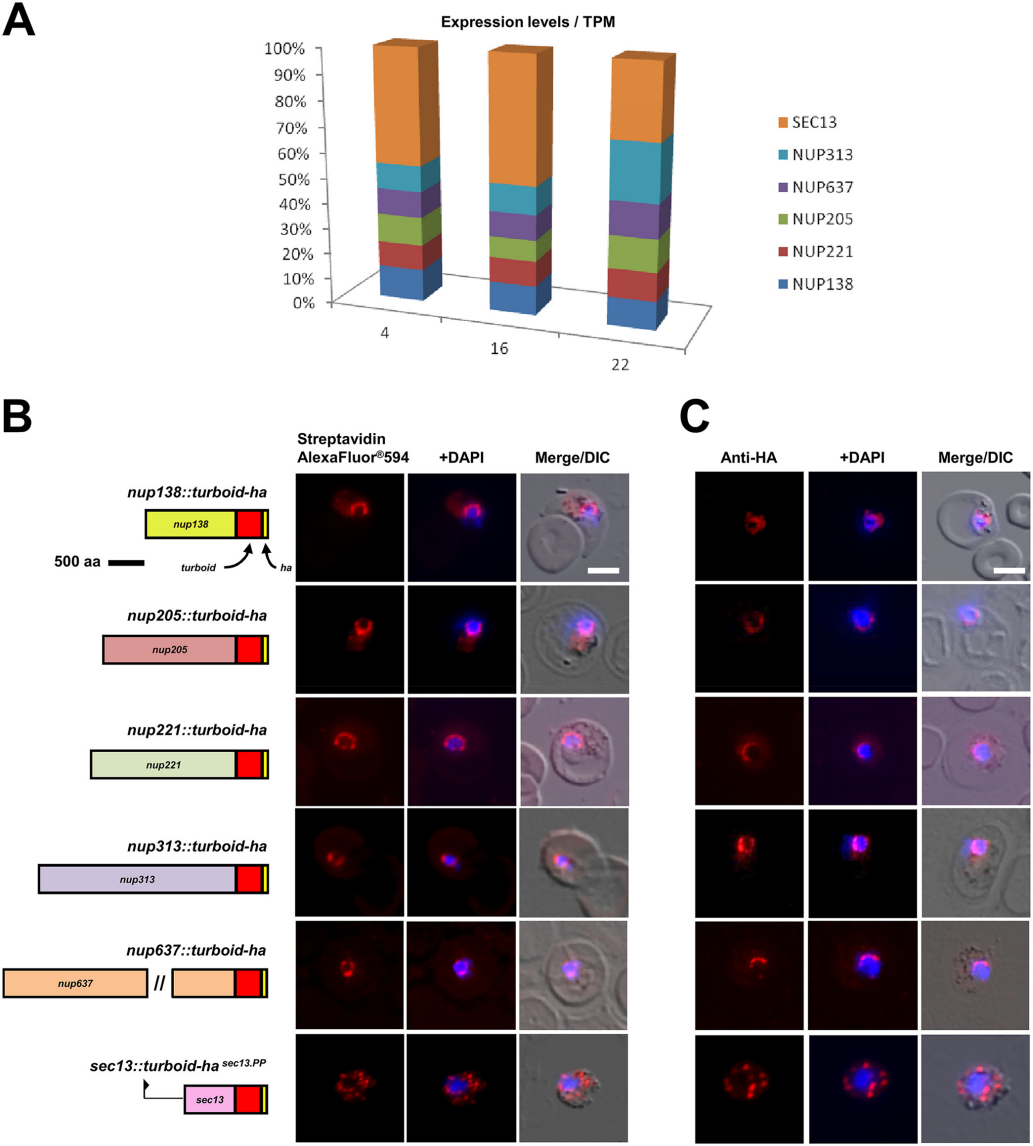
TurboID fusion proteins facilitate visualization of FG-Nups and localizes Nup637 to the nuclear periphery in blood-stage parasites. (A) Transcript abundance by RNAseq (76) plotted to show expression of Nups in 3 intra-erythrocytic stages: 4H ring, 16H trophozoite, 22H schizont. Expression levels are depicted as percentage of total Transcripts per Million (TPM) for all Nups shown in the bar chart. (B) Localization of TurboID-tagged nucleoporins detected with Alexa Fluor 594 conjugated streptavidin. Pictorial representations of fusion protein constructs used in the experiments are shown to the left and are drawn to scale; 0.5 cm = 500 amino acids. Scale bar = 5 μm. (C) Immunofluorescence assays using anti-HA antibodies confirm protein localization at the nuclear periphery. Scale bar = 5 μm.

Following the successful establishment of TurboID as an efficient *in vivo* proximity labeling tool in P. berghei, we extended our study to the 4 remaining Plasmodium FG-Nups 205, 221, 313, and 637. Apart from Nup637, each of the FG-Nups has previously been localized by fluorescent protein tagging to the nuclear periphery in P. berghei (12). Analogous to TurboID-tagging of Nup138, we transfected linearized plasmid constructs promoting integration into the P. berghei genome, thus maintaining expression of each fusion under the control of the endogenous promoter (Fig. S1C). With similar, coordinated transcription levels of all FG-Nups across the P. berghei intraerythrocytic developmental cycle (Fig. 2A), Nup205::turboid-HA, Nup221::turboid-HA, Nup313::turboid-HA, and Nup637::turboid-HA could be mapped to the nuclear periphery by anti-HA immunofluorescence assay as well as streptavidin staining (Fig. 2B and C). For the first time, we could confirm the localization of Nup637 at the nuclear periphery.

Three attempts to introduce TurboID-tagged Sec13, the most conserved of all known nuclear pore proteins across the Plasmodium genus, failed. On the other hand, expressing Sec13::turboID under the control of its endogenous promoter from an episomal plasmid revealed streptavidin labeling reflecting its dual function as Nup and COPII vesicle component with perinuclear staining as well as signal similar to that of its COPII partner Sec31 (Fig. 2B and C) (12, 14).

A challenge to studying the Plasmodium NPC is the lack of experimental evidence to label this structure in the parasite by other means. Attempts to label a broad range of Plasmodium FG nucleoporins with the widely used monoclonal antibody 414, which recognizes FXFG repeats found for example in human Nup62, Nup152, and Nup90 (42, 43), failed (Fig. S3A). Perhaps not surprisingly, out of 95 FG repeats known from all 5 P. berghei FG-Nups, only 4 adhere to this otherwise broadly conserved feature (12). Fluorescently conjugated Wheat Germ Agglutinin (WGA), a carbohydrate-binding lectin that recognizes O-linked β-*N*-acetylglucosamine (O-GlcNAc), labels the NPC in other systems as this posttranslational modification is frequent in the unstructured regions of FG-Nups (44). However, WGA binds extensively to the RBC surface, largely obscuring the intracellular parasite. While this problem can be alleviated by examining extracellular merozoites (Fig. S3B), WGA labeling cannot be used to study NPCs in other blood stages due to their residence within the RBC. Aleuria aurantia lectin (AAL) is specific for fucosylated glycans that have been detected proximal to the *Toxoplasma* NPC (45), but did not label any defined feature near the P. berghei nucleus (Fig. S3C). As these common approaches to NPC labeling used in other eukaryote systems cannot be used in Plasmodium spp., our Nup-TurboID/Streptavidin strategy provides an efficient and sensitive alternative for visualizing the Plasmodium NPC.

10.1128/mbio.01815-22.3 FIG S3 mAB414 and lectin staining of P. berghei parasites. (A) Immunofluorescence assay with mab414 fails to detect FG-Nups. (B) WGA conjugated to AlexaFluor 488 (green) was used at 1:500 dilution for staining infected erythrocytes and free merozoites following MACS purification. DAPI (blue) was used to stain the nucleus. Extensive staining of glycosylated proteins of the host RBC obscured the parasites. In extracellular merozoites unobscured by the RBC staining, a punctate signal is seen which conforms to the NPC localization signal at this stage. Scale = 2 μm. (C) Parasites stained with AAL lectin conjugated to AlexaFluor 488 did not display a nuclear periphery labeling. Download FIG S3, PDF file, 0.1 MB. Copyright © 2022 Ambekar et al. 2022 Ambekar et al. https://creativecommons.org/licenses/by/4.0/ This content is distributed under the terms of the Creative Commons Attribution 4.0 International license.

### Mass-spectrometric analysis of *nup313::turboid* proximity-labeled parasite lysates identifies known and novel Plasmodium berghei NPC components.

**FIG 3:**
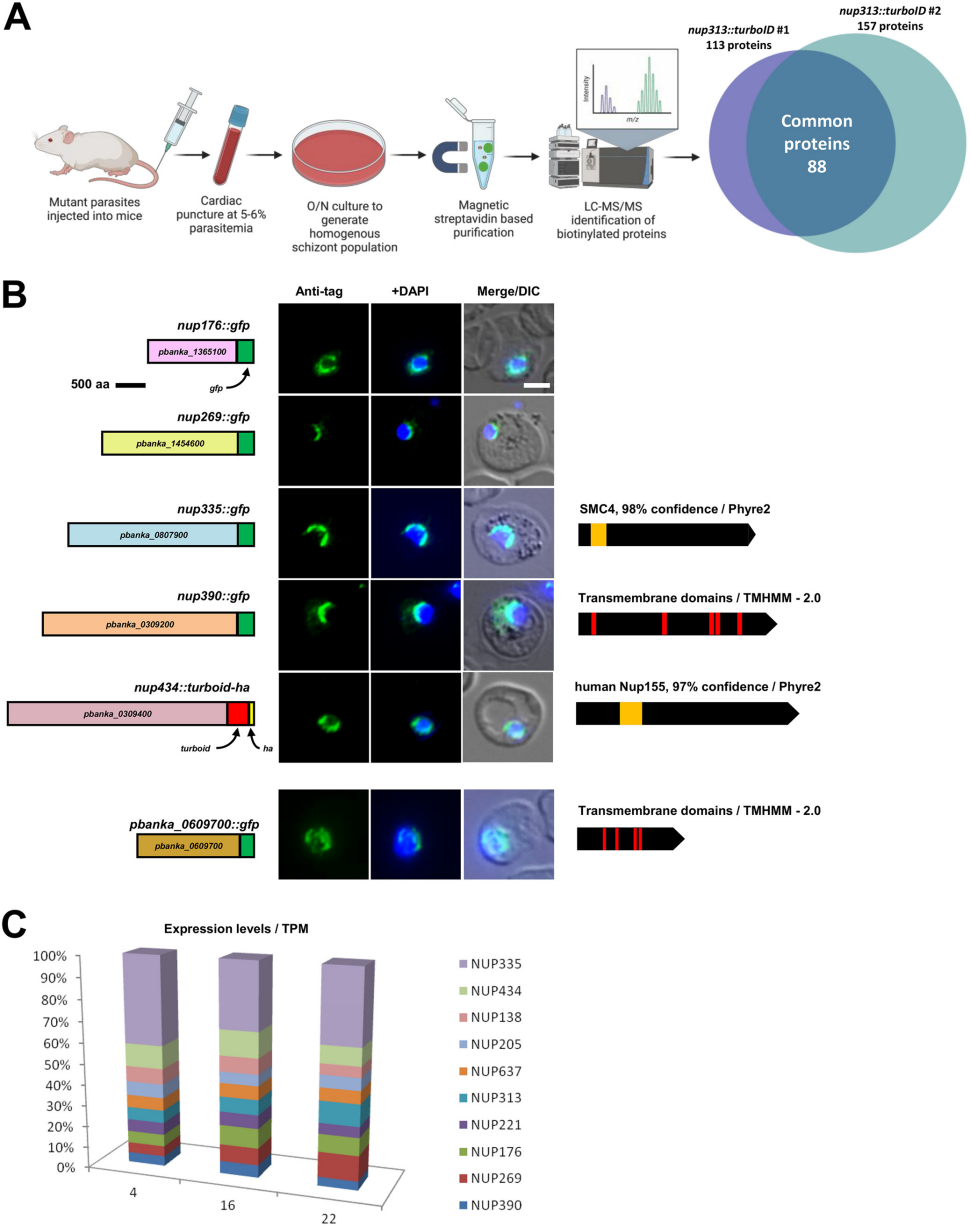
313::turboID proximity labeling and mass spectrometry identify novel Plasmodium nucleoporins. (A) Flowchart showing the methodology used for carrying out PL and mass spectrometry with TurboID-fused Nup313. (B) Localization of tagged nucleoporins detected with anti-GFP and anti-HA immunofluorescence. DAPI (blue) shows position of the nucleus. Schematic representations of constructs used for the experiment are show on the left where 0.5 cm = 500 amino acids. Phyre2 models are shown to the right of the image panel. Scale = 5 μm. (C) Transcript abundance by RNAseq (76) plotted to show expression of novel and FG-Nups in 3 intra-erythrocytic stages: 4H ring, 16H trophozoite, 22H schizont. Expression levels are depicted as percentage of total Transcripts per Million (TPM) for all genes shown in the bar chart.

Next, we employed the *nup313::turboid* line for the identification of novel NPC components. Apart from Sec13 (12, 14), only Nup313 has been confirmed as an NPC protein in another Plasmodium spp. (15, 16), thus presenting an attractive candidate for mass spectrometry experiments. We grew *nup313::turboid* parasites to a parasitemia of 5–6% and produced a homogenous schizont-rich population through overnight culture. *In vitro* P. berghei undergoes IDC development but fails to egress spontaneously from the iRBC (46, 47). To identify proteins by proximity labeling, we prepared *nup313::turboid* lysates followed by affinity purification of biotinylated proteins on magnetic streptavidin beads and protein identification by mass spectrometry (Fig. 3A). As TurboID is already visibly active in an *in vivo* setting (Fig. 2), we did not provide additional biotin but rather relied on the biotin levels present in mouse serum (6 to 15 ng/mL) and RPMI (200 ng/mL). We analyzed 2 independent biological *nup313::turboID-ha* samples alongside a wild-type control. We identified 113 and 157 proteins in samples 1 and 2, respectively, that were not detected in the wild-type control. Furthermore, 88 proteins were common to both biological replicates (Fig. 3A).

This data set included the bait protein Nup313 as well as all 5 FG-Nups 138, 205, 221, 313, and 637 but not Sec13 (Table S2). The bait, Nup313, and another FG-Nup, Nup637 were present in the top 50^th^ percentile of all proteins identified with normalized spectral abundance factor (NSAF) values of 113 and 155, respectively. Of the 88 detected proteins, 41 contained a predicted nuclear localization signal (48); this included all known FG-Nups apart from Nup138 (Table S2). Of the 88 proteins 38 had been detected in the nuclear core proteome of P. falciparum, including all FG-Nups (49) (Fig. S4).

10.1128/mbio.01815-22.4 FIG S4 Gene ontology analysis of proteins identified by proximity labeling by Nup313::TurboID. (A) Proteins common to both replicates and not present in wild-type controls were analyzed using PANTHER classification tool and grouped by protein class and biological function. (B) 38 out of 88 proteins were found in the Plasmodium core nuclear proteome published in a previous study (49). Overlapping proteins include all FG-Nups as well as select novel Nups (Nup335 and Nup176) identified in the present study. Download FIG S4, PDF file, 0.2 MB. Copyright © 2022 Ambekar et al. 2022 Ambekar et al. https://creativecommons.org/licenses/by/4.0/ This content is distributed under the terms of the Creative Commons Attribution 4.0 International license.

10.1128/mbio.01815-22.8 TABLE S2 Proteins identified in Nup313::turboID mass spectrometry experiment. Download Table S2, XLSX file, 0.02 MB. Copyright © 2022 Ambekar et al. 2022 Ambekar et al. https://creativecommons.org/licenses/by/4.0/ This content is distributed under the terms of the Creative Commons Attribution 4.0 International license.

A Protein ANalysis THrough Evolutionary Relationships (PANTHER) gene-ontology (GO) analysis classified 39 of the 88 hits into families of chaperones, cytoskeletal proteins, transporters, translational proteins, and those involved in biochemical processes, while most proteins (56%) lacked a GO annotation, including the known FG-Nups, owing to the large number of unannotated Plasmodium proteins in that database (Fig. S4) (50). Consistent with selective PL at the NPC, our mass spectrometry data set contained all 5 annotated NUPs, proteins known to traverse the NPC or those with a role in nuclear biology. This included for example 15 small and large ribosomal proteins (24%), as well as factors associated with ribosome biogenesis and transport. Such Nup313-ribosomal protein interactions can occur during the trafficking from the cytoplasm to the nucleolus for ribosome assembly and the subsequent export of the mature translation machinery (51, 52). Interactions with Nup313::turboID may also occur during bait translation, folding, and trafficking prior to insertion into a stable NPC; such factors are represented by chaperones, proteins localized at the ER, and translation factors. Furthermore, we detected the DNA/RNA-binding protein Alba 3, a highly repetitive RNA binding protein with a single RNA recognition motif (RRM), the RNA helicase DBP1, and SENP1 which has documented roles in mRNA surveillance and DNA repair while associating with the NPC (53) and functions in the maturation of small Ubiquitin-like modifier (SUMO), and removal of SUMO from modified proteins in concert with the proteasome during DNA synthesis (53, 54). We also identified four cytoskeletal proteins: actin 1, α-tubulin 1 and 2, and β-tubulin. Recent work has demonstrated the localization of microtubules using the tubulin marker, SPY555-Tubulin in the P. falciparum nucleus, close to the NPC, by using Nup313 to define the nuclear boundary during cell division and closed mitosis (16).

With an aim to identifying novel Nups, we focused on 8 proteins within our data set that were conserved among Plasmodium spp. but lacked functional annotation: PBANKA_0309200, PBANKA_0309400, PBANKA_0609700, PBANKA_0807900, PBANKA_1211700, PBANKA_1365100, PBANKA_1404700, and PBANKA_1454600. Of those, PBANKA_1211700 and PBANKA_1404700 contained predicted signal peptides and were not pursued any further. Among the remaining candidates, PBANKA_0309200, PBANKA_0309400, and PBANKA_1365100 were similar in spectral abundance (NSAF) to the bait, Nup313 (Table S2). As a first step toward their characterization, we performed C-terminal GFP-tagging with genomic integration (Fig. S1D) to establish their subcellular localization; turboID-HA tagging was used for PBANKA_0309400 as GFP-tagging failed repeatedly (*n* = 5). Five of the 6 candidate proteins localized to the nuclear periphery typical for FG-repeat nucleoporins in P. berghei (Fig. 3B). In contrast, PBANKA_0609700 presented a diffuse cytoplasmic localization despite the presence of four predicted transmembrane domains (Fig. 3B). Apart from the localization to the nuclear periphery, the transcriptome profiles of the new nucleoporins were similar to those of the 5 annotated FG-Nups with the exception of Nup335 (Fig. 3C).

We forthwith refer to these newly identified P. berghei nucleoporins according to their predicted molecular weights in kDa as: Nup176, Nup269, Nup335, Nup390, and Nup434 (Table 1).

**TABLE 1:** Known and newly identified P. berghei nucleoporins

Gene ID	Genbank acc.	Annotation	PlasmoGEM^*b*^	NLS	Plasmobase	Domain architecture
PBANKA_0417900	XP_034420236	Nup138	Essential	0	No hits	2 CC^*c*^, FG^*d*^ repeats
PBANKA_1140100	XP_034422607	Nup205	Essential	1	No hits	2 CC, FG repeats
PBANKA_0416300	XP_034420220	Nup221	Essential	1	Nup100	FG repeats, 2 TM^*e*^
PBANKA_1310200	XP_034423255	Nup313	Essential	4	No hits	1 CC, FG repeats
PBANKA_0107600	XP_034419676	Nup637	Essential	3	LAMP	FG repeats
PBANKA_1445400	XP_034424278	Sec13	Essential	0	IKI3	WD domain
PBANKA_0309200	XP_034419976	**Nup390** ^*a*^	Essential	1	No hits	3 CC, 5 TM
PBANKA_0309400	XP_034419977	**Nup434**	Essential	4	Nucleoporin_N	1 CC, beta-propeller
PBANKA_0807900	XP_034421049	**Nup335**	No Data	2	No hits	12 CC
PBANKA_1365100	XP_034423807	**Nup176**	No Data	3	No hits	1 CC
PBANKA_1454600	XP_034424373	**Nup269**	No Data	9	No hits	3 CC

a All newly identified Nups (bold font) were annotated as ‘conserved Plasmodium proteins, unknown function’ in PlasmoDB.

b The PlasmoGEM column indicates the essential/redundant nature of each gene in a global gene deletion screen; NLStradamus was used to scan each protein for the presence of nuclear localization signals; TMHMM to predict the number of transmembrane domains; SMART to identify coiled-coil domains and Plasmobase for identifying similar proteins based on protein domain architectures. To build models based on protein domain homology, Phyre2 was employed, and domain architecture hits with significant homology and confidence are listed. As a comparison, known FG-Nups are shown in the TABLE to demonstrate the poor detection of Plasmodium nucleoporins through homology modeling.

c CC, coiled coil domains.

d FG, phenylalanine-glycine repeats.

e TM, transmembrane domains.

Together with Sec13 (12, 14) and 4 previously identified FG-Nups (12) we have identified a repertoire of 11 NPC components in P. berghei. A *post hoc* enrichment analysis of the *nup313::turboid-ha* proximity labeling experiment shows a 53-fold enrichment of nucleoporins in our proteomic data (hypergeometric *P*-value = 1.24e-17).

All 5 new Nups are conserved in the human malaria parasite P. falciparum 3D7 strain (Fig. S5); they are encoded by syntenic genes but typically larger than their P. berghei orthologs. All lack detectable primary sequence homology outside the Plasmodium genus. Therefore, we probed all newly identified Nups with 6 different bioinformatic protein tools (Table S4). For Nup434, Phyre2 [55], a bioinformatics tool to predict and analyze protein structure generated a structural model with 97.1% confidence and 31% coverage based on the human nuclear pore subunit, Nup155 (Fig. 3B). The model covers only 365 amino acids of this 3,656 amino acid long protein, spanning amino acids 747 to 1,112. Nup155 is a non-FG, structural Nup and part of the inner ring of the human NPC, which includes Nup205, Nup188, Nup93, and Nup35. Nup155 plays a key role in the transcriptional response to DNA damage, specifically activating the cyclin-dependent kinase inhibitor p21 in the tumor suppressor p53 pathway (55); this pathway is not conserved in Plasmodium. In addition to human Nup155, Phyre2 returned 2 additional hits (>95% confidence) to inner-ring Nups from yeast (Nup157 with amino acid similarity from position 877 to 1,110) and Chaetomium thermophilum (Nup170 with amino acid similarity from position 747 to 1,087) (56). In the thermophilic eukaryote, C. thermophilum, Nup170 is required for NPC assembly and organization and, together with Nup192, Nic96, and Nup53, forms the inner pore ring. Furthermore, yeast Nup170 is physically linked to the TM domain-containing pore membrane protein, Pom152, thus stressing its role in the anchorage of the NPC. The protein domain architecture comparison instrument Plasmobase (57) revealed similarity of Nup434 to the N-terminus of Nup133, a human Y-complex Nup. The N-terminus of this Nup forms a seven-bladed beta-propeller structure which is also characteristic of Nup155 and Nup170 in this family and which were recognized in our analysis with Phyre2. Finally, HHpred (Homology detection & structure prediction by HMM-HMM comparison) (58, 59) recognized homology with Nup170 from Chaetomium. Collectively, these analyses suggest that Nup434 is a component of the inner ring of the Plasmodium NPC with remarkable evolutionary divergence from significantly smaller Nups identified in other eukaryotes.

10.1128/mbio.01815-22.5 FIG S5 ClustalW alignment of P. berghei candidate-Nups and their P. falciparum syntenic orthologs. Boxshading provided by http://www.ch.embnet.org/software/BOX_form.html. (A) ClustalW alignment of P. berghei NUP176 and P. falciparum ortholog. (B) ClustalW alignment of P. berghei NUP269 and P. falciparum ortholog. (C) ClustalW alignment of P. berghei NUP335 and P. falciparum ortholog. (D) ClustalW alignment of P. berghei NUP390 and P. falciparum ortholog. **** indicate 5 SMART-predicted transmembrane domains. (E) ClustalW alignment of P. berghei NUP434 and P. falciparum ortholog. Download FIG S5, PDF file, 0.6 MB. Copyright © 2022 Ambekar et al. 2022 Ambekar et al. https://creativecommons.org/licenses/by/4.0/ This content is distributed under the terms of the Creative Commons Attribution 4.0 International license.

10.1128/mbio.01815-22.10 TABLE S4 Bioinformatic analyses of Plasmodium berghei nucleoporins. Download Table S4, XLSX file, 0.01 MB. Copyright © 2022 Ambekar et al. 2022 Ambekar et al. https://creativecommons.org/licenses/by/4.0/ This content is distributed under the terms of the Creative Commons Attribution 4.0 International license.

Nup335 (amino acids 267-1,117) was modeled with 98% confidence to SMC4 (structural maintenance of chromosomes protein), a condensin that forms a heterodimer with SMC2 and together with CAPH, D2 and G is required for chromatin condensation at the prophase stage of cell division before chromatin segregation during mitosis. In Plasmodium, SMC4 is found at all proliferative stages with a characteristic centromeric localization during schizogony (60). These SMC similarities were also recognized by HHpred and the Conserved Domain Database (61). Nup335, however, showed a different, perinuclear localization.

For the remaining novel Nups, Phyre2 predicted models with less than 80% confidence and low coverage. None of the other proteins were identified in Plasmobase. In addition, this tool failed to recognize the P. berghei FG-Nups 138, 205, 221, 313, or 637, most likely due to the extremely low conservation of these proteins, not just at the primary sequence but also at the secondary and tertiary structural level. Sec13, on the other hand, was modeled with 100% confidence highlighting its evolutionary conservation and similarity to other eukaryotic Sec13 proteins (14).

Nup390 was predicted to contain 5 transmembrane domains (analysis through TMHMM) (62) (Table 1). Together with Nup221, it is one of only 2 TM domain-containing Nups known in P. berghei. Such Nups are embedded in the nuclear envelope and function as important anchors for the NPC; they also stabilize the pore complex against the sharp curvature of the nuclear membrane and play a role in NPC assembly (6).

We finally probed characteristic secondary structure elements in the newly identified Nups. They include tandem repeats of β-strands, which fold into a β-propeller, and anti-parallel α-helical repeats, which pack into α-helical solenoids (6); the latter occur in FG and linker Nups, as well as components of the nuclear basket and play a role in telomere organization and spindle pole assembly (3). We collected secondary structure predictions from PSI-blast-based secondary structure PREDiction (PSIPRED; available on PlasmoDB) and plotted them to show the confidence of prediction at each amino acid position for all predictions with confidence >5. (Fig. S6). All the candidate-Nups are predicted to contain helices and strands throughout the length of the protein without substantial disordered regions, suggesting these Nups may contribute to forming the structural scaffold of the Plasmodium NPC. These α-solenoid and β-propeller folds typically form interaction interfaces that facilitate the binding of other proteins due to their extensive solvent-accessible surfaces. Furthermore, they allow significant variation in sequence while retaining the core structure, thus optimizing protein-protein interactions for Nups (63).

10.1128/mbio.01815-22.6 FIG S6 Secondary structure prediction for novel P. berghei Nups. Protein secondary structure predictions of newly identified Nups generated from PSI-blast based secondary structure PREDiction (PSIPRED) data available on PlasmoDB was plotted using R studio. The protein length is along the x axis and the y axis indicates the confidence score for all residues with confidence >5. Orange = helices and purple = strands. Right panel shows the results of DeepCoil2 for identification of coils in the proteins. Download FIG S6, PDF file, 0.1 MB. Copyright © 2022 Ambekar et al. 2022 Ambekar et al. https://creativecommons.org/licenses/by/4.0/ This content is distributed under the terms of the Creative Commons Attribution 4.0 International license.

Finally, we analyzed the novel Nups for the presence of coiled-coil domains using Deep Coil 2 (64). Long coiled coil domains which are a few hundred residues in length, such as those found in Nup335 (Fig. S6), mediate interactions between different Nups within the NPC (6).

While potential relationships identified by remote homology detection methods in a few of the newly identified Plasmodium Nups suggest a shared ancestry, these proteins generally lack any discernible relationship to known nucleoporins, reinforcing the evolutionary divergence of the Plasmodium NPC.

### Novel Nups are essential for asexual life cycle progression.

Two of the 5 Nups are predicted to be essential for asexual life cycle progression from the large-scale PlasmoGEM forward mutagenesis screen in P. berghei (Table 1) while the remaining 3 (Nup335, 176, 269) were not targeted in this screen (65). In order to provide additional evidence for a critical role in parasite biology, we attempted to generate knockout lines for these 3 Nups using established methodologies (41). Knockouts of Nup335, 176, and 269 failed repeatedly (*n* = 3 transfections for each gene), consistent with an important or essential role for these and all known Nups in the P. berghei blood stage in maintaining a functional NPC (Table 1). Furthermore, a genome wide transposon insertional mutagenesis screen in P. falciparum did not recover any insertions within the orthologs of any of the 5 candidate Nups despite their substantial size, consistent with a vital blood stage role for each in maintaining a functional NPC (66).

### Mass-spectrometric analyses of *nup434::turboid* parasite lysates.

**FIG 4:**
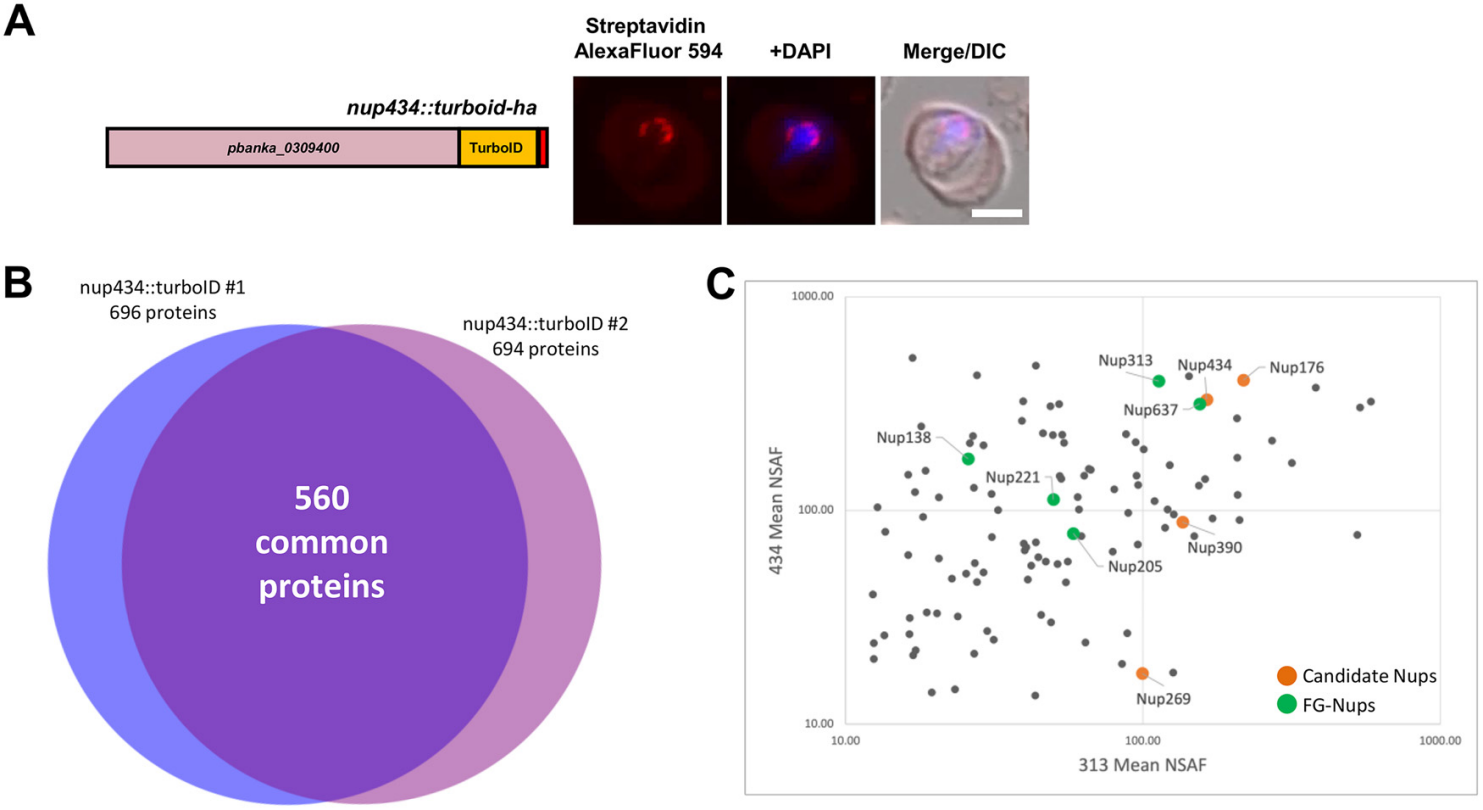
Proximity labeling with Nup434 identifies all FG-Nups and newly identified Nup candidates. (A) Schematic representation of the construct used for the experiment is shown on the left where 0.5 cm = 500 aa. Streptavidin staining shows the biotinylation of proteins in the nuclear periphery. (B) Venn diagram showing proteins identified in independent duplicates that were not observed in the wild-type control for the *nup434::turboID* MS experiment. (C) Comparison of mean normalized spectral abundance for proteins detected in both *nup313::turboID* (*x* axis) and the reciprocal *nup434::turboID* (*y* axis) experiments. All FG-Nups (green) as well as 3 of the remaining four candidate-Nups (orange) are identified in both independent experiments.

Finally, as a reciprocal experiment, we tagged the candidate inner-ring Nup434 with TurboID to validate the initial bait Nup313-Nup434 interaction. We carried out fluorescence microscopy to confirm its localization and visible biotinylation at the nuclear periphery (Fig. 4A). Mass-spectrometry analysis was carried out on 2 independent biological replicates for mutant parasites along with one wild-type control (Table S3). Excluding proteins present in the wild-type control, we identified 696 proteins in sample 1 and 694 proteins in sample 2, of which 560 were common between the duplicates (Fig. 4B). The high number of proteins identified in this experiment could be due to the variation in expression levels of Nup434 compared to Nup133, but also its orientation and position within the NPC that may facilitate greater access to the C-terminal TurboID fusion.

10.1128/mbio.01815-22.9 TABLE S3 Proteins identified in Nup434::turboID mass spectrometry experiment. Download Table S3, XLSX file, 0.1 MB. Copyright © 2022 Ambekar et al. 2022 Ambekar et al. https://creativecommons.org/licenses/by/4.0/ This content is distributed under the terms of the Creative Commons Attribution 4.0 International license.

Comparing the 2 independent mass spectrometry experiments, we identified all 5 known FG-Nups and 4 novel-Nups (Fig. 4C). In the *nup434::turboID* mass spectrometry data set, Nup313 and Nup434 were among the top 5% of proteins with mean NSAF values of 403 and 330, respectively. Another candidate, Nup176, was also within this group with a mean NSAF of 408. Forty-five proteins (12%) in our data set were ribosomal subunits, which could represent contaminants or cargo that were labeled by NPC components as they were transported between the nucleus and cytoplasm. We also detected the karyopherin beta protein in our samples, which belongs to the group of proteins responsible for the import (as importins) and export (as exportins) of macromolecules in and out of the nucleus via active transport through the NPC. Finally, given the large number of total proteins identified in this experiment, not surprisingly, we identified 38 proteins without a signal peptide and functional annotation on PlasmoDB. Since a typical eukaryotic NPC contains approximately 30 Nups, there is potential for further examination of these proteins. Altogether, we were able to identify all known NPC components as well as nucleo-cytoplasmic transport molecules in this *nup434::turboID* mass spectrometry experiment, strengthening confidence that the Nup candidates are bona fide members of the Plasmodium NPC.

## DISCUSSION

Nuclear pore proteins are key elements of eukaryotic biology. They function as essential conduits for proteins, RNA, and small molecules between the nucleus and cytoplasm, and exert key roles for gene regulation as well as karyokinesis (nuclear division) during mitosis. Yet, none of these aspects of NPC biology are understood in the malaria parasite due to the unique nature of its NPC. Despite 1.5 billion years of evolution, Nups remain well conserved across many eukaryotes including yeast, humans, trypanosomes, and even the apicomplexan Toxoplasma gondii (13), but curiously not Plasmodium. With the exception of Sec13 (12, 14) and 4 Nups containing FG-repeat modules (11, 12), efforts to identify Plasmodium NPC components through protein homology have failed (11, 67). Similarly, purification of nucleoporins through fractionation or co-immunoprecipitation, successful in many eukaryotes, has been futile (personal observations); this could be due to the large sizes of many nucleoporins we have now identified in P. berghei (Table 1). TurboID-mediated biotin labeling on the other hand emerged to be an efficient and rapid visualization method that also aids the annotation and biological classification of orphan or taxonomically restricted genes in this protozoan. We employed BioID to explore the composition of the Plasmodium NPC through proximity labeling followed by mass spectrometry and successfully identified known and previously unrecognized Nups. Past studies using BioID have already demonstrated its capability to study apicomplexan biology (23); here we extended these prospects to the rodent malaria model P. berghei NPC and tested various biotin ligase protein tags. Our data show that under physiological biotin serum levels, typically ranging from 6 to 15 ng/mL (40), TurboID, but no other biotin ligase variants, produced a reliable NPC signal that can be detected rapidly in acetone/methanol-fixed blood smears. While first-generation BioID/BirA* effectively mediates biotinylation in long-lived P. berghei gametocytes (erythrocyte-resident, non-dividing sexual precursor cells) when they are provided with 1 mM biotin in the drinking water of mice (30), we demonstrate here for the first time that labeling is possible without supplementation in asexual stage parasites in the *in vivo* mouse model using TurboID. An analysis of various BioID variants in mammalian cell culture by Roux et al. compared TurboID and miniturboID to the parental ligase, BioID, under basal as well as varying biotin concentrations in culture medium (50 μM or 500 μM) (68). TurboID was shown to be most effective, efficiently carrying out biotinylation at basal biotin levels present in culture medium, whereas miniturboID was deemed unstable, leading to a lack of protein detection; this could explain our negative results with this smallest of ligases albeit anti-tag immunofluorescence assays show full-length expression of miniturboID-tagged Nup138. Our approach reduces the complexity and time required to study the localization of the P. berghei NPC through a versatile, single-step approach that uses a fluorophore-conjugated streptavidin reagent. This acts as a fast-track method of confirming mutants and pinpointing subcellular protein localization. The results also show that it is possible to fuze a TurboID tag to proteins of varying and often considerable sizes without mislocalization or hindrance to the formation and functionality of the NPC. All known, and novel Nups identified in our mass spectrometry experiments display a localization pattern at the nuclear periphery and transcript abundance similar to that of previously characterized GFP-tagged FG-repeat Nups (12). While we could not obtain any homology information through BLAST sequence similarity searches across different eukaryotes, Phyre2 — a protein structure prediction tool (69) — revealed structural similarities of Nup434 with the considerably smaller human Nup155 and Nup133 proteins; both act as scaffold Nups that contain seven-bladed beta-propeller regions. The structural similarity of Nup335 with condensin SMC4 could indicate a role for the NPC and one its constituents during DNA replication and closed mitosis in P. berghei. Despite the overall evolutionary divergence that prevents sequence-based recognition of homology, Plasmodium Nups share basic structural features common to eukaryotic NPC components that include: FG-repeats, α-solenoids, and β-propellers. On the other hand, all nucleoporins identified in Plasmodium possess distinct features (12): these idiosyncrasies include a large poly-proline extension at the C-terminus of Sec13; a change of the widely conserved GLFG motif of many FG-repeat Nups to [SN]XFG (63 out of 95 FG repeats adhere to this motif; only 5 to the GLFG motif); and lastly the extraordinary large molecular mass of many Plasmodium Nups. The inability to identify clear structural homologs of other candidate-Nups as well as previously known FG-Nups demonstrates the advantage of proximity labeling to assign function to orphan proteins in evolutionarily distant eukaryotes and opens the door to understanding the unique evolutionary divergence of the NPC in this early-branching protozoan.

In Plasmodium, there is evidence only for the existence of nuclear pores and their dynamics during the blood stage (12, 15, 17, 70). Apart from Sec13, 2 independent studies have recently confirmed the localization of the P. berghei Nup313 ortholog *Pf*Nup313 (its actual molecular mass is predicted to be 347 kDa) to the nuclear periphery in multiple asexual and sexual stages of the parasite, and revealed an association with microtubules in multiple asexual and sexual stages of the parasite (15, 16); this interaction is also evident from our Nup313::turboID labeling experiment. Our data will allow deeper exploration of NPC dynamics during the cell division cycle in the asexual blood stage (schizogony), but also in the less accessible mosquito and vertebrate liver stage, where cytokinesis produces thousands of daughter cells in a few days (71). These proliferative stages of the malaria parasite life cycle (schizont, oocyst, and liver stage) may be susceptible to intervention of NPC formation as shown in cancer cells, which are known to be particularly sensitive to such disruption causing cell death (72).

In total, we have now confirmed 11 nucleoporins in malaria parasites; considering that eukaryotic NPCs typically contain some 30+ different Nups, several may remain to be discovered in this apicomplexan parasite. However, many of the identified Plasmodium Nups are noticeably larger than those found in humans or yeast. For example, the largest Nup in humans is FG-Nup358, or the inner ring Nup188 in yeast. Perhaps Nups in Plasmodium are in fact (yet) unrecognizable fusions of 2 or more nucleoporins as suggested for the Sec13-Nup145C fusion in P. falciparum Sec13 (14). The identification of short structural homology regions within Nup434 may lend some support to this notion.

### Conclusions.

Our study provides the first systematic analysis of enzymatic activity of various biotin ligases in the rodent malaria model P. berghei. Using the nuclear pore complex as an example, we demonstrate the value of TurboID in facilitating protein localization as well as providing insight into protein complex composition and orphan protein annotation in P. berghei, a malaria model that allows the rapid generation of mutants. Our data offer the first detailed view of the constituents of the Plasmodium nuclear pore complex and highlight the evolutionarily divergent nature of its constituent Nups, in particular from its human host.

## MATERIALS AND METHODS

### Plasmid construction.

For comparison of various biotin ligases, nucleoporin 138 (Nup138) was used as the bait protein. The C-terminal region of the gene was PCR-amplified from P. berghei genomic DNA using Q5 High-Fidelity DNA polymerase. BioID2-3XHA was obtained from Addgene (#74224) and was fused in-frame to the C-terminal end of the Nup138 resulting in plasmid pLIS0574 (Table S1) which also contains the Toxoplasma gondii DHFR selection marker. Each Nup138 tagging construct includes a 1,848 bp fragment encoding the C-terminal region of the 3,714 bp long gene to promote homologous recombination. Similarly, another fusion protein plasmid was made with the same bait protein but with a 13X GGGGS linker between the C-terminus of the protein and BioID2. TurboID-3XHA (Addgene #107171) was fused in-frame to the C-terminal end of the Nup138 using the plasmid pLIS0574 (Table S1); likewise mini-TurboID-3XHA (Addgene #107172) and BirA* (12). As a wild-type control, we used the 2.34 P. berghei clone containing none of the biotin ligases. To ensure that the nuclear periphery specific signal was from the biotin ligase fused to Nup138, we constructed a plasmid with the promoter sequence of Nup138 and TurboID as a negative control. All cloning was carried out either with T4 DNA ligase or NEBuilder HiFi (NEB).

To tag all additional FG-Nups with TurboID, we replaced the Nup138 flank in pLIS0654 with flanks for the respective genes by amplifying the C-terminal regions from the genomic locus. For visualization of candidate-Nups identified via mass spectrometry, we used pLIS0010 to tag each protein with GFP. For *nup434::turboID-ha*, we replaced the gene flank using pLIS0654.

### Transfections and maintenance of mice.

For plasmid transfection, mature schizonts were purified using MACS columns (Miltenyibiotec); transfection was performed according to established protocols (41). Transfected parasites were injected into mice intravenously using a 0.37 × 12.7 mm insulin syringe. Mutants were selected and maintained with 0.07 mg/mL pyrimethamine in drinking water provided *ad libitum*. Once parasitemia reached 4 to 6%, mice were sacrificed by heart puncture and blood collected for further experimentation as outlined below.

### Genotyping.

50-100 μL blood was lysed in RBC lysis buffer; genomic DNA was extracted from 50–100 μL cardiac puncture blood using IBI Scientific Mini Genomic DNA Kit (Blood & Cultured Cells) according to the manufacturer’s instructions. PCR genotyping for mutant parasites was carried out using primers across the homologous recombination regions. This was done such that there is a forward primer in the genomic region and reverse primer in the plasmid region at the 3′ end, and a forward primer in the plasmid and reverse primer in the genomic region at the 5′ end; bands of the correct sizes for both 3′ and 5′ confirm integration. An unmodified genomic locus and DHFR control were used as positive PCR controls. All primer sequences are available upon request. Recombinant *Taq* DNA polymerase (Invitrogen) was used for PCR and conditions were used as indicated in the protocol.

### Streptavidin staining.

Thin blood smears were made from 2 μL of blood obtained from the cardiac puncture of the infected mouse. After drying, the smears were fixed in 1:1 (vol/vol) methanol/acetone for 2 min at −20°C. Fixed smears can be stored at −20°C indefinitely. All the subsequent steps were carried out at room temperature. The fixed slides were first rehydrated in PBS for 15 min. The slides were then blocked using BlockOut - Universal Blocking Buffer (Rockland immunochemicals) for 1 h at RT. For streptavidin staining, blocked slides (see above) were incubated with Streptavidin-Alexa Fluor 594 Conjugate (Thermo Fisher) at 1:500 dilution in blocking buffer for 1 h at room temperature. To stain nuclear DNA, slides were mounted with 20 μL of Invitrogen Fluoromount-G containing DAPI. Images were taken on Zeiss Axio Observer with Axiocam 702 mono camera and Zen 2.6 Pro Software. Exposure times for each channel were kept consistent across all samples. Images were analyzed with Zeiss Zen blue software. For each line, at least 100 cells were counted and representative images are shown.

### Western blot.

For Western blots to observe the biotinylation of proteins, parasite-infected blood was collected from mice by cardiac puncture and cultured for 18h in complete RPMI supplemented with 200 μM biotin. Biotin-free media was used as a control for each mutant line as well as a wild-type control cultured under the same conditions. Parasites were collected the next day, washed, and lysed with 0.015% cold saponin. Samples were spun down, the parasite pellet was washed thrice with cold PBS, and finally lysed with RIPA and 1X protease inhibitor cocktail. After a freeze-thaw cycle, SDS sample loading buffer was added to the samples and denatured at 95°C. Samples were loaded onto a 7.5% Mini-Protean TGX precast gel for separation. The transfer was carried out overnight at 4°C. The membrane was blocked with 3% dry milk in PBST and incubated with IRDye 800-conjugated streptavidin at 1:500 for detection of biotinylated proteins. The blots were imaged with an Odyssey infrared imaging system (Li-COR Biosciences).

### Antibodies for IFA.

BlockOut-treated slides (see above) were incubated with rabbit polyclonal anti-HA antibody (ProteinTech) at 1:100 dilution or rabbit anti-myc antibody (ThermoFisher) at 1:100 in the same blocking buffer overnight at 4°C. Goat anti-rabbit highly cross absorbed secondary antibody, Alexa Fluor 594 (ThermoFisher) was then used at 1:1000 dilution for 1 h at RT. ER staining was done using rabbit polyclonal anti-PfBiP (MRA-1246, BEI resources) at 1:500 dilution. Fluorophore conjugated WGA and AAL were used at 1:500 dilutions. For localization of candidate Nups, rabbit polyclonal anti-GFP (enQuire bioreagents) antibody was used at 1:100 dilution. For all IFA experiments, nuclear DNA was stained with DAPI present in Invitrogen Fluoromount-G.

### Preparation of protein sample and proteomics by mass spectrometry.

To identify novel nuclear pore components, we carried out streptavidin magnetic bead pull-down followed by mass spectrometry (MS) using protein lysates prepared from the TurboID fusion proteins. In brief, following cardiac puncture of infected mice, parasites were grown in culture O/N with RPMI 1640 media (Sigma-Aldrich) to generate a homogenous schizont population. Parasites were purified using a MACS column to remove uninfected RBCs. Following elution with RPMI, erythrocytes were lysed with cold 0.05% saponin; parasites were then washed twice with cold 1 x PBS and pelleted at 4000G at 4°C. Cells were finally lysed with 8M Urea with 1 mM DTT containing Halt protease inhibitor cocktail (Thermo Scientific). The sample was incubated with 50 μL magnetic streptavidin beads at 4°C overnight on a rotator to bind biotinylated proteins. The following day, beads were collected using a magnetic column and washed thrice on a rotator at room temperature for 5 min with a buffer containing 8M Urea, 50 mM Tris, and 0.01% Triton X-100. A final wash was done with wash buffer without Triton X-100 and the beads were diluted in 8M Urea. Mass spectrometric analysis (LC-MS/MS) was performed at the UCLA proteomics facility and ISU protein facility.

The proteins bound to streptavidin beads were reduced and alkylated via sequential 20-minute incubations of 5 mM TCEP and 10 mM iodoacetamide at room temperature in the dark while being mixed at 1200 rpm in an Eppendorf thermomixer. Proteins were then 1digested by the addition of 0.1 μg Lys-C (FUJIFILM Wako Pure Chemical Corporation, 125-05061) and 0.8 μg Trypsin (Thermo Scientific, 90057) while shaking 37°C overnight. The digested samples were quenched by addition of formic acid, desalted, and then resuspended in 15 μL of 5% formic acid for analysis by LC-MS/MS.

Peptide samples were separated on a 75uM ID x 25 cm C18 column and eluted directly into a Thermo Orbitrap Fusion Lumos mass spectrometer. MS spectra were acquired by Data Dependent Acquisition (DDA). Database search was performed by using ProLuCID (73) and DTASelect2 (74, 75) implemented in Integrated Proteomics Pipeline IP2 (Integrated Proteomics Applications) and searched against P. berghei database available on PlasmoDB. A PSM-level false positive rate was set at less than 1% as estimated by a target-decoy database competition strategy, protein and peptide identifications were filtered by DTASelect2 and a minimum of 2 unique peptides per protein are required for confident protein identification.

### Bioinformatic analyses.

All DNA and protein sequences used in bioinformatic analyses were acquired from PlasmoDB (release 51, 16 March 2021 or earlier) (20). To calculate the enrichment of Nups, a hypergeometric test developed by the Graeber Lab was used (https://systems.crumP.ucla.edu/hypergeometric/index.php). Phyre2 was used with default parameters for function prediction of the proteins by homology modeling (69). The PANTHER classification system was used to analyze mass spectrometry hits by protein class and biological function. First, PBANKA IDs from Table 1 were entered into Uniprot via the Retrieve/ID mapping option and converted into UniProtKB IDs using gene names. The Uniprot accession numbers were then plugged into PANTHER (http://www.pantherdb.org/) (Mi et al., 2019) and P. berghei was chosen as the reference proteome to carry out the functional classification. Proteins were grouped into the following categories: molecular function, biological process, cellular component, protein class, and pathway.

### Data availability.

The data sets supporting the conclusions of this article are included within the article and its additional files: Table S2 and Table S3. The mass spectrometry proteomics data are deposited in ProteomeXchange Consortium via the PRIDE partner repository with the accession number PXD034491.

## References

[1] Arendsee ZW, Li L, Wurtele ES. 2014. Coming of age: orphan genes in plants. Trends Plant Sci 19:698–708. doi:10.1016/j.tplants.2014.07.003. 25151064

[2] Vakirlis N, Carvunis AR, McLysaght A. 2020. Synteny-based analyses indicate that sequence divergence is not the main source of orphan genes. Elife 9:e53500. doi:10.7554/eLife.53500. 32066524 PMC7028367

[3] Grossman E, Medalia O, Zwerger M. 2012. Functional architecture of the nuclear pore complex. Annu Rev Biophys 41:557–584. doi:10.1146/annurev-biophys-050511-102328. 22577827

[4] Field MC, Koreny L, Rout MP. 2014. Enriching the pore: splendid complexity from humble origins. Traffic 15:141–156. doi:10.1111/tra.12141. 24279500 PMC3906644

[5] Beck M, Hurt E. 2017. The nuclear pore complex: understanding its function through structural insight. Nat Rev Mol Cell Biol 18:73–89. doi:10.1038/nrm.2016.147. 27999437

[6] Lin DH, Hoelz A. 2019. The structure of the nuclear pore complex (an update). Annu Rev Biochem 88:725–783. doi:10.1146/annurev-biochem-062917-011901. 30883195 PMC6588426

[7] Ganesan SJ, Feyder MJ, Chemmama IE, Fang F, Rout MP, Chait BT, Shi Y, Munson M, Sali A. 2020. Integrative structure and function of the yeast exocyst complex. Protein Sci 29:1486–1501. doi:10.1002/pro.3863. 32239688 PMC7255525

[8] DeGrasse JA, DuBois KN, Devos D, Siegel TN, Sali A, Field MC, Rout MP, Chait BT. 2009. Evidence for a shared nuclear pore complex architecture that is conserved from the last common eukaryotic ancestor. Mol Cell Proteomics 8:2119–2130. doi:10.1074/mcp.M900038-MCP200. 19525551 PMC2742445

[9] Degrasse JA, Devos D. 2010. A functional proteomic study of the *Trypanosoma brucei* nuclear pore complex: an informatic strategy. Methods Mol Biol 673:231–238. doi:10.1007/978-1-60761-842-3_15. 20835803

[10] Obado SO, Brillantes M, Uryu K, Zhang W, Ketaren NE, Chait BT, Field MC, Rout MP. 2016. Interactome mapping reveals the evolutionary history of the nuclear pore complex. PLoS Biol 14:e1002365. doi:10.1371/journal.pbio.1002365. 26891179 PMC4758718

[11] Mans BJ, Anantharaman V, Aravind L, Koonin EV. 2004. Comparative genomics, evolution and origins of the nuclear envelope and nuclear pore complex. Cell Cycle 3:1612–1637. doi:10.4161/cc.3.12.1316. 15611647

[12] Kehrer J, Kuss C, Andres-Pons A, Reustle A, Dahan N, Devos D, Kudryashev M, Beck M, Mair GR, Frischknecht F. 2018. Nuclear pore complex components in the malaria parasite *Plasmodium berghei*. Sci Rep 8:11249. doi:10.1038/s41598-018-29590-5. 30050042 PMC6062611

[13] Courjol F, Mouveaux T, Lesage K, Saliou J-M, Werkmeister E, Bonabaud M, Rohmer M, Slomianny C, Lafont F, Gissot M. 2017. Characterization of a nuclear pore protein sheds light on the roles and composition of the *Toxoplasma gondii* nuclear pore complex. Cell Mol Life Sci 74:2107–2125. doi:10.1007/s00018-017-2459-3. 28138739 PMC11107709

[14] Dahan-Pasternak N, Nasereddin A, Kolevzon N, Pe'er M, Wong W, Shinder V, Turnbull L, Whitchurch CB, Elbaum M, Gilberger TW, Yavin E, Baum J, Dzikowski R. 2013. PfSec13 is an unusual chromatin-associated nucleoporin of *Plasmodium falciparum* that is essential for parasite proliferation in human erythrocytes. J Cell Sci 126:3055–3069. doi:10.1242/jcs.122119. 23687383

[15] Boltryk SD, Passecker A, Alder A, Carrington E, van de Vegte-Bolmer M, van Gemert G-J, van der Starre A, Beck H-P, Sauerwein RW, Kooij TWA, Brancucci NMB, Proellochs NI, Gilberger T-W, Voss TS. 2021. CRISPR/Cas9-engineered inducible gametocyte producer lines as a valuable tool for *Plasmodium falciparum* malaria transmission research. Nat Commun 12:3055–3069. doi:10.1038/s41467-021-24954-4. 34376675 PMC8355313

[16] Simon CS, Funaya C, Bauer J, Voβ Y, Machado M, Penning A, Klaschka D, Cyrklaff M, Kim J, Ganter M, Guizetti J. 2021. An extended DNA-free intranuclear compartment organizes centrosome microtubules in malaria parasites. Life Sci Alliance 4:e202101199. doi:10.26508/lsa.202101199. 34535568 PMC8473725

[17] Weiner A, Dahan-Pasternak N, Shimoni E, Shinder V, von Huth P, Elbaum M, Dzikowski R. 2011. 3D nuclear architecture reveals coupled cell cycle dynamics of chromatin and nuclear pores in the malaria parasite Plasmodium falciparum. Cell Microbiol 13:967–977. doi:10.1111/j.1462-5822.2011.01592.x. 21501361

[18] Deitsch K, Duraisingh M, Dzikowski R, Gunasekera A, Khan S, Le Roch K, Llinás M, Mair G, McGovern V, Roos D, Shock J, Sims J, Wiegand R, Winzeler E. 2007. Mechanisms of gene regulation in *Plasmodium*. Am J Trop Med Hyg 77:201–208. doi:10.4269/ajtmh.2007.77.201. 17690387

[19] Guizetti J, Martins RM, Guadagnini S, Claes A, Scherf A. 2013. Nuclear pores and perinuclear expression sites of var and ribosomal DNA genes correspond to physically distinct regions in *Plasmodium falciparum*. Eukaryot Cell 12:697–702. doi:10.1128/EC.00023-13. 23475702 PMC3647773

[20] Aurrecoechea C, Brestelli J, Brunk BP, Dommer J, Fischer S, Gajria B, Gao X, Gingle A, Grant G, Harb OS, Heiges M, Innamorato F, Iodice J, Kissinger JC, Kraemer E, Li W, Miller JA, Nayak V, Pennington C, Pinney DF, Roos DS, Ross C, Stoeckert CJ, Treatman C, Wang H. 2009. PlasmoDB: a functional genomic database for malaria parasites. Nucleic Acids Res 37:D539–D543. doi:10.1093/nar/gkn814. 18957442 PMC2686598

[21] Mair GR, Lasonder E, Garver LS, Franke-Fayard BMD, Carret CK, Wiegant JCAG, Dirks RW, Dimopoulos G, Janse CJ, Waters AP. 2010. Universal features of post-transcriptional gene regulation are critical for Plasmodium zygote development. PLoS Pathog 6:e1000767. doi:10.1371/journal.ppat.1000767. 20169188 PMC2820534

[22] Muñoz EE, Hart KJ, Walker MP, Kennedy MF, Shipley MM, Lindner SE. 2017. ALBA4 modulates its stage-specific interactions and specific mRNA fates during Plasmodium yoelii growth and transmission. Mol Microbiol 6:266–284. doi:10.1111/mmi.13762. PMC5688949 28787542

[23] Kimmel J, Kehrer J, Frischknecht F, Spielmann T. 2021. Proximity-dependent biotinylation approaches to study apicomplexan biology. Mol Microbiol 7:553–568. doi:10.1111/mmi.14815. 34587292

[24] Bosch JA, Chen CL, Perrimon N. 2021. Proximity-dependent labeling methods for proteomic profiling in living cells: an update. Wiley Interdiscip Rev Dev Biol 10:e392. doi:10.1002/wdev.392. 32909689 PMC8142282

[25] Li P, Li J, Wang L, Di L-J. 2017. Proximity labeling of interacting proteins: application of BioID as a discovery tool. Proteomics 17:1700002. doi:10.1002/pmic.201700002. 28271636

[26] Qin W, Cho KF, Cavanagh PE, Ting AY. 2021. Deciphering molecular interactions by proximity labeling. Nat Methods 18:133–143. doi:10.1038/s41592-020-01010-5. 33432242 PMC10548357

[27] Roux KJ, Kim DI, Raida M, Burke B. 2012. A promiscuous biotin ligase fusion protein identifies proximal and interacting proteins in mammalian cells. J Cell Biol 196:801–810. doi:10.1083/jcb.201112098. 22412018 PMC3308701

[28] Lam SS, Martell JD, Kamer KJ, Deerinck TJ, Ellisman MH, Mootha VK, Ting AY. 2015. Directed evolution of APEX2 for electron microscopy and proximity labeling. Nat Methods 12:51–54. doi:10.1038/nmeth.3179. 25419960 PMC4296904

[29] Kim DI, Roux KJ. 2016. Filling the void: proximity-based labeling of proteins in living cells. Trends Cell Biol 26:804–817. doi:10.1016/j.tcb.2016.09.004. 27667171 PMC5077660

[30] Kehrer J, Frischknecht F, Mair GR. 2016. Proteomic analysis of the *Plasmodium berghei* gametocyte egressome and vesicular bioID of osmiophilic body proteins identifies merozoite TRAP-like protein (MTRAP) as an essential factor for parasite transmission. Mol Cell Proteomics 15:2852–2862. doi:10.1074/mcp.M116.058263. 27371728 PMC5013303

[31] Khosh-Naucke M, Becker J, Mesén-Ramírez P, Kiani P, Birnbaum J, Fröhlke U, Jonscher E, Schlüter H, Spielmann T. 2018. Identification of novel parasitophorous vacuole proteins in *P. falciparum* parasites using BioID. Int J Med Microbiol 308:13–24. doi:10.1016/j.ijmm.2017.07.007. 28784333

[32] Schnider CB, Bausch-Fluck D, Brühlmann F, Heussler VT, Burda P-C. 2018. BioID reveals novel proteins of the *Plasmodium* parasitophorous vacuole membrane. mSphere 3:e00522-17. doi:10.1128/mSphere.00522-17. PMC5784244 29404413

[33] Nessel T, Beck JM, Rayatpisheh S, Jami-Alahmadi Y, Wohlschlegel JA, Goldberg DE, Beck JR. 2020. EXP1 is required for organisation of EXP2 in the intraerythrocytic malaria parasite vacuole. Cell Microbiol 22:e13168. doi:10.1111/cmi.13168. 31990132 PMC7138706

[34] Miyazaki S, Chitama B-YA, Kagaya W, Lucky AB, Zhu X, Yahata K, Morita M, Takashima E, Tsuboi T, Kaneko O. 2021. *Plasmodium falciparum* SURFIN4.1 forms an intermediate complex with PTEX components and Pf113 during export to the red blood cell. Parasitol Int 83:102358. doi:10.1016/j.parint.2021.102358. 33901679

[35] Boucher MJ, Ghosh S, Zhang L, Lal A, Jang SW, Ju A, Zhang S, Wang X, Ralph SA, Zou J, Elias JE, Yeh E. 2018. Integrative proteomics and bioinformatic prediction enable a high-confidence apicoplast proteome in malaria parasites. PLoS Biol 16:e2005895. doi:10.1371/journal.pbio.2005895. 30212465 PMC6155542

[36] Wichers JS, Wunderlich J, Heincke D, Pazicky S, Strauss J, Schmitt M, Kimmel J, Wilcke L, Scharf S, von Thien H, Burda PC, Spielmann T, Löw C, Filarsky M, Bachmann A, Gilberger TW. 2021. Identification of novel inner membrane complex and apical annuli proteins of the malaria parasite *Plasmodium falciparum*. Cell Microbiol 23:e13341. doi:10.1111/cmi.13341. 33830607

[37] Kim DI, Jensen SC, Noble KA, Kc B, Roux KH, Motamedchaboki K, Roux KJ. 2016. An improved smaller biotin ligase for BioID proximity labeling. Mol Biol Cell 27:1188–1196. doi:10.1091/mbc.E15-12-0844. 26912792 PMC4831873

[38] Branon TC, Bosch JA, Sanchez AD, Udeshi ND, Svinkina T, Carr SA, Feldman JL, Perrimon N, Ting AY. 2018. Efficient proximity labeling in living cells and organisms with TurboID. Nat Biotechnol 36:880–887. doi:10.1038/nbt.4201. 30125270 PMC6126969

[39] Dellibovi-Ragheb TA, Jhun H, Goodman CD, Walters MS, Ragheb DRT, Matthews KA, Rajaram K, Mishra S, McFadden GI, Sinnis P, Prigge ST. 2018. Host biotin is required for liver stage development in malaria parasites. Proc Natl Acad Sci USA 115:E2604–E2613. 29483266 10.1073/pnas.1800717115 PMC5856565

[40] Carfrae LA, MacNair CR, Brown CM, Tsai CN, Weber BS, Zlitni S, Rao VN, Chun J, Junop MS, Coombes BK, Brown ED. 2020. Mimicking the human environment in mice reveals that inhibiting biotin biosynthesis is effective against antibiotic-resistant pathogens. Nat Microbiol 5:93–101. doi:10.1038/s41564-019-0595-2. 31659298

[41] Janse CJ, Ramesar J, Waters AP. 2006. High-efficiency transfection and drug selection of genetically transformed blood stages of the rodent malaria parasite *Plasmodium berghei*. Nat Protoc 1:346–356. doi:10.1038/nprot.2006.53. 17406255

[42] Jevtić P, Schibler AC, Wesley CC, Pegoraro G, Misteli T, Levy DL. 2019. The nucleoporin ELYS regulates nuclear size by controlling NPC number and nuclear import capacity. EMBO Rep 20:e47283. doi:10.15252/embr.201847283. 31085625 PMC6549122

[43] Stavru F, Hülsmann BB, Spang A, Hartmann E, Cordes VC, Görlich D. 2006. NDC1: a crucial membrane-integral nucleoporin of metazoan nuclear pore complexes. J Cell Biol 173:509–519. doi:10.1083/jcb.200601001. 16702233 PMC2063861

[44] Li B, Kohler JJ. 2014. Glycosylation of the nuclear pore. Traffic 15:347–361. and doi:10.1111/tra.12150. 24423194 PMC4001855

[45] Bandini G, Haserick JR, Motari E, Ouologuem DT, Lourido S, Roos DS, Costello CE, Robbins PW, Samuelson J. 2016. O-fucosylated glycoproteins form assemblies in close proximity to the nuclear pore complexes of *Toxoplasma gondii*. Proc Natl Acad Sci USA 113:11567–11572. doi:10.1073/pnas.1613653113. 27663739 PMC5068260

[46] Janse CJ, Franke-Fayard B, Mair GR, Ramesar J, Thiel C, Engelmann S, Matuschewski K, van Gemert GJ, Sauerwein RW, Waters AP. 2006. High efficiency transfection of *Plasmodium berghei* facilitates novel selection procedures. Mol Biochem Parasitol 145:60–70. doi:10.1016/j.molbiopara.2005.09.007. 16242190

[47] Janse CJ, Waters AP. 1995. *Plasmodium berghei:* the application of cultivation and purification techniques to molecular studies of malaria parasites. Parasitol Today 11:138–143. doi:10.1016/0169-4758(95)80133-2. 15275357

[48] Nguyen Ba AN, Pogoutse A, Provart N, Moses AM. 2009. NLStradamus: a simple hidden markov model for nuclear localization signal prediction. BMC Bioinformatics 10:202. doi:10.1186/1471-2105-10-202. 19563654 PMC2711084

[49] Oehring SC, Woodcroft BJ, Moes S, Wetzel J, Dietz O, Pulfer A, Dekiwadia C, Maeser P, Flueck C, Witmer K, Brancucci NMB, Niederwieser I, Jenoe P, Ralph SA, Voss TS. 2012. Organellar proteomics reveals hundreds of novel nuclear proteins in the malaria parasite *Plasmodium falciparum*. Genome Biol 13:R108. doi:10.1186/gb-2012-13-11-r108. 23181666 PMC4053738

[50] Mi H, Ebert D, Muruganujan A, Mills C, Albou L-P, Mushayamaha T, Thomas PD. 2021. PANTHER version 16: a revised family classification, tree-based classification tool, enhancer regions and extensive API. Nucleic Acids Res 49:D394–D403. doi:10.1093/nar/gkaa1106. 33290554 PMC7778891

[51] Baßler J, Hurt E. 2019. Eukaryotic ribosome assembly. Annu Rev Biochem 88:281–306. doi:10.1146/annurev-biochem-013118-110817. 30566372

[52] Pena C, Hurt E, Panse VG. 2017. Eukaryotic ribosome assembly, transport and quality control. Nat Struct Mol Biol 24:689–699. doi:10.1038/nsmb.3454. 28880863

[53] Chow K-H, Elgort S, Dasso M, Powers MA, Ullman KS. 2014. The SUMO proteases SENP1 and SENP2 play a critical role in nucleoporin homeostasis and nuclear pore complex function. Mol Biol Cell 25:160–168. doi:10.1091/mbc.E13-05-0256. 24196834 PMC3873886

[54] Kramarz K, Schirmeisen K, Boucherit V, Ait Saada A, Lovo C, Palancade B, Freudenreich C, Lambert SAE. 2020. The nuclear pore primes recombination-dependent DNA synthesis at arrested forks by promoting SUMO removal. Nat Commun 11:5643. doi:10.1038/s41467-020-19516-z. 33159083 PMC7648084

[55] Holzer K, Ori A, Cooke A, Dauch D, Drucker E, Riemenschneider P, Andres-Pons A, DiGuilio AL, Mackmull M-T, Baßler J, Roessler S, Breuhahn K, Zender L, Glavy JS, Dombrowski F, Hurt E, Schirmacher P, Beck M, Singer S. 2019. Nucleoporin Nup155 is part of the p53 network in liver cancer. Nat Commun 10:2147. doi:10.1038/s41467-019-10133-z. 31089132 PMC6517424

[56] Amlacher S, Sarges P, Flemming D, van Noort V, Kunze R, Devos DP, Arumugam M, Bork P, Hurt E. 2011. Insight into structure and assembly of the nuclear pore complex by utilizing the genome of a eukaryotic thermophile. Cell 146:277–289. doi:10.1016/j.cell.2011.06.039. 21784248

[57] Bernardes J, Vaquero C, Carbone A. 2017. Plasmobase: a comparative database of predicted domain architectures for *Plasmodium* genomes. Malar J 16:241. doi:10.1186/s12936-017-1887-8. 28592293 PMC5463329

[58] Zimmermann L, Stephens A, Nam S-Z, Rau D, Kübler J, Lozajic M, Gabler F, Söding J, Lupas AN, Alva V. 2018. A completely reimplemented MPI bioinformatics toolkit with a new HHpred server at its core. J Mol Biol 430:2237–2243. doi:10.1016/j.jmb.2017.12.007. 29258817

[59] Gabler F, Nam S-Z, Till S, Mirdita M, Steinegger M, Söding J, Lupas AN, Alva V. 2020. Protein sequence analysis using the MPI bioinformatics toolkit. Curr Protoc Bioinformatics 72:e108. doi:10.1002/cpbi.108. 33315308

[60] Pandey R, Abel S, Boucher M, Wall RJ, Zeeshan M, Rea E, Freville A, Lu XM, Brady D, Daniel E, Stanway RR, Wheatley S, Batugedara G, Hollin T, Bottrill AR, Gupta D, Holder AA, Le Roch KG, Tewari R. 2020. *Plasmodium* condensin core subunits SMC2/SMC4 mediate atypical mitosis and are essential for parasite proliferation and transmission. Cell Rep 30:1883–1897. doi:10.1016/j.celrep.2020.01.033. 32049018 PMC7016506

[61] Lu S, Wang J, Chitsaz F, Derbyshire MK, Geer RC, Gonzales NR, Gwadz M, Hurwitz DI, Marchler GH, Song JS, Thanki N, Yamashita RA, Yang M, Zhang D, Zheng C, Lanczycki CJ, Marchler-Bauer A. 2020. CDD/SPARCLE: the conserved domain database in 2020. Nucleic Acids Res 48:D265–D268. doi:10.1093/nar/gkz991. 31777944 PMC6943070

[62] Sonnhammer EL, von Heijne G, Krogh A. 1998. A hidden Markov model for predicting transmembrane helices in protein sequences. Proc Int Conf Intell Syst Mol Biol 6:175–182. 9783223

[63] Devos D, Dokudovskaya S, Williams R, Alber F, Eswar N, Chait BT, Rout MP, Sali A. 2006. Simple fold composition and modular architecture of the nuclear pore complex. Proc Natl Acad Sci USA 103:2172–2177. doi:10.1073/pnas.0506345103. 16461911 PMC1413685

[64] Ludwiczak J, Winski A, Szczepaniak K, Alva V, Dunin-Horkawicz S. 2019. DeepCoil-a fast and accurate prediction of coiled-coil domains in protein sequences. Bioinformatics 35:2790–2795. doi:10.1093/bioinformatics/bty1062. 30601942

[65] Bushell E, Gomes AR, Sanderson T, Anar B, Girling G, Herd C, Metcalf T, Modrzynska K, Schwach F, Martin RE, Mather MW, McFadden GI, Parts L, Rutledge GG, Vaidya AB, Wengelnik K, Rayner JC, Billker O. 2017. Functional profiling of a *Plasmodium* genome reveals an abundance of essential genes. Cell 170:260–272. doi:10.1016/j.cell.2017.06.030. 28708996 PMC5509546

[66] Zhang M, Wang C, Otto TD, Oberstaller J, Liao X, Adapa SR, Udenze K, Bronner IF, Casandra D, Mayho M, Brown J, Li S, Swanson J, Rayner JC, Jiang RHY, Adams JH. 2018. Uncovering the essential genes of the human malaria parasite *Plasmodium falciparum* by saturation mutagenesis. Science 360. doi:10.1126/science.aap7847. PMC6360947 29724925

[67] Neumann N, Lundin D, Poole AM. 2010. Comparative genomic evidence for a complete nuclear pore complex in the last eukaryotic common ancestor. PLoS One 5:e13241. doi:10.1371/journal.pone.0013241. 20949036 PMC2951903

[68] May DG, Scott KL, Campos AR, Roux KJ. 2020. Comparative application of BioID and TurboID for protein-proximity biotinylation. Cells 9:1070. doi:10.3390/cells9051070. PMC7290721 32344865

[69] Kelley LA, Mezulis S, Yates CM, Wass MN, Sternberg MJE. 2015. The Phyre2 web portal for protein modeling, prediction and analysis. Nat Protoc 10:845–858. doi:10.1038/nprot.2015.053. 25950237 PMC5298202

[70] Simon CS, Funaya C, Bauer J, Voß Y, Machado M, Penning A, Klaschka D, Cyrklaff M, Kim J, Ganter M, Guizetti J. 2021. An extended DNA-free intranuclear compartment organizes centrosomal microtubules in *Plasmodium falciparum*. bioRxiv 10.1101/2021.03.12.435157. PMC8473725 34535568

[71] Prudencio M, Rodriguez A, Mota MM. 2006. The silent path to thousands of merozoites: the *Plasmodium* liver stage. Nat Rev Microbiol 4:849–856. doi:10.1038/nrmicro1529. 17041632

[72] Sakuma S, Raices M, Borlido J, Guglielmi V, Zhu EYS, D'Angelo MA. 2021. Inhibition of nuclear pore complex formation selectively induces cancer cell death. Cancer Discov 11:176–193. doi:10.1158/2159-8290.CD-20-0581. 32988961 PMC8004544

[73] Xu T, Park SK, Venable JD, Wohlschlegel JA, Diedrich JK, Cociorva D, Lu B, Liao L, Hewel J, Han X, Wong CCL, Fonslow B, Delahunty C, Gao Y, Shah H, Yates JR. 2015. ProLuCID: an improved SEQUEST-like algorithm with enhanced sensitivity and specificity. J Proteomics 129:16–24. doi:10.1016/j.jprot.2015.07.001. 26171723 PMC4630125

[74] Tabb DL, McDonald WH, Yates JR, III. 2002. DTASelect and Contrast: tools for assembling and comparing protein identifications from shotgun proteomics. J Proteome Res 1:21–26. doi:10.1021/pr015504q. 12643522 PMC2811961

[75] Cociorva D, L Tabb D, Yates JR. 2007. Validation of tandem mass spectrometry database search results using DTASelect. Curr Protoc Bioinformatics 13:3.4.1–13.4.14. doi:10.1002/0471250953.bi1304s16. 18428785

[76] Otto TD, Böhme U, Jackson AP, Hunt M, Franke-Fayard B, Hoeijmakers WAM, Religa AA, Robertson L, Sanders M, Ogun SA, Cunningham D, Erhart A, Billker O, Khan SM, Stunnenberg HG, Langhorne J, Holder AA, Waters AP, Newbold CI, Pain A, Berriman M, Janse CJ. 2014. A comprehensive evaluation of rodent malaria parasite genomes and gene expression. BMC Biol 12:86. 25359557 10.1186/s12915-014-0086-0 PMC4242472

