# Supplementary material for: TurboID Identification of Evolutionarily Divergent Components of the Nuclear Pore Complex in the Malaria Model Plasmodium berghei
Source: mBio. 2022 Aug 30;13(5):e01815-22. doi: 10.1128/mbio.01815-22 (PMC9601220; doi:10.1128/mbio.01815-22)
Supplement: Figure S1 [file mbio.01815-22-s0004.pdf]

Figure S1

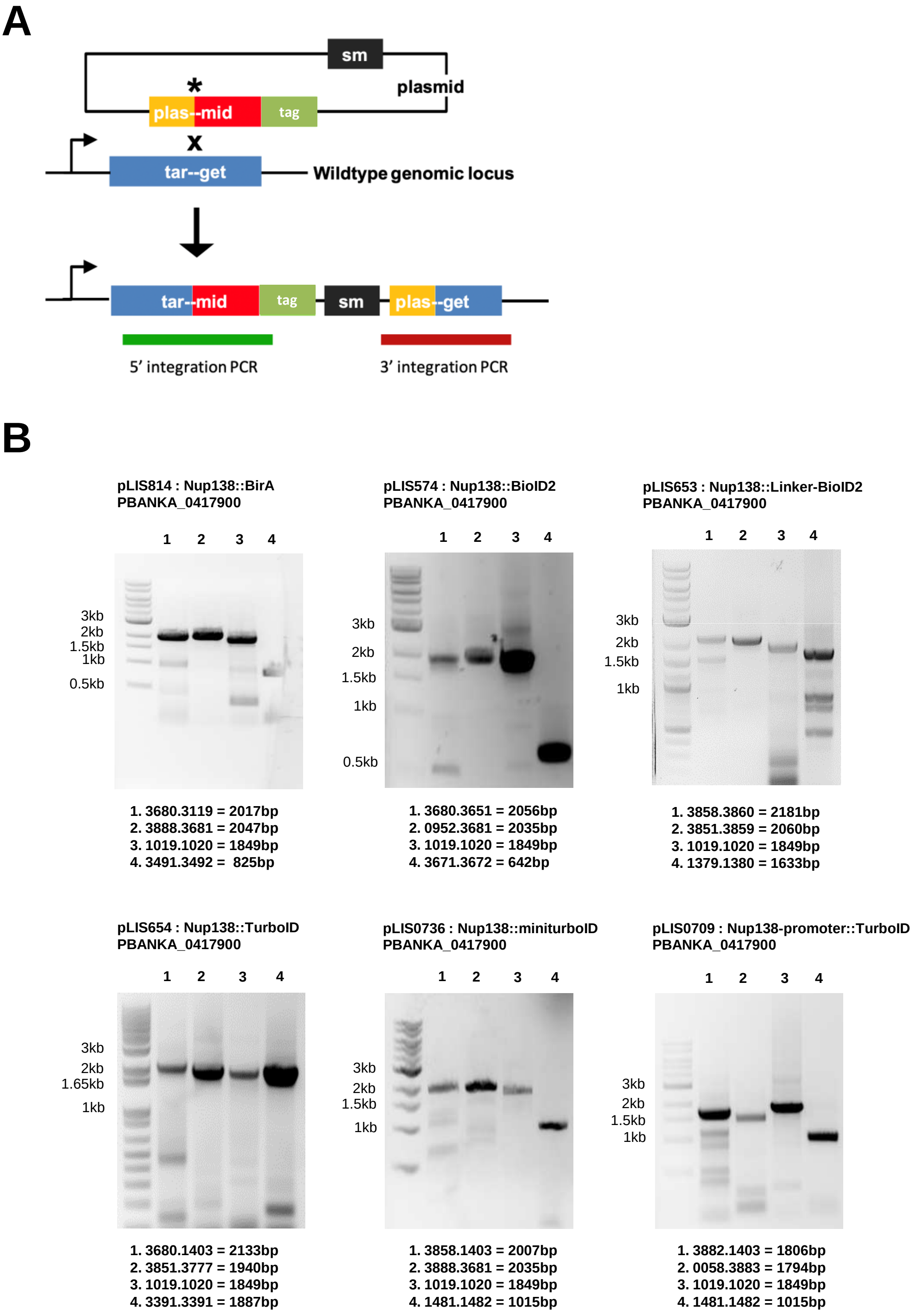
A
sm
plasmid
plas--mid
tag
tar--get
Wildtype genomic locus
tar--mid
tag
sm
plas--get
5' integration PCR
3' integration PCR
B
pLIS814 : Nup138::BirA
PBANKA_0417900
1 2 3 4
3kb
2kb
1.5kb
1kb
0.5kb
1. 3680.3119 = 2017bp
2. 3888.3681 = 2047bp
3. 1019.1020 = 1849bp
4. 3491.3492 = 825bp
pLIS574 : Nup138::BioID2
PBANKA_0417900
1 2 3 4
3kb
2kb
1.5kb
1kb
0.5kb
1. 3680.3651 = 2056bp
2. 0952.3681 = 2035bp
3. 1019.1020 = 1849bp
4. 3671.3672 = 642bp
pLIS653 : Nup138::Linker-BioID2
PBANKA_0417900
1 2 3 4
3kb
2kb
1.5kb
1kb
1. 3858.3860 = 2181bp
2. 3851.3859 = 2060bp
3. 1019.1020 = 1849bp
4. 1379.1380 = 1633bp
pLIS654 : Nup138::TurboID
PBANKA_0417900
1 2 3 4
3kb
2kb
1.65kb
1kb
1. 3680.1403 = 2133bp
2. 3851.3777 = 1940bp
3. 1019.1020 = 1849bp
4. 3391.3391 = 1887bp
pLIS0736 : Nup138::miniturboID
PBANKA_0417900
1 2 3 4
3kb
2kb
1.5kb
1kb
1. 3858.1403 = 2007bp
2. 3888.3681 = 2035bp
3. 1019.1020 = 1849bp
4. 1481.1482 = 1015bp
pLIS0709 : Nup138-promoter::TurboID
PBANKA_0417900
1 2 3 4
3kb
2kb
1.5kb
1kb
1. 3882.1403 = 1806bp
2. 0058.3883 = 1794bp
3. 1019.1020 = 1849bp
4. 1481.1482 = 1015bp

C

pLIS0705: Nup205::turboID
PBANKA_1140100

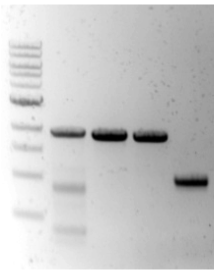

1. 3081.1403 = 1989bp
2. 3888.3082 = 1913bp
3. 1019.1020 = 1849bp
4. 1481.1482 = 1015bp

pLIS0710: Nup221::turboID
PBANKA_0416300

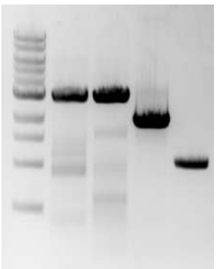

1. 3120.1403 = 2938bp
2. 3888.3659 = 2822bp
3. 1019.1020 = 1849bp
4. 1481.1482 = 1015bp

pLIS0706: Nup313::turboID
PBANKA_1310200

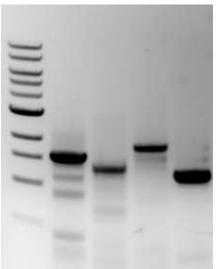

1. 3078.1403 = 1608bp
2. 3851.1447 = 1399bp
3. 1019.1020 = 1849bp
4. 1445.1446 = 1210bp

pLIS0760: Nup637::turboID
PBANKA_0107600

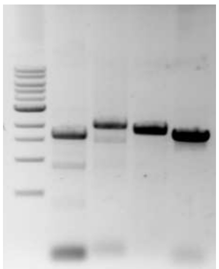

1. 3080.1403 = 1707bp
2. 3888.0745 = 2129bp
3. 1019.1020 = 1849bp
4. 3466.3467 = 1751bp

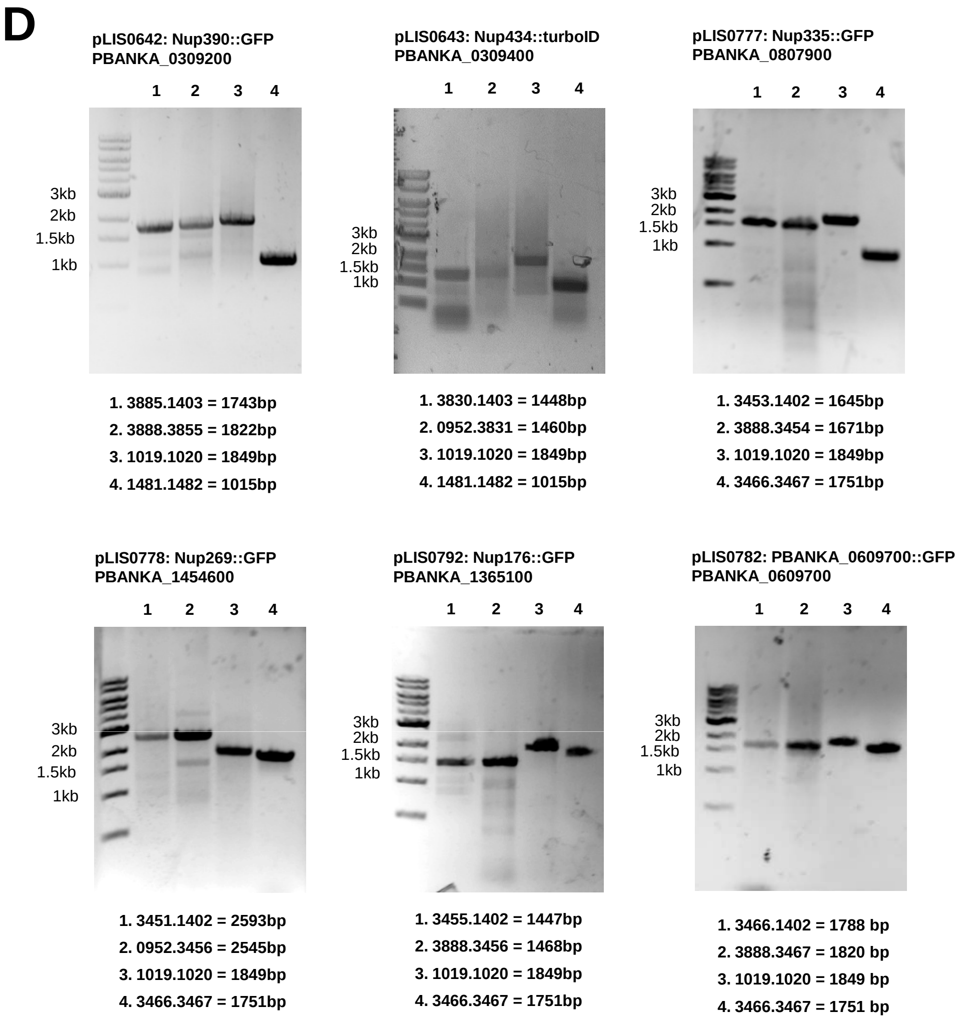
D
pLIS0642: Nup390::GFP
PBANKA_0309200
1 2 3 4
3kb
2kb
1.5kb
1kb
1. 3885.1403 = 1743bp
2. 3888.3855 = 1822bp
3. 1019.1020 = 1849bp
4. 1481.1482 = 1015bp
pLIS0643: Nup434::turboID
PBANKA_0309400
1 2 3 4
3kb
2kb
1.5kb
1kb
1. 3830.1403 = 1448bp
2. 0952.3831 = 1460bp
3. 1019.1020 = 1849bp
4. 1481.1482 = 1015bp
pLIS0777: Nup335::GFP
PBANKA_0807900
1 2 3 4
3kb
2kb
1.5kb
1kb
1. 3453.1402 = 1645bp
2. 3888.3454 = 1671bp
3. 1019.1020 = 1849bp
4. 3466.3467 = 1751bp
pLIS0778: Nup269::GFP
PBANKA_1454600
1 2 3 4
3kb
2kb
1.5kb
1kb
1. 3451.1402 = 2593bp
2. 0952.3456 = 2545bp
3. 1019.1020 = 1849bp
4. 3466.3467 = 1751bp
pLIS0792: Nup176::GFP
PBANKA_1365100
1 2 3 4
3kb
2kb
1.5kb
1kb
1. 3455.1402 = 1447bp
2. 3888.3456 = 1468bp
3. 1019.1020 = 1849bp
4. 3466.3467 = 1751bp
pLIS0782: PBANKA_0609700::GFP
PBANKA_0609700
1 2 3 4
3kb
2kb
1.5kb
1kb
1. 3466.1402 = 1788 bp
2. 3888.3467 = 1820 bp
3. 1019.1020 = 1849 bp
4. 3466.3467 = 1751 bp
