# Supplementary material for: TurboID Identification of Evolutionarily Divergent Components of the Nuclear Pore Complex in the Malaria Model Plasmodium berghei
Source: mBio. 2022 Aug 30;13(5):e01815-22. doi: 10.1128/mbio.01815-22 (PMC9601220; doi:10.1128/mbio.01815-22)
Supplement: TABLE S1 [file mbio.01815-22-s0010.docx]

**Table S1.** Mutant *Plasmodium berghei* genotypes of this study

| Genotype | Plasmid | Gene ID |
| --- | --- | --- |
|  |  |  |
| *2.34* | n/a | n/a |
|  |  |  |
| *nup138::bira-myc* | pLIS0814 | PBANKA_0417900 |
| *nup138::bioid2-ha* | pLIS0574 | PBANKA_0417900 |
| *nup138::linker-bioid2-ha* | pLIS0653 | PBANKA_0417900 |
| *nup138::minturboid-ha* | pLIS0736 | PBANKA_0417900 |
| *nup138::turboid-ha* | pLIS0654 | PBANKA_0417900 |
| *turboid-ha ^nup138.PP^* | pLIS0709 | PBANKA_0417900 |
|  |  |  |
| *nup205::turboid-ha* | pLIS0705 | PBANKA_1140100 |
| *nup221::turboid-ha* | pLIS0710 | PBANKA_0416300 |
| *nup313::turboid-ha* | pLIS0706 | PBANKA_1310200 |
| *nup637::turboid-ha* | pLIS0760 | PBANKA_0107600 |
| *sec13::turboid-ha ^sec13.PP^* | pLIS0763 | PBANKA_1445400 |
|  |  |  |
| *rna pol II::gfp* | pLIS0010 | PBANKA_0807000 |
| *nup176::gfp* | pLIS0792 | PBANKA_1365100 |
| *nup269::gfp* | pLIS0778 | PBANKA_1454600 |
| *nup335::gfp* | pLIS0777 | PBANKA_0807900 |
| *nup390::gfp* | pLIS0642 | PBANKA_0309200 |
| *nup434::turboid-ha* | pLIS0720 | PBANKA_0309400 |
| *pbanka_0609700::gfp* | pLIS0782 | PBANKA_0609700 |
