# Supplementary figures and images for: TurboID Identification of Evolutionarily Divergent Components of the Nuclear Pore Complex in the Malaria Model Plasmodium berghei
Source: mBio. 2022 Aug 30;13(5):e01815-22. doi: 10.1128/mbio.01815-22 (PMC9601220; doi:10.1128/mbio.01815-22)

# Figure S2

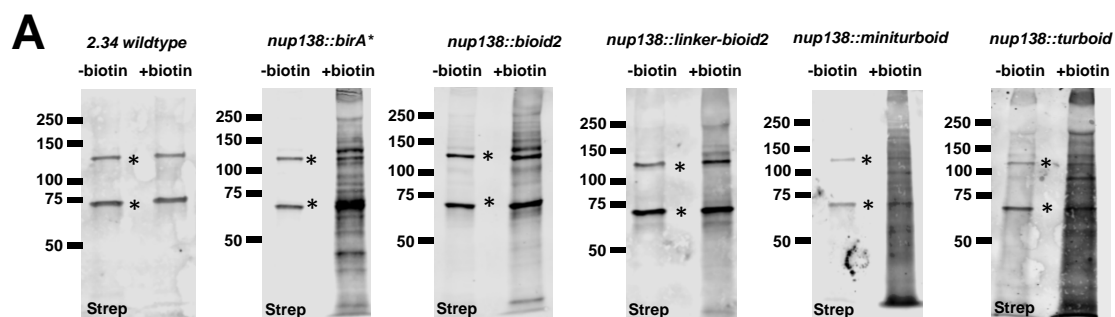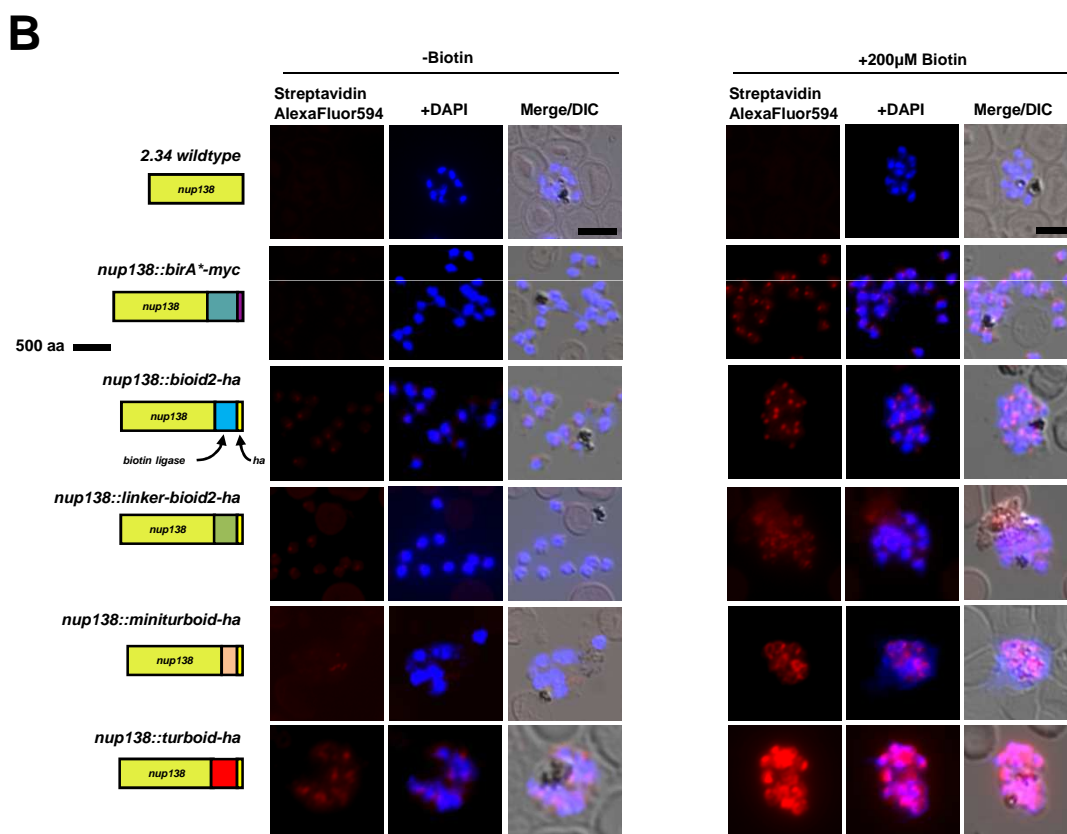

Supplement: FIG S2 [file mbio.01815-22-s0005.pdf]

# Figure S3

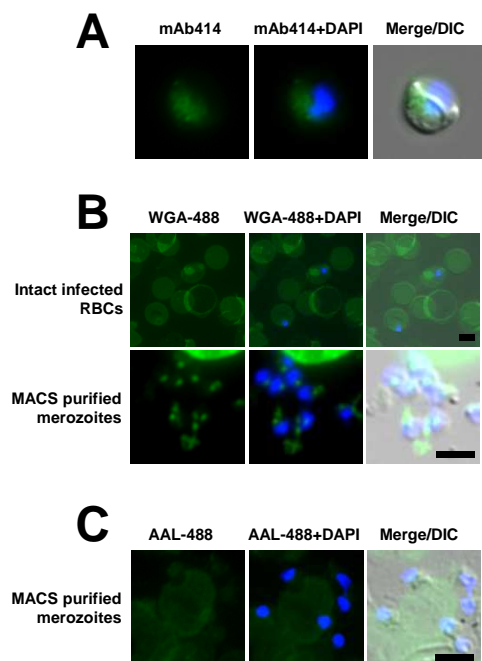

Supplement: FIG S3 [file mbio.01815-22-s0006.pdf]

# Figure S4

**A**

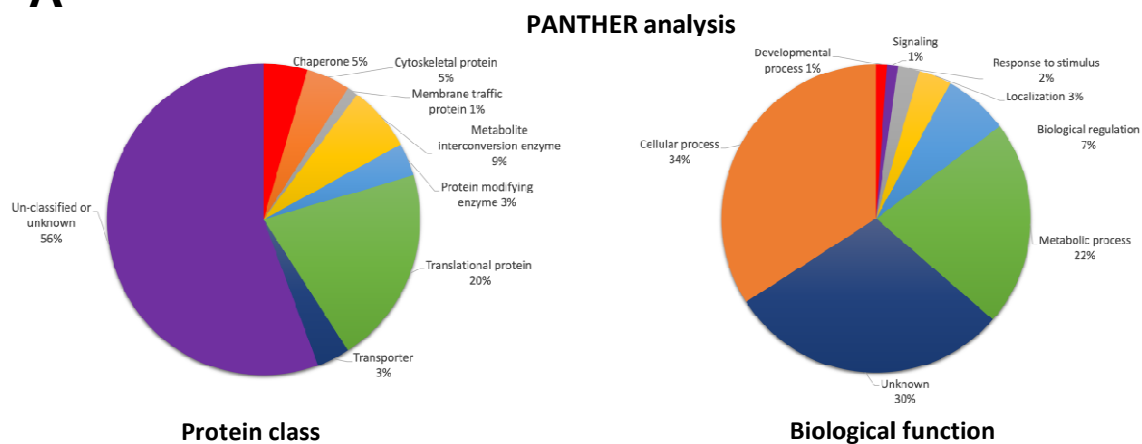

**B**

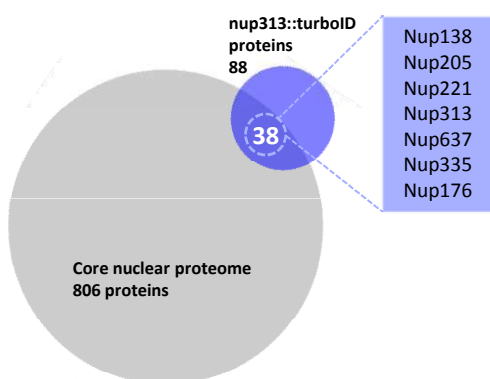

Supplement: FIG S4 [file mbio.01815-22-s0007.pdf]

# Figure S6

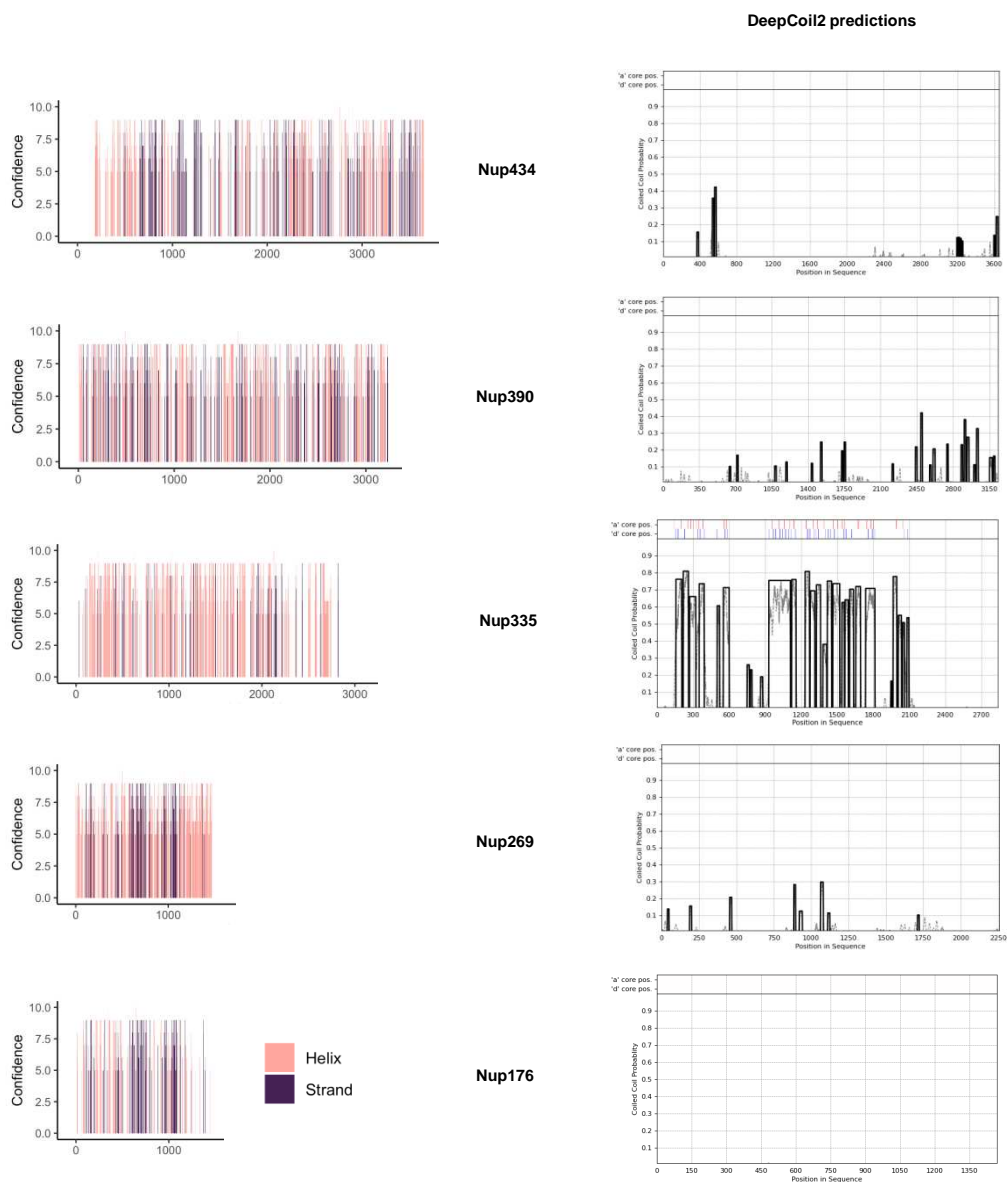

Supplement: FIG S6 [file mbio.01815-22-s0009.pdf]
