# Supplementary material for: TurboID Identification of Evolutionarily Divergent Components of the Nuclear Pore Complex in the Malaria Model Plasmodium berghei
Source: mBio. 2022 Aug 30;13(5):e01815-22. doi: 10.1128/mbio.01815-22 (PMC9601220; doi:10.1128/mbio.01815-22)
Supplement: FIG S5 [file mbio.01815-22-s0008.pdf]

Figure S5

A

Plasmodium berghei Nup176

P. falciparum syntenic ortholog PF3D7_1352400 conserved *Plasmodium* protein, unknown function

```
PF3D7_1352400      1 MNDFSEEYDFMQRIHKVEGYN-------ECNINNGFEGTKNDDLLFGKDKCEKEFYLKSK
PBANKA_1365100     1 MNDFSGEYDFMQRIHKIEGHNNNDYGINKDNLKGGDIIKGKDDVLLEKEKYEKDFYLKSK

PF3D7_1352400     54 YDKYSNVLNGFGKDDKYLYLKKLKNEKEHRKRTEGSSLGRSAVDYDGKKSNINELTKIHE
PBANKA_1365100    61 YDKYNNTLNGFEKDDKFSYLKRLKKENELKKRESSS-----------KKSNINELVKINE

PF3D7_1352400    114 IVFYIHANKRNVYDYLNKQTDLENNYYQIEEKPQNLTNDIVKENEEYIYLNENAYYKLKI
PBANKA_1365100   110 IVYYIHANKKNVYEYITNQTDLENNYYQIEERPNNLSRNIIKNNEEYIYLNDNAYYKLKI

PF3D7_1352400    174 NKEDKDDTGGGGGGGSRGGGGGFINGVTHNMGNMKIRYAVDENGNNIMGDIIEGDNFLL
PBANKA_1365100   170 NRDEKD---------------GREEKEDEHGIESMNLKYSLDGNKSTIIENIIESDNILI

PF3D7_1352400    234 NIANSLFTFKDYIKNVIYFLSKIFHYNFLDYTYIKNYSLTNYFDKNVKDYMKYLKKSEDT
PBANKA_1365100   215 HILHVLLSFKNCVKGIFLFLSKIFHFNFLDYNNIKNYSLTNYFDQNVKEYMKYLKKHEDS

PF3D7_1352400    294 RFLSQEFILRKKFIMPHFIYNCVDCNYNTNDNGIIILSSIYPFIHSYDYNQKLKDLRNQE
PBANKA_1365100   275 RIINHDFLLRRKYITPHFIYNSVDCNYNTNDNGIVILSSILPFIHSFDYDYKLKETRNRE

PF3D7_1352400    354 KELDNILNNERKRRR--KTFFSFVNKFRNFVVNKIYSDKNNVNNMTSRIRN---------
PBANKA_1365100   335 QDLEYVLNGEKKKYFGKTGIFEYLKKIKNMFINKLNRSIGNDNNNNNENGDGMGNDEGAW

PF3D7_1352400    403 ---QSEYSEFLKKFESSINEEGELIQDIIFPGKAQMIFINFKQLENNFTNMISNVDVKNE
PBANKA_1365100   395 GNDNNEYSEFLKKYENCINEKGELIQDILFAGKAQMIFINFKQLENNFNNIIGNIEIKNE

PF3D7_1352400    460 RVQVSNLILGYEKKEHIKYTDILISNILKNIKKKYPDMVINDENPVNFLLEQKDAIPKKL
PBANKA_1365100   455 KIHVSNLILGYEKREHIKYTDILISNILKNIKKKYPDIAINDENPKQFIYDDNEFSDKLM

PF3D7_1352400    520 SAESGNDFKNNININLNTNGINNNNNNNNNNNNNNNNNNNYYGDNNSYHNNSVNYNMHNF
PBANKA_1365100   515 GE--------------NNNTYGGNHINNMNSNDESNISLTEHIDNN--------------

PF3D7_1352400    580 IPSTNTSSYTNKNFLPSEMSSDVYFNKKDLYKLSDLKDYICNSPSHLMKEKKSSCEYVTC
PBANKA_1365100   547 ------------------------IYKRELYKLSDLKEYVYSAPSHLMRDQKNNCEYVTC

PF3D7_1352400    640 VCFDNNSNNCNLINNFVLGYNTGKLEYIYGKIIYSNISHCDLLIDYYSKKNKKFSYELNK
PBANKA_1365100   583 VSFDNNSNNCNLINNFVLGYNTGKIEYIYGKIVYNNISHCDLLIDYYSNKNKKFSYELCK

PF3D7_1352400    700 RIFLNGSVIKISFAPNGFFCLILTSRTLYLCNFFYFSIYEITNNCYNSQYVNFLWLNNNS
PBANKA_1365100   643 RIYLNGSVIKIGFAPNVFFCLILTSKTLYICNFFYFSIYEIPNVCYNSRFVNFLWINNNS

PF3D7_1352400    760 YVIFNDKGHIHLYEKNLKSDGFQGFSVIKTFFINQEIFFNSICEYHLYSNSIYLYTGEIE
PBANKA_1365100   703 YCIFNDNGYIYLYEKNSKSDGLNSFSLVKCFFIDQNIYYNSICEYHLYSHSIYLYTGEIE

PF3D7_1352400    820 KRKRKKSSGHLFKKKNHINNNNKNTKKKRTSEYFYKYDCNLIIIDLNSDVHKNISNYKGK
PBANKA_1365100   763 KRRQKKNVTHFFKKK-------KIRMHKRYSDYFYKYDCNFIIIDLNSYMGNNCENNKSS

PF3D7_1352400    880 EFLN-MYLKDNMNDIDNNMKEIKMLEKLIDNEKTFEDEDIPFDKLTYINIKDFNNYISTI
PBANKA_1365100   816 DMLVRGLLKDNVTDVEGNMKQIKILEMLIENGANLEEEDANFENMLNINIKDFNNYISTI

PF3D7_1352400    939 KTLGNNLYMNNESGDEHMLEYDPTKADENFSVKKLFVNYIKDVKRYLYNLKNDNIVNILL
PBANKA_1365100   876 KDANTKLYMND-SGDERKDMIDYNKENDKFSIKNIFVNYIQDVKRHLYNLKNENIVNVLL

PF3D7_1352400    999 PRYYSSTDDYNYNNFLNIRFFTIEKSDNKHLIALDTETDFVYIYKIRKNVYLDDDTLDNL
PBANKA_1365100   935 PRYYSSGDDYSHKNFLNVRFFTIEKLDNKHLIALDTETDYVYIYKIRKNVHLDYNSIEAL

PF3D7_1352400   1059 KNRRLVGGIGSGFHFNNPLQMFYSTYMKKESLLKDEWRIYNDYIYINTKKPQKFIKYNLL
PBANKA_1365100   995 KNNPYQTNR-------NPLHMLYSTYMKKEPILKDEWKIYNEYIYVNTKKLKKYIKYNLL

PF3D7_1352400   1119 YIIKNQRKIFFPLHVYVINTKQNENEYFLFIKWATIKNSFIYSSYSSLNKVSKMKEYFKR
PBANKA_1365100  1048 YIIKNHKKIFFPLHVYVINTKYNENEYLLFIKWATIKNSFVYSSYSSLNKVSKMNDYFNN

PF3D7_1352400   1179 DDEKLLKVLENPKNIFLYNINENDYPTSYLYNNNNNNNITSNRNNINNNRLLSRRNKLID
PBANKA_1365100  1108 EDEKILKVLENPKNIFLYNINDENRNVGGRMG-------YPYMPNNDILRSLSGKNKYYD
```

```
PF3D7_1352400  1239 KDYLSENDESYLLGNKYKDKLNKQHDPYRHSYR---QYLGPEDYINKTPMKNIPRNFFTS
PBANKA_1365100 1161 NDYMSDNGNNYISYNRSGNKPNEYRENFSNKMKNFTNRISHEDFMSKTPIKNPERNFFTS

PF3D7_1352400  1296 PVSPNNLNFNNMNMMNNNTYNIDPSRYSDNDVYHNNTAFYMNNKAHPTYQNYLQEKKNNE
PBANKA_1365100 1221 GVG------NNMGINETNPILYKRRGRFTENDIYNEYYMNQNNKDGNYSNYLNENKKLNE

PF3D7_1352400  1356 ADSKWSYFKKMNIFRKDNNNDMDNFHDADEDYYNRQRAMYNSDKDPRYISNKNRNNLNID
PBANKA_1365100 1275 SDKKWSYLKKINIFSKKDEDLINDNYGNHRSNYN--------NMDPKNLQYKNSHMYNLK

PF3D7_1352400  1416 QDHIINDMRSPHFYSTSNHMRYNRLQDGTDGSSTIENNDINNMNNMDFVNDMNHINNYPK
PBANKA_1365100 1327 NDNIFNEMKNSNLYSSPNDIKFNRLHDNQDGPKIPNGD--NNMNEYYYMNNTNKYKTSNI

PF3D7_1352400  1476 NNYDNNYLKYDSYKNIRLDTNNTDNNNNNNNNNNNNNDKDKDYNTNIAYNNKNFMNNDMY
PBANKA_1365100 1385 NNNYDSYVLKNDLYNFRNNININENNNNANNNYSYGNTDFYRAPNDTYYRGNQEFNANVY

PF3D7_1352400  1536 QISSDHNSYLNDIERTNNNIGYIYKNNKDFNNVDHMYNEKNKYKTDNQKDKESSYRKYI
PBANKA_1365100 1445 NAGYGS----------------------DSNHIHHESPYRSRFRNYS------------
```

B

Plasmodium berghei Nup269

P. falciparum syntenic ortholog PF3D7_1241200 conserved *Plasmodium* protein, unknown function

```
PF3D7_1241200     1 MEKQKLLNILQRELKEYGDEEMLSCFHRFYESIEANENDLSKKLFNELIQQMKKLFIIYH
PBANKA_1454600    1 MEKGKLVGILERELKEYGDKDILMLFHQFYQSIEESDYENAQDSFNEMVRQMKKIYVVYD

PF3D7_1241200    61 KRNKRDLIEKIKNFFKEESNEVNIIDVENNDKDNDSNFNYEDDYFMNNEDININDDNNKN
PBANKA_1454600   61 KRNKNDLIEKIKNYFKDETTEVNIVDISQEKLEVQLNKTIEEYDNFSKNLDDIKNGKLKE

PF3D7_1241200   121 KVRRKGKRSKDPKDMKLPHFKNHYSFLSTNLFDNINLHDLKSNVYEYDIFKLYNTHVEMI
PBANKA_1454600  121 NVEKN----------EFPCFKTPSSFLNTNLFDDINLDDLEKCEFSFDILDLYDKSYIES

PF3D7_1241200   181 LLEDNKDITIDDEPYFYETFEYFFNVLCLLYFLKNIYFDLNYKTNENSYSFLFKGFEERK
PBANKA_1454600  171 VDEMPDTVINDED--FYETFEYFINLLCLLYFLKNIYFDLSYRSNENSYSFLFKGFEERK

PF3D7_1241200   241 NKVLSYFFILKKLKIHKLMDNEVIKIFCNFFQYIYINEYLIRNIFDVSMNSFIYTAVKNF
PBANKA_1454600  229 NKIISYFFIFKRLKMYKLMDNEVIKIFCNFFQYTYINDYLIRNIFDNSTSMFIFTAVNNF

PF3D7_1241200   301 QKKYKMDNILVPLQSLTDADYSEQQQKEKVHNDMEKIICDNTTCVNNTSSKNSISDYNIC
PBANKA_1454600  289 NNKYEQD-----------------------------------------------------

PF3D7_1241200   361 VNKSTNELIASGNNNNNKTLGKSEISGNEIICQENITNNHKINKMNNTIITTTTTTINNN
PBANKA_1454600  296 ------NISLSPNKTNVNAQFGNNDKVGN------NISINQLFFGNNNIRYDIEKRERQT

PF3D7_1241200   421 SNNYNNNNNKVNRNLFSNNYFVKNSPNHEDFLNKELNNFFISHNQ--NKNNKHSFIDIIY
PBANKA_1454600  344 NGGYNDNNMGEKDLLKKN-----ENDNQEDYLNKKLNNFFISNQTPNNKSDKNSFVEIIY

PF3D7_1241200   479 NDINYDNIYTDEIINSLFYFFNNPYLIFTFHKNVLYYIEKKSRDSNFFHSYKNAIALIKY
PBANKA_1454600  399 NDEAIDNMYSDKIINSLFYFFNNPYLIFTFQKNVLYYIEKKNKDNEFFSSHKNSQSLIRY

PF3D7_1241200   539 LNYCVTTISVIFHIFVSAGLFFNKHIMIIESNKYLNFYKLLNFDNLLVDSDNYDQKK---
PBANKA_1454600  459 LNHCVITISEILHIFITSGLFFNNNIMQQKTNKYLNFYKLLNFNNMNFDYDIINENKSKN

PF3D7_1241200   596 -KNKYNPSEYNNKRNNNTYNNLSKRRSSTHMNSSPRTHEKDENIRKNTQKKKVESFDSLN
PBANKA_1454600  519 IQDRANDSNISNTKKNTNLVNKHKTVSKSHGKGGEETKKNSKHYHNHHERRKRAISDDTI

PF3D7_1241200   655 DSSSYNDDSINENELVVHNNPFDKKNREK---------KNYGNLEDNKFYDFNIIKYLMK
PBANKA_1454600  579 VSDIDNKYHYSDNNKLFFGKSNKIINLDKSKYNYANTTENKKDIDDINMQDFNITKYLNK

PF3D7_1241200   706 YKNVVSNLNQDHFNKKDYSNESEGEVNDFVKLDQSNINSRGSYTSSHYNKGDVTNSSYDK
PBANKA_1454600  639 YKTVLRKLCENDFNKNDTTSNDDEEQINTEDYYDEKRSDKNLKNINNKHDNAYLRNNYNR

PF3D7_1241200   766 SNSYSDDDYNDSDTSKFQENRYKHSYKNVKYNKSSDDYENDSNSSSNNNNNNNTYSDDDF
PBANKA_1454600  699 NSMSYNTDTYDSDVERESGSHSSGSKEEYYNSKHQNEDNKPRGYNNNNSDSNSMRDNNSR

PF3D7_1241200   826 NSYNRNRRNTQRKSKQMKNERFQKKNKKKNKIDQEDSTMSDEYNMNNDLYSNEYTNRSIF
PBANKA_1454600  759 YESDDSEGSYQNTLYRGKDKNSKKEKKNSKKSNIDNNSTTIVHITNSMYKTDDISNKKIF

PF3D7_1241200   886 NDILHKAKKRKRFYDLISNINRMKNTFNVNNINKNKTIDFNYIENIDTISYFISIYNSEH
PBANKA_1454600  819 NEILNKSKKKKKFYEIIRNINKLKNAFDVSNIQKNKNIDLNYVENIDTISYFISIFNSDN

PF3D7_1241200   946 IFQFDYYFLKFIAEICTTYNNYIKSYLININNNNNNNNNNLKYRREDLDKIINQVEHFLN
PBANKA_1454600  879 IFQFDYYFLKFIAEITTSYNNYIKKHIVDDNS--------VKIYGNDIDKLANEIKQYLN

PF3D7_1241200  1006 EVTKLIYVYTWCILYIFGHSYSYVKYAKYFYQKKSFFLLDKKPHFIFNQFSYVTQESFDN
PBANKA_1454600  931 EVTKLIYVYTWCILYIFGHSYSYVKYAKYFYQKKSFFILENKKQCIFNNFNYCIQENEYK

PF3D7_1241200  1066 ISHQNSNSNSNINEKDDIYKIKKNNNNINNEDHFDYRKHLYNNYNDDIPINHFFNPSNIV
PBANKA_1454600  991 NGSGEGYTQETDQQSD---------------------AFYLQEAEDDVHLKNFFNPLNVV

PF3D7_1241200  1126 ENLKYNYELKRNTSEFDNFNFNNLIHIYKNSYFQNIVDKLQLNLKFIDNILFTLDNILKN
PBANKA_1454600 1030 ENLKYNYEIRRNLNEFNNFNFHNFINIYKNSYFQNIVDKLQLNIKFIENILFTIDNVLDN

PF3D7_1241200  1186 KKSK---KKKFLNYLCVKDQKLDDICNSIDNLKRNINKYRQTFHYCLNFNNIKNVIMLTL
PBANKA_1454600 1090 KVSKSKKKKKYINYLCVKNQKLDDIYNSIENLKKNINKYKKTFHYCLNFNNIKNAIILSL

PF3D7_1241200  1243 EEMSMSSDKLELNKLLNEYLNKLESKGQLKKRQKKINIKIIDNNIILDTNNIKKEREREK
PBANKA_1454600 1150 EEMNMTSDKLELNKLLNEQLNKIENRGQLKRRKKKINVKLIDNNFVLDTHSKKKRKKMHR

PF3D7_1241200  1303 RKSVENFLNKKKFLKSYMDMNDISNHLIDKRRNKSIKMGTTNTNHRRSSSVANRNRSRSI
PBANKA_1454600 1210 -------------------------HKLSDYSSYADKTMEYDISSSYSEDNSNNSVEKNK

PF3D7_1241200  1363 LSSSFLEKKEKSKKYSIKSQKKYSEHVNTDRSYIKNNSHINNKLSICMDNNKKKNDGINK
PBANKA_1454600 1245 MIQYTFGAKTKKERRKKESNRSYSKHNDTSSLLQNEHSRKNLDNKNSFEMHNHESSGS--
```

```
PF3D7_1241200  1423 SFMINDNTNRGSINNDSYYRKNNRKYEDSVNYKNDMLERSRRRSHNYYSSSEDNKSDNYN
PBANKA_1454600 1303 ---NYKNSKKGKINHDSKEFNKNRKGKHDYLGEIEDINKMYESSGGSNKYDGNSDTLSID

PF3D7_1241200  1483 INDNNDYHHNDTDTDEDNEYYSDNYKKGGNQNGNKLNDNNNNNKKNKNEKNKMSELIFN
PBANKA_1454600 1360 NSLSSNISSGDSNNRNYHNKQTNKKKTLNGRYNNNIYNGNVNDKNNINNIEEKENNIILN

PF3D7_1241200  1543 SEYHTERLIFFFNLLNGNNLFYYLSLHYLVKNEKIMEILHFLPLKYLLDLLILYDLKDYI
PBANKA_1454600 1420 SEYNTERLIFFFNLFNGNELFYYLSLYYLIKDENIMEILHLLPLKYLLDLLILYDLTDYI

PF3D7_1241200  1603 KFKTIIRVFRIIYEFQDFSPILREDIFFNKNNYCLENADFMNYEFWCTFLTVNKKDLS--
PBANKA_1454600 1480 KFKVIARVFRIIYEFQDFSPILSESIFFNKNYFFLNNADFTNYEFLCKFLTINNLDIYKI

PF3D7_1241200  1661 PKKAYDDDNYNSIFMALKQTAIIYNNMHDKKEDNFEDIRKRDDYSNAADDDDHEHHHDHS
PBANKA_1454600 1540 EKKTIDESNYKAISTNNIKSNDPIDNGVFIENENEIDITADENINDAYKNE---------

PF3D7_1241200  1721 NNSNYYNDEKYVHMDNNSHDVKKHGFFVDLIKKVFSLFNFSNTLKSYPVLLKELLQKLLI
PBANKA_1454600 1591 -------------------KRPLKEVFLNLIKNMFSLCNFFTNIKSYPIVLKNLLNKILI

PF3D7_1241200  1781 NIIGKHSCINDNKHYKGYNSIKYYALKFVYNNLKNLTFECNTSSYYSYTINNQTELYNIS
PBANKA_1454600 1632 SIIGKYSYINNDRNNKDYNSITYYILRFIFKNFKNITLGYSNSSYFNYTITDPTELYNIS

PF3D7_1241200  1841 IHNFILYHIYELGMRTKELIIKSSCFKLIKYLLTEYYYYPDIFNEKCHELINSDIKINKF
PBANKA_1454600 1692 MHNFILYHIYELAIKTKVLSIKSSCFKILKYLLTEYYYYPDLLNEKSHELINSEIKINNF

PF3D7_1241200  1901 YIKNVILDFVSFNIMICHLQRELTSDLNVLNNLDLFNYSIKSIANYLIGYKNNSFFFLID
PBANKA_1454600 1752 YIKNVILDFVSFNIMVCHLQKELTSNNSVLNNLDLFNYSIKSIANYLIEYNDKSFYVLMD

PF3D7_1241200  1961 KKMVKTLLIKLTFMMPFIYKLKIPACVIRLYIRVIKSLDKEILDTIHNSNKIDLIYDSLF
PBANKA_1454600 1812 KNVLKTFLIKLTFMMPFIYKLKIPACVIRLYIRVIKSLDKDILDNIHNSNKINLIYDSLF

PF3D7_1241200  2021 YIIISHFIYFFLNMNIELDEEASNILSCNYNKGAFPNKFEKNDNYIKITNDTSEEKESGN
PBANKA_1454600 1872 YIIVSRFIYFFLNINIELDEEATNILSSNQ-KNQFSRNSFDNTSYIKINTVENEESENYN

PF3D7_1241200  2081 VQE-----------------------------------------------DEEQLSCRNI
PBANKA_1454600 1931 EEERGNFRDKYNKNTQSVKQTYDYLTVDHNYNNYSQNEELDRIIEDIKSSNKHEVSEEAV

PF3D7_1241200  2094 NSFEKSIDDEGDKNN--------------------------KNKAKGKFLCIDDEFNFEN
PBANKA_1454600 1991 NSDEKVMNRDEEDNNSDEKVMNRDEEDNNSDEKVMNRDEEDNNSDEKVMNRDEEDNNSDE
PF3D7_1241200  2128 NVQNEMDQNNERDEPTVERKSDDSRND----------------------------KDDNS
PBANKA_1454600 2051 KVMNRDEENNNSDEKVMNRDEEDNNSDEKVMNVGDGEANGENEMKNDTLIDDMNGRHSDD

PF3D7_1241200  2160 HSDYSNDTNNSNDTNNSNNSNDTNNSNNSNDSNNSNNSNNSNNSNNSNNSNNSNNSNNSN
PBANKA_1454600 2111 TSKRDIRTESKGDSDNENKRNSLSDDTSYSDNENKSGSLSSDKSYSDNESKSDSDGSRSE

PF3D7_1241200  2220 NSNSNSPNDDLSKNQLNDDISAKNFNKLSDVLYIRKIINENSNFNLENNYFINYSLYILH
PBANKA_1454600 2171 YDRSSDESPLQMDNGVNANVNTNNVNKISDVLFLRKVINENNNLNLDNNYFINYSLHILH

PF3D7_1241200  2280 KEHYSIYVFFSTFFKNALKEYNDICKSQKV
PBANKA_1454600 2231 KENYSIYVFFSTFFKNALKEYCEISKKHKI
```

C

Plasmodium berghei Nup335

P. falciparum syntenic ortholog PF3D7_0317300 conserved *Plasmodium* protein, unknown function

```
PF3D7_0317300     1 MNNDFMKNEKDDYDKVNEEEYSLENFRGIDMNNNVDISNMNNNLNDVGELINIDNNDKYD
PBANKA_0807900    1 -MNNNMQTSDKNVMENDKLNFKSEASKLLDFKTTGNYGVLGLGKDANFSIANTNIRDGDI

PF3D7_0317300    61 EDQLSLIKESLEYGELLLSNVKEEDENNRREHFKKNDYMKDTEDVHYK-ESKIGQDKRFM
PBANKA_0807900   60 QMDLDFLDMEKELDLLNDENYKKILEGKNNNINNGSNINGTAIDIGNCSISNFPGDDNMI

PF3D7_0317300   120 HSLCVKELKSTEEGELFDDEKYKDKDTLINMLRKKLEIKIKDYNLIMDTLILTKEECSKK
PBANKA_0807900  120 ESLCINELKSEEDDHLFSDKNYADKDELISMLRKKLKFKIKDYNLIMDTLIRTKEECSKK

PF3D7_0317300   180 EEQLKDYKMKYNKMEKECYSLKNEIERKNNEKHLNIGSFSFYKNEYDDMKYKLLKCEEKN
PBANKA_0807900  180 TDQIKELQTNNNKIEKECFELKKELERKNSQSNLNIENHSFYKKEYDEIKYKLAIYEDKY

PF3D7_0317300   240 KILLNKNQELHQTIIQMKNDSYNQNIKFKKDIDVLTEDKKNILIQNKNFTKQNKILIDKY
PBANKA_0807900  240 KEVLKKNELLNKEITSLKNEKIKYEINKKTDIEKFKLEEERLRDENEKLTSKGNLLINKY

PF3D7_0317300   300 LRQEKIIYKLKKHNEEQEIIIKECHKKIEFVNKNSVNHLNKVRDVLNKSLIKSEEHVKLY
PBANKA_0807900  300 LEAEKKTYAIEQKYKEEIQNLKNILNKEEINKKNSIQNFAKVKDILSQSLAKSEESYKQV

PF3D7_0317300   360 TNLKKENDSLKIEYNKSKTNIQQLNEQLVNYKNFIKEMEKKYKQLVVKNNSLFSITHDFI
PBANKA_0807900  360 IQAEKERDNLKVQNENLQNTINDLKGKIDNLKNCMKIFENKYKEIIKKNNNMFAITHEFI

PF3D7_0317300   420 NLKNSNIIIIRRTSDMKQIFKMYNLDIEHFNEQDHLSVIYIYEILYNTNDNNNNDNDNNN
PBANKA_0807900  420 NQKNCKIIILRKNAEIKDIFEKYKIDKNIFQSINDFSALYIYEILIQNSSKIDETEMTEF

PF3D7_0317300   480 DNNNNNNNNNDNNNNNNNDNNNNNNNYNNIMMMIENMNSGNHPNSNNLHNYRHNTNDENN
PBANKA_0807900  480 ESGTYDISENDDK-----------------------------------------------

PF3D7_0317300   540 LSSLKTSFRYKINNKSGKSRSIKSRSHFSGDNEYIQDQLSNREMKDHYNYIERYNHSSNR
PBANKA_0807900  493 ------------------------------------------------------------

PF3D7_0317300   600 NDIHNLYYMEENVSHNNNKSDYDEDGDAENNNYYIKIKKNKYKKLNDLLNKAQMKIITLT
PBANKA_0807900  493 --------------------------------YIKIKKKKYITLNYMLNEAQIKIITLT

PF3D7_0317300   660 RANEMFEKYCSNIKNTLIRDDMKKFRKPDIS-DVHILHNEKIYLEKLLNEKLNYIKDIEK
PBANKA_0807900  520 KTKEHYEKLYSSSKNNSHKDDESIYKKHYLSSDMTNLYNEKVHFENKLVEKIEYIKNLEN

PF3D7_0317300   719 KLDELHGVINKNKEDIYILQVEKQTLIKVISSVYDYTKMESENHIFKMNTTWNKMLNNVH
PBANKA_0807900  580 KLKEKESVLEKSVETNMRLRLEKDTLIKVISATPEFISIAHQSNGNYSIQSFVEHTVIEY

PF3D7_0317300   779 MSSNKDYNNQNNQNIENNQNIENNQNNQNIENNQNIENNQNNQNNQNNQNNQNNQNNQNN
PBANKA_0807900  640 VQKHERQKRDNNNMITSAVIDDYNQQTPSCISNNSGGIKLSHPEEYNQRNIMSNLVINN-

PF3D7_0317300   839 QNNAGIHISPDDFLKYRMSLEKFIQFTIKEHIVYIMKDEKKKTSNIIKESISLKKKHTKK
PBANKA_0807900  699 ---------------------------------------------------THERKMKKK

PF3D7_0317300   899 SIINNNNDNNNEDDDDNDMLSVMYSNDDVIKNKRKENNKEILEEHVSFSSFSNNEYIAHS
PBANKA_0807900  708 DFSNTTYNGNEECHTHSNVDNTNYSN--------KEDGSYIDDYYYSPSSYAIEHIMAN-

PF3D7_0317300   959 FNSILLQLSNYIFNIECKQMEYFKNSNLLSYVDDYTITIELFYRLKKYNNIFSIEQILGT
PBANKA_0807900  759 -------LEKAIVEIENTQIAYFRSSNLLTYIDDYMLTLQIFNRISDYNDAYKVENLLEN

PF3D7_0317300  1019 QYPSILQKLHDGIYCLKDNNKKKNNDGDNKSQEDDDGNKKKNNDGDNKSQEDDDGNKKKN
PBANKA_0807900  812 EE---------------------------------------------------------Y

PF3D7_0317300  1079 NDGDNKSQEDDYGNKKKNNDDDDDDSYKIELIVDELNKCKKNYTDEELYELMKGSDFDIF
PBANKA_0807900  815 NFIIDKTCKNFNNNDNTFLGSYPNDDSKIGIIITELEKCKKDYSDEELLHLFE-EKKLL

PF3D7_0317300  1139 KKYKNFYLNHFININNIFSTIISFHIHNIEDKYKKVYERYFNLFNFNFSNVELSFDLLIR
PBANKA_0807900  873 EKYRKFYHDHFVNINNIFNKFISLNLFNVETKHKNVYERYFNLFNLNFSNVEISFDLLLK

PF3D7_0317300  1199 RFDKILRLTKKYEQLLEENYEKIKNKNEKEEYLHACIKELEMNLERYNNEKIILDEQINE
PBANKA_0807900  933 RFYKILRLSKTYENLIIEREKKIKDINNAQINLYNDKQELQTNIELIKKEKEYLEKLIIK

PF3D7_0317300  1259 KEKKINIINEKYLILEKEYEEYQNKNIFINAQIENLEKEKKQLQEEIIQKDMINVKLNEK
PBANKA_0807900  993 KENNVKEINEKYFQLEKEYNEYKNNNCIIMEQYENMKNENYNLEQELSKKELKNNTLNEK

PF3D7_0317300  1319 NCDIIKIYEKEKQYLHTLLQENKDSHNYLKDKFENLLNLNEKLKYDHDISLNKINTLWLE
PBANKA_0807900 1053 NCEIINLYEKEKACLQTLLHESKEVNLYLKEKMETLLNNNERMKYDYDIRLNNLNALWIE

PF3D7_0317300  1379 EKENNKKNTFHMNNLRVENNNLLLKMKELQNKYNIIKKELNERIKQINVFRNNVSTLSLR
PBANKA_0807900 1113 EKENNKKSNFNVNSLKKENNNLLIKIKEMQAKNQLLKVELSERIKQLNMFKTSINSQ---
```

```
PF3D7_0317300  1439 DNRSTRGSIHQINNMYMNNTHLGFMGASKINNNISNLYYSNMIHMSHRGSIIKNKEDAEG
PBANKA_0807900 1170 ------------------------------------------------------------

PF3D7_0317300  1499 NSTQARMNNKDSTDNIINNIHNTDNINNMNNINNNTLNSINSNHLYYPFPFHNNVNSPKM
PBANKA_0807900 1170 ------KNNKDTSSIVGYTLNSVNTTVNNNASIGDDLSGG--------------------

PF3D7_0317300  1559 VGMCDVTLASGVNKKDDFLLNLEENEENSFLEYEIRIKSLQEELCDKESEILKIKGEKNI
PBANKA_0807900 1204 IEKKHIAHGNYLDRDQNCNYFLNIDVTACKIEYEEKIKKLEENITDLNAEILKLKGEKEI

PF3D7_0317300  1619 LITCIETWKCFCKNSKEEISRLKEICKEQLEKHKEFLLINKSNEDKLKYINSLLCDEKDK
PBANKA_0807900 1264 LLISIETWRCFSADSKEEIKHLKKMCNEQIEKHKEFILINKTNEDKLKFVKNLLMCEKDK

PF3D7_0317300  1679 YDIVVKDIKNNMRNEIDKLNNDINEKSYEIKLLKHENNNLINEMNILKNKETENMNIKQK
PBANKA_0807900 1324 YEKDVDNIKEKLTNELEKVSDELKEKTLEIEKLKLTNEKLLLKAQIIDNGKNDEMNMKKK

PF3D7_0317300  1739 EEDYIKLIKKDKTNIQNEYNDLLEKYNEVVVKNNMLYNDMNVLLKEHKEEIFLLKENIKI
PBANKA_0807900 1384 EEEYIELLKKEKENVEKKFENTSEKYNEQISLNKKLTDDINLLVSTHKEQVKVLNEQINI

PF3D7_0317300  1799 LQKDNTYLNDMFKNQINYVDNNLLKNRLDQLFNINQDLQKHLDTNQKHLEQLKYDYIEIK
PBANKA_0807900 1444 LKKDNKYLLESLEKEVEVSDNVMIKNRLEQLVEINKDLHKEIQENYNNTEKMKFKIIELK

PF3D7_0317300  1859 ERLKIEKTKINKQEKYIIQLQKDNNLILNDFNSTTTTTNNNNNNDNNNDNNNDNNDNNND
PBANKA_0807900 1504 ENIRVQKETHLQQQKCIIELR-----------------------------AKLIDNNMTN

PF3D7_0317300  1919 TYQQFIHSLKANLENSRLELKELSNLNEKIQLSDEKNRMKITILEDKLFKNEKDKMKLQQ
PBANKA_0807900 1535 QKDEYVSNLKINLESSRMQLKDLCDQLEKGNLNEKKSSMKIQNLETKLIKEEKERKRYQM

PF3D7_0317300  1979 IIDDNNKNYMIQYNKLKTNLDMLSEENRMLLLNKEEYEKQIEQLNHDHKLFISTKNNDIQ
PBANKA_0807900 1595 ELANKNSTDLISYNKLKTQVEMITEENKILLLRKECYEKEIEQLKRDSQFFNSTKNNDMN

PF3D7_0317300  2039 IIENEKLQEQVDQYITTINEKDKIIVHLNLQIKKLANQNEHMRSRCDIFNVAHSQDNIKN
PBANKA_0807900 1655 IIERDVLKKQVEEHIAKINEKDKQIVNLNFEIKKLYNQLEEMKDSMNRIESTTS------

PF3D7_0317300  2099 DHMVVGEDIMGDTNHDVNKNIDQGTNQHINQGTNQHINQGTNQHDTCDGPNYNYVKVQNA
PBANKA_0807900 1709 ---------------IFDRPIDEGSSLNIDNSKTSQNNQ---------------------

PF3D7_0317300  2159 TNREDNKNKERNLSQEIYKYINENIDLTSELEKKNDMLENYKNELKEKNEEIYKLNNDID
PBANKA_0807900 1733 ------------VSLEIYKYINENIDLTAELENKNDIIEQMKEDVKNKNKEIAKLNKDVI

PF3D7_0317300  2219 MLSNNCKKLKESIMMMEKYKIIMNNNIQEKDEIIENLKNKYNN-----KLDDLINNYSVV
PBANKA_0807900 1781 NLSANYDKLKESIYMMEKHKTNLNEYIKQKDEIISLLQQKVNNNNSNNNNNNNNNNNNNN

PF3D7_0317300  2274 DKSIVSCFEDSNIMSPSCNDILNVFNNLSKSNKKVCTNMDICNENMDSISSINNVNNINN
PBANKA_0807900 1841 NNNNNNNNNNNNNPTKCCNLVENNNSNQNVGKEDDSNTPIKYNINMKDNNSKKIEEKIMG

PF3D7_0317300  2334 VNNINNVNNINNVNNINNVKN----IVDINNYLVNNLQLNKDNDNIIIIKFNILKLFKLG
PBANKA_0807900 1901 MEEIMNTSCEEVINTLKKIPRNSVESDNNNLSSSESREFSVSSDHITLIKCNILKMFKLG

PF3D7_0317300  2390 SCYLYIINRNLKEIQMLKNQILSLEESIKSLNEFINNLKNENEKNELIKINNFEEILKLK
PBANKA_0807900 1961 SCYLYIINRNLKEIKILKDKINYLEENIQSLNLFINNLKDQNKNNEVIKINNEEQIIQLK

PF3D7_0317300  2450 NNLQDNESCIQNLNNYLKKNEELNKINVKNIFKYKGYIIHLIQQSNVFCKIFKHFNENKI
PBANKA_0807900 2021 NSLQNNENCISNLNDNLKQKDEMNNSNIKNLIKYKSFIINLVHQSNIFFHIFKIMNTQKV

PF3D7_0317300  2510 IDQSIINKLLYLKKSFDFYMYDSVIQEIRENK----------NIIINQDFLTDEYFKHIQ
PBANKA_0807900 2081 IQNSIYNQLTQLRKELDFYLNDYIMISELENKEKLNLSNIENNLLNIVSTFSYENYEHIQ

PF3D7_0317300  2560 TFTKTCNVLIQRGYLSILKDTNNDFFIQNKQSNQQGNQN------GNHINMCNIYPDDEI
PBANKA_0807900 2141 IFTNKFNFIIERGKVSIFSDGKIHPSQNDQTNNFFSNINSYFQNQNQYNNALNFKDLNEN

PF3D7_0317300  2614 NVTADQQIFDGTENVQQSLQNEEDYVNNEEMYTDKMDLDNNMNGDDDDDDDDDDDDDNNN
PBANKA_0807900 2201 TIETPKDTQVQTVQIYQETQNEEENIKNENIATQVNNQNDQIDALKNEEYNDDQMYSVEN

PF3D7_0317300  2674 NNNNNNNNNNNNMGDEDNHLVNAFNNHNLLTNGNVKSDQINNETLERYEENIIQNIYTND
PBANKA_0807900 2261 DGTEEEDYRAN--------------------KEHTEHDEHEEKCKEEGDMEINENVLNNN

PF3D7_0317300  2734 NVDNNQVIENINKILIKDKQDIINNDELKNEHNNLIRLINESIENAHNLENVYVQNDANN
PBANKA_0807900 2301 KEKENTNIESYNEYNNNREEYADVIDNDEEKEGEKFSYSNKERSNID----------NNN

PF3D7_0317300  2794 LINDNIKKEETLTYVDEKDNVSNESNSKCDDDKKENEDIIQAKNENYPVSTHYDNNDDIN
PBANKA_0807900 2351 YDGNNCDGNETLQRYEENVIVENGNN----------------FGEKEFLTSNVESNLFEN

PF3D7_0317300  2854 KDNINNDNNNDNINDDNNNDNINNDNNNDNINNDNINNDNINNDNNNDNNNDNSNNGFVC
PBANKA_0807900 2395 NYKNNDDNGNNIVSEVSEENKLLGDDSVKHINYENITIGININTSNPQNEENISPPSFTN

PF3D7_0317300  2914 ELSSNINDFNNILNVNKDNFQGINKSNNFSTNLSEYNYDAYVKIVEAGSALENKKQQDKK
PBANKA_0807900 2455 KVEN---------------------HNNTFVNGATCSYLSECEFSSFDNNDSFKMKGSKK

PF3D7_0317300  2974 RKYFSDSEITKENGYLGESNSIKIRKVSVGGDNDNNDDDNNDDNDDDDNDDDNNDDDNNN
PBANKA_0807900 2494 RKYHSDSEIAKQN-----SNNKAEHAKKKMRKMNNSHKFTSGGTSSGIAENPTQQNINLD

PF3D7_0317300  3034 DDDNDDSYDDEEKEQENYSNDVNTELNNHVTFEDISVHNNIQENKKNYFSNVHENTFNLH
```

```
PBANKA_0807900 2549 GNDNDNDNLGGSSPNESFDNYYDEEKKKYELLENISKLN----------QAYNSNNLDAT

PF3D7_0317300  3094 VDENVHEDLQNYEDYVNNNNNNNDDDNEEESNNSCYIISSDDEGDKKNVSKQNDDDEDDD
PBANKA_0807900 2599 SDENNYTKKNENNSMMKSDKESQNENEDAKESDSCYVISSDD--DRQVMDYGQDEEEYEE

PF3D7_0317300  3154 DNGDDDDNEDDDDNEDDDNGDDDDNEDDDNGDDDDNEDDNYDDEDNDNYENEDDDNYANE
PBANKA_0807900 2657 EYEEEYEEEYEEEYEEENEENEEENEENEENEEENEENEENEEENEENEEENEENEEENE

PF3D7_0317300  3214 DDDNYENDDDDNYENDDDDNYENDDDNYENDDDDNYENGDDDNDNDHNDDNNDEEKYSCH
PBANKA_0807900 2717 ENEEENDGEVDETEEEDREMDEADYEQFDSKGELNSSICDEGDESNSDDADKDMIDYDEI

PF3D7_0317300  3274 DDKNEHTNNDLLNIDHDNNKNNITDELYSTYNVSVSHNKDPSNKENEIQNLISIDSSNEN
PBANKA_0807900 2777 DNMN---------------------------------------------------INEN

PF3D7_0317300  3334 DENDENDENDENDENDENDENDENDENDENDEKDENDENDENDENFDNNNEGTLNEMNSE
PBANKA_0807900 2785 RNGEDSEAENGKYDCNSNGSNCLDEKNKNKDPIISIATSDDENDGGNEDRNETFNE----

PF3D7_0317300  3394 E
PBANKA_0807900      -
```

D

Plasmodium berghei Nup390

P. falciparum syntenic ortholog PF3D7_0212400 conserved *Plasmodium* membrane protein, unknown function

```
PF3D7_0212400      1 MRKEYISNKKVYELLSQGTYEGYEEEIKTIFERRYMNILSYLCPPNKRSFEDEIISLKIQ
PBANKA_0309200     1 MKNQYLSNKKIYELINSKKYEGYENSIKEAFEFRYANLLNYLNKDNN---YYNIISIKLQ

PF3D7_0212400     61 KYINEYLSFSNSTSYFFGFSKELIFHCLKIYFTDIKYEEVDVMKSCKHVSMMDSEDTYNN
PBANKA_0309200    58 HFINENLNFGNLKSYFFGFSKELLVHSLKVYFYNEIANNDSFILELNSIKCDDKSVLNDN

PF3D7_0212400    121 NNNNNNDDDDKYNIESD----EELIERIKKNADALNMEKLHLLYCDEYFYIFKNILYVLK
PBANKA_0309200   118 QNVFDPGFIDKMNNEYDNKMDKELFKILEENIKFIDMEKLHLIYFNEYFYIFKNILHILK

PF3D7_0212400    177 RCEVFYFYTNYKVEEFSYEKNFLYDIFYIIEKKINIQDLIQIYYDTYKDTNNYFDLLKNM
PBANKA_0309200   178 SCYIFYAYTKYKIDDLEYEDNLIYQLFNIIEKKIKIQNFIQIYFDTYNDTNNYFELMKTT

PF3D7_0212400    237 DIENIVWIYKYILLFLNIQIYCINIFLYLQLRLNILSQSNIKSYLHIFLTFPKLSLSNTY
PBANKA_0309200   238 HTENYPWLYKYILLFLNIQIYCINIFFFLQLRLNILTKNNFKTLLNIFITP-STNISKKN
                            ***********************

PF3D7_0212400    297 MNEDIITSTLQQKEYSVIFFICSLCNIITYEDIHKYVNCCLSINLFKLFFFFKKNYHTND
PBANKA_0309200   297 NFNDIIIYTLKQKEYSIFFLLISVNNIIKLDDINKYIKSPFSNNLFKFFLIYQKNKNSP-

PF3D7_0212400    357 NRNYYEHNIKMLSGSLHYFLDILKNFIFINDNFVYKRILIQILENSLCTYSIQPVDFHNM
PBANKA_0309200   356 --NIYEQNIQTLSNFLPHFHDILTNFIFIKDNFVYKKILGDLLDGILVIDDNISVENDGI

PF3D7_0212400    417 LFDSMGFGTPHSKNENENENERLYRRNVEFNDADIYKKNGNIKNVNHGCDEHDGNVSYFQ
PBANKA_0309200   414 LGDQYIN--------NNNNNIFMIKNGVKINNKNYELNNRDNDNTNEYLFDRD-------

PF3D7_0212400    477 TPNTTNDYNKELQNEEYNLDVSNLNNMFEDEDRYKTNQDVNININISLVKNMKKHIEQKE
PBANKA_0309200   459 ------------------------------------------INKYLVKNMKKHIKKKE

PF3D7_0212400    537 FFKRNINKNLFLNVCILLFKKQNFLLYTHDIKKEYKNINTCLEYLQNDNYQYDIYSLKYF
PBANKA_0309200   476 FFKNNIDINLFLKICIYLFNGEDFILFVNDIKKCYKNIKTCLDYIKDDNIYQNAFSLKYF

PF3D7_0212400    597 LHNYDYKETEIITNFKEKEKSLCPFISVESKNILLEISSLFFSFDYLKAYTEENNIYGDN
PBANKA_0309200   536 FNLYNYKNTEIVTNNIEITKHVSPYIYIESKNILLETSTLFFAYDYLKAYADTSTNVSNI

PF3D7_0212400    657 NKKNFKINNIYNIIDKEKKKNEIKKKNNNEMKIERKKEKNNNEMKIEINKEKNFIDNTIL
PBANKA_0309200   596 N--------------------------------------------SQLNGEENENEKGMF

PF3D7_0212400    717 HPVILYNVNRLLLDFFYDKISKASIQTLIIFNHNLLSIYRILKTLIGTNTNLINFNEVTK
PBANKA_0309200   612 HPIILYNVNKLLLEFFANKICNFPIHTLIMFNHNLLSIYRILNVLIGTNANLINFEEVEN

PF3D7_0212400    777 IVEKLAQEEGNITKKNHKNIHMVILEDTLYYKIIYILRKKNINKDTIYITYIEYYYILLD
PBANKA_0309200   672 NLESLSKLS------NNTDSIITILEDTLYFKITNILKTKNLDIKNIYITYIEYYYILID

PF3D7_0212400    837 YFCKYFYNNMDNYFKYNYMKKSTVRKKYVKNKNINDTKGHNKNNNNNNNIYGDDDDNNIY
PBANKA_0309200   726 FYSKYLYNNYDRGNKIFFGNNNFN------------------------------------

PF3D7_0212400    897 CHDDDDIYCHDDDDIYCHDDDDDDNIFIFFEKIVIFFCNILQINKCFHILIEIKLNYYFKI
PBANKA_0309200   750 -------------------------FCFFKNMIIFFCKMLKINKCFPLLIEIKLFSYFKL

PF3D7_0212400    957 PYDIKNTFINLFTYLFFFSLKVQEFSRFQIMIVRCLSFLLKKKNINKLNAYIFQLFSYLE
PBANKA_0309200   785 PYGIQNSFVSIFTYLLFLSLKYKELQCFTYEIVKTLLFLVKKININKLNTYLFQIFSYSE

PF3D7_0212400   1017 NDQINININEKGMVHRKSSKYHRNNQEEYSHNNKTNDNSVSNLYRDIENEYDENHLERRKDR
PBANKA_0309200   845 HKEMERRE---------------------MAKNMNTDVDNNIFNSNVFLNNNSEHGQNNN

PF3D7_0212400   1077 NVFSSNMNDDKKYNNLSDFKYTKENMDIKENFRIDISFLKIFFLLNDVRQINLNESNGRK
PBANKA_0309200   884 DTFGNFQNNNMNCVNFSNFKIEKESMDIKENFRIDISFLKIYFLINDVKTVRLHKN----

PF3D7_0212400   1137 DKLESKAKRRIQKLDVHRYTYNENDNNKYNDGNTFLSSQDEEKSKSFDSSDSCSVDEKES
PBANKA_0309200   940 ------------------LSNNESEQNKNISKYSHKKSYNEEYDEALNKMEKKKMKITQQ

PF3D7_0212400   1197 SKGLYGNDFVNSSDHNNNSSNNSSNNSSNNSSNNSSSGRNNSSDEVVVDPYDYNNYYECK
PBANKA_0309200   982 NKYEYN------------------------------------------------------

PF3D7_0212400   1257 DSNKFGVVVNYFYAHLPNFEKSYNINYVVEDISFDDIFLISIMDLWETNNNNNLLNLIND
PBANKA_0309200   988 ---------NFYNSNLNSYEKTVNSNHEIEEISFENVFIITLIHLCEIDNNK--INFVTD

PF3D7_0212400   1317 LLKIYEEEKKKKIYICTSLLLKIFKRIIKKKSNSYFLFNIYKAFENDIKLILDSINILIK
PBANKA_0309200   1037 LLRLYDEEKKKNIFPCTEGILKLFKKVINKKTGISFYSNIYKCFQRKIEQILDSINTLVK

PF3D7_0212400   1377 KWVVWTFKNCDNIFNREKNINIKKLVKLFFISFYKYLKNYFLQIYYHFFYNNQIYNRKNY
PBANKA_0309200   1097 KWTKWTFENCDNTFKREKNIDINKLVNLFFISFYKYLKNYFLIIN--DFLNNIKIYNSNS
```

```
PF3D7_0212400  1437 NFDNFFFSIFSKYINKIFVEIYSSSSSSTSSNSSFVFNVSKFYMMKMCISIINNMIGVVK
PBANKA_0309200 1155 TFDEFFFFIFSKYINKVFIDNFSS---------SFIFKISKFTILNTALYIINYMLKISE

PF3D7_0212400  1497 YINLERVKQVFYEHNIMMDVHMKSHLHHDIDVYYGHDNSYNNIYQKIIKSYRGEEKDTLD
PBANKA_0309200 1206 NVNTHTIKEFYTHYNNQNKLYLQNVKVEKSHIRNNNSSFLSYPYNENDQDG---------

PF3D7_0212400  1557 VINTESVHQNRNEDDIDGSINSLDVFNEIMRNIIINHNSLIKDHNDMCTKKKRINIFQIS
PBANKA_0309200 1257 -----NIHTEHTPYDLQLSKKNTITFNNFVNNIIINHTNLLSKP--ELYLHKKINIFQLH

PF3D7_0212400  1617 SPATSEQLMNNHYTMNYLTDVMLLQKDYIYNIDNNMNEHKQNVFNKPFDNNNNNNNNNNF
PBANKA_0309200 1310 MANENEYFTN-ITTMSHVTDFLCIEKESVNNKLDFSHYRNN-------IENFEKRYSNNF

PF3D7_0212400  1677 MLNYFNYIPENNNNNYRMDIKKRYPPESYDNNYYMFNNIKNEEENILLQNNSMSSSIYID
PBANKA_0309200 1362 NLEYDNQVSSVENLFQKPTINFNLNNNDFLENENTSNKNKNYDKTRFDTN----------

PF3D7_0212400  1737 KKLMKDTKEMEPLFNKTKDMKNYNEEQKNNELISYPYNNMLQNNIIFVKFFLYTQNLLQI
PBANKA_0309200 1412 -------------------------EFFENNSVNMIHKNTINYNLMFQNFFCHIINILEI

PF3D7_0212400  1797 IFQNNYIFFLSDFLFINYKKKEYIEEKKNGNQNVINIKDEDKHITNIKDGDKHITNIKDG
PBANKA_0309200 1447 IFQNNYVFFLSDFLLVHYEQSEKNKK----------------------------------

PF3D7_0212400  1857 DKNITNIKDDDKNITNMKKKNNKNYLTILMYNSQECSFYYSIFNTLINDYNFLYYSKKYD
PBANKA_0309200 1473 ---------------------------IFKYHLSNSDNYYSNMNFFINNYNINYYKNKDI

PF3D7_0212400  1917 PDLLNSFLMNTHKKNKGYSKLYKQIVNKIIYMYSLLLNIYANFYFIKEIHDIATNISNQI
PBANKA_0309200 1506 DLEN--------SKYKTYQKLNEQIINRVLLMYSLILEVCCRFKFIKKINNISTNIANQI

PF3D7_0212400  1977 FSLFDTSHFLLNLFPKNLYN----------------------------------------
PBANKA_0309200 1558 FALFDTSHPIHNLFSKVCLNSLPKVSPFYFSKFFIFFHFFFIFFSFFFIFFHFFFIFFQF
                                                    ************************

PF3D7_0212400  1997 MVEGYNVVSKSLNNDILYKKKKGEHVTRENLSLSLLSFFLEDYKSSCFLYESLNTFFKIN
PBANKA_0309200 1618 IILQNDIVSKSLNNDILSIEKKHSNEKREILSISLFYFFMHNYKNIYTLYDNLNKYFCSN

PF3D7_0212400  2057 NFNNIYFLCKYSSGYLPLERIIKLFMDVIFGHFIKFININENINDYELLEVLEYNGNKCY
PBANKA_0309200 1678 NFSSIYFMCKYRKGYFPLLSIITMFTGKIKFYYNKINQVQCKN-------MKIEYTKRIL

PF3D7_0212400  2117 ELLRFLFFFIKQNDLITINIYKYIFDIIMCILEQYIAHVN---YYIYKGKAWDVFFNKLK
PBANKA_0309200 1731 KILKFLYFFIRNNDLIDVSIYEELFDLLFFIITKYFEMGNNKEDIYKSYDIKSCEFNKLK

PF3D7_0212400  2174 ILNLSLHFVNSIYFNIFCDDINAEIKRENDNNKSNDNNNKNNHNNNNNKNNHNNNNNKNN
PBANKA_0309200 1791 IFNLSLHFMNGVYLNLFNEKYKDENSQKNSNIS---------------------------

PF3D7_0212400  2234 EKTQNVDEQRRSRLWNIVECLFYNVINKLHVNSINCLKKNKLGSYKCDEEFPKELNCKRY
PBANKA_0309200 1824 --------EKKESLWKYIELHFNNFINKLKINNYIFIKS------------------YEH

PF3D7_0212400  2294 FLNYNKDFKKEIYYYLYNLNIASEIFELIIKAIYINETKIYPLIINICYDRNISNIFFNI
PBANKA_0309200 1858 MPIYDKHVKKELYNYMYNINVISEIFDLVIKGLYTEEINIYKFIIQICYNRNMCNIFFNI

PF3D7_0212400  2354 DYDNLNSILEKYTYLHKKKKDHIKNLKYLLCKNKSIHMHKYISYIDDDHLINNMLHLLRR
PBANKA_0309200 1918 NYVNLNNLLSSYCKLYNKKIDTANNVKDILSNNKNILIEKHG-SISDDILIENIFQIFSE

PF3D7_0212400  2414 KNIYYKYVLNINEYNNFLDNHKCKRKRKFINYNNIQSSYNNNYNIYNNTNNFYEYHDYIA
PBANKA_0309200 1977 KNYYYKYILNLYEN-------------------------KAGNDQNEENCIFERKNNSLS

PF3D7_0212400  2474 IKNILHKKIELLDDDYICSRILDTQSQKTYGEKNYLFDVKNYIYNMNFINNNYQENSYIN
PBANKA_0309200 2012 IKNVLQNKMELLEDDYISKRLFEKKSKKKYGEKNFVFDIKNYCYCLNVQKNNLQNNTSVF

PF3D7_0212400  2534 DVINGKKKMFTLQISEYDKHTNYNSLFMDCVQNHHNIKKMNSTNNMNHHINTNNNYLHNH
PBANKA_0309200 2072 NLHKENKNLINGNIHCNNETN---------------------------------------

PF3D7_0212400  2594 NFISNYNSFNVHDNKKIYSYNENCKSDEIMQKKIDMSIWKNIDSIFPETFIDSDKQPAYN
PBANKA_0309200 2093 -------------------------PDIKLKNKIYKSILKNMDAIFDNKNTNNEKK----

PF3D7_0212400  2654 FDPIDSINLGSSRSNNEKKKKYIQIDNPVKKECLLLNINYDKHDSIVYNKYDNMFHYDEL
PBANKA_0309200 2124 ----------------------------------------------------------KI

PF3D7_0212400  2714 PDINNNNNNNNNDNNNNTCVIEDIKDIYEKRMNKNTKRNKEKKEKRKYIFLNNFNNNKEK
PBANKA_0309200 2126 KNTSLKFDENNNEDNKMNNWNDKIDRIQNIKNNKFEKIDRKKKDKKKTIYYEYSDNISKS

PF3D7_0212400  2774 KMKNNQKTVYSNNNIMGEEFYNEFYLHNFKNEIKCMKYINLTQSLYDVKYRLLLLFYKFI
PBANKA_0309200 2186 FMFS----------------------KYFKNEIKCMKYINITQSVYDSKIRLLLFLYKFI

PF3D7_0212400  2834 IILKHKELLQNENYIKEEKEFLKKHHIKKNIPFLFFIYELMITFFNTAENINKNTYYYVL
PBANKA_0309200 2224 IILKYKKLFEIENYKKEEEFYLKKSNTKLNVPFVFFIYEAMCTFLVPSIQINKNSFYYVL
                                                ***********************   ******

PF3D7_0212400  2894 IINILVNLFLFINKRDYDDETCMSNIINNDNNKKNKNNLIENKNEIYNTNIKSLKNDKEY
PBANKA_0309200 2284 ISNIIVNLFFIINMHSYEQKKKKNSLENENQTKIPFIINTNIENTNYVNNKQNEINNIDN
                    *****************

PF3D7_0212400  2954 IDNHSN---YAMFYCDLFCDDFFISNGKKNKENVVFHTLHNMSHKEMSKYDLIGKNKYLE
PBANKA_0309200 2344 NDSYFDDEYENSYYCDLFLYESYSTNIKNNNNNIFYVLHR--------------------

PF3D7_0212400  3011 NYINNLILEKKKKINNLNVHINKKMDNNILYSFINRINETRDNTKKKNKLYIRRYYLKKS
```

```
PBANKA_0309200 2384 ----------------GSSFISEIYRKQMNWQLQNEGYNDIDNKNSKIENKDKKKPEKNS

PF3D7_0212400  3071 IKYNNHLYNMPIFLSLFLRCVTIHLHYFKFYNSYIYFLKHYNMLHIPHAVLKHLYSTHHF
PBANKA_0309200 2428 IEDSKMICNMSVFFSLFIRCIIIHLHYFKLYNKNYYFLKNLNALYVPNCILKHIYPAQNF
                         ***********************

PF3D7_0212400  3131 NINLFVNMLELFYVFIQIYNNYFVSFCDISSCRNKHVQRDQRCLNNNKNKSEDNEKIYCT
PBANKA_0309200 2488 NINLFISLLEVFYVSIRLYNNHFVKINKGR----------QFDEDFKFDEVDKKNKNSKQ

PF3D7_0212400  3191 NNNGDGYDDDGYGEKNVSGIYKENNNKINVKGDIYNIDNINVYPLNGKLVSIYLNTLKEI
PBANKA_0309200 2538 QNIFDNEKNKEINKKVINYVKNKQLDIYNSGDNIFNFN-------------IYIRTLKEI

PF3D7_0212400  3251 LKECYECHLGHMKNNENMEKSFFIEHLLLYFLYNRINTIYELFYNFYFTYLRKKENNNDI
PBANKA_0309200 2585 LTECYESHDMYMQNEENNKMSYFMEHILLYFLYNRINTIYELLYSFYFKCIKVEKTK---

PF3D7_0212400  3311 LLDIVNEHIYNLIGNKIYDQINKINNFLDDKQYYYFYINTLTFITLNKQICIYIIKKKIL
PBANKA_0309200 2642 IIDIVNKQIYKNLENILYDHVNKINTFLYNNQYYYFYINTLILLTLNRQICLHIIKTDIL

PF3D7_0212400  3371 NKLIYIPFIYHSLFDKNKNFTSIYHINNNQYIRNKDHIIFCSLIVFIIKLLYVFVKNFKH
PBANKA_0309200 2702 TKFIYVPIIYHSMFDNNKNFNSIYYISNGEYIRNKNHITFCSLIVLIIKMLYIYANDYKN

PF3D7_0212400  3431 KNVNFNNHQNNKNHENVNTNVGHVFHSSFISENPYYHVTKHLDYYNDHTFMESMKVATNR
PBANKA_0309200 2762 FEENAEFYFSEN------------------------------------------------

PF3D7_0212400  3491 IYENPYYRNYNKEPYTSNEHKILNSNMDINNSNHFLRSNDENVKTNTNTNTNTNTNTNTN
PBANKA_0309200 2774 ------------------------------------------------------------

PF3D7_0212400  3551 KNSDTDNHNDAYNHNDAYNHNDAYNHNDAYNHNDAYNHNDAYNHNDAYNHNDAYNHNDAY
PBANKA_0309200 2774 ------------------------------------------------------------

PF3D7_0212400  3611 NHNDADNHNDTDNHSDNYNSHKYKGTYKIYRIHDEEDIIQDNNYTNDDYVNTNYNFHQNN
PBANKA_0309200 2774 ------------------KSYNFSKKLKKINEWNSEGIEIDEMHTNSFFFENISSINLQI

PF3D7_0212400  3671 NYQNSSKDNSHTYTSSGDDNLNNPFISLNKENISKKKKNKKTNKNREEEKKKKNEIIHNE
PBANKA_0309200 2816 NPKEMITGIQNDVTNQRNDYISNNEINIQNKNAKHFENDNKNAKYKGKENLDGRCYTNKE

PF3D7_0212400  3731 VEYFLEAILNVVGKLSDRINFIFLKIEKINCLAIFEESYLYVELLTKISYYCNIYNINNF
PBANKA_0309200 2876 VVFFLSSIFNIIGKMSDRINFIFTKIEKINCLAALEEAYLYVELLTKISYYCSLYNINDF

PF3D7_0212400  3791 LISLMDKRVEHHLFYINRILEKFIYEKEVIIYPYSPYEINASTNVPNNQDKKKKKKSTKN
PBANKA_0309200 2936 LISIMDKAVDHHLFYINRILEKFIFEKEMIIDPYSQYEVSASS---NSQDYINQNKLKKN

PF3D7_0212400  3851 ELTLFTQRVLYICYKSILNYNTILLNLIQTPYFKNSHFVIHLYILILKSTRLVTNIIEHF
PBANKA_0309200 2993 GLSLFTQRILYICYKSILNYNTILLNIINNQYFYYSYFVIHFYILILKSTRLVTTIIEHF

PF3D7_0212400  3911 GKKNTTLIRTIKANDNNSYVPICLDILNTEKEKKKEKIHYTRGVG--KSNFTYYLSDSNT
PBANKA_0309200 3053 GRRNTTLIRTVKANNGISNVPICLDIINTTKEKKKEKIYYTRGVGGKSSSFCQYLSDNNS

PF3D7_0212400  3969 RSEESAYPGEIIQYNKSIDEYINTKRVYKNDHLFNFLPEIISLKTYYNILKEILERSAFL
PBANKA_0309200 3113 KSDISNNSGEIIQYNKSIDEYTNTKRIYKNDNLFNFLPEVIQLKKYYNILKEILERSAFL

PF3D7_0212400  4029 GAFVLDKLKNSDEDSCLKQILVSNIFSYLIHINATLLPSNICTNNLKKKCTLIYNYVMNV
PBANKA_0309200 3173 GAFLLEKLKILCEDSCLKQILISNICSYLIHINATLLPANICTNNFKKKCTLIYNYIVNI

PF3D7_0212400  4089 HKK
PBANKA_0309200 3233 HKK
```

E

Plasmodium berghei Nup434

P. falciparum syntenic ortholog PF3D7_0212500 conserved Plasmodium protein, unknown function

```
PF3D7_0212500      1 ------------------------------------------------------------
PBANKA_0309400     1 MDGNDFRFDAESHVNNESENDDINSKKKNEESDRDISNGSQSDRSQSNRSQSNRSQGNRS

PF3D7_0212500      1 ----------MEYLESEKSSSDDRREVNNFENDYSKDSSHSNINSDLDVDRKKHSDNVYE
PBANKA_0309400    61 QGNRSQGNRSQSNRSQSDRSQSDRSQSDRSQSDRSQSNRSQGNRSQGNRSQGNRSQNNRS

PF3D7_0212500     51 ESEQDGKQTEGRKKIKGFFKLKKGDSEDENKEKETKDHRLKDGGDTFEENINVLKK----
PBANKA_0309400   121 QSNRSQSNRSQSNRSQGNRSQSDRSQSDRSQSDRSQSNRNISGKQVKSDIECTEKENISE

PF3D7_0212500    107 -----------KKKKKNSDTINYNKKYFNKNKHNGSSSNEHSSYSDENFFEAAKRNKILN
PBANKA_0309400   181 VDEISEISKEHERKQKKTRKKIALKKYRKYFTKNKSEEKEIKSSSDENFFTAEKKNKRKN

PF3D7_0212500    156 EEIYKNKDNEDMMCDMSIFNDDNNMDDSLFNKNEDNNRYDEEEMKKYKRKGKRYSSDSYK
PBANKA_0309400   241 EERYTINNNVENNCDMSIFNDQP-INDSLFSQPQNGSETE-----------KGSEKGSGD

PF3D7_0212500    216 DDSPQYMSERYSSEKYSSEKYSSEKYSSEKYSSSNRNQSTNLLNNIKNFCNTYIINKKKD
PBANKA_0309400   289 NVDESIYEEEQTSEYKKSTETYSRKYTDEDVSRSMESNHSYDNNRGSQYSQSYKSDQENE

PF3D7_0212500    276 RSRDTYEDEESREGAYGENTTEDLNEDTQEGHKNKKKEILMNILYNDINIKKNDEKDFFM
PBANKA_0309400   349 SICDEMESKSSYKNVKKNYKIHEIRNKIKN--VNKKSEILLNMLFNANEISMNKSGTLRD

PF3D7_0212500    336 DRNFKGKKKEIDIKKNQQVMKNMLNIKNNENIDVYNDKDNFINIDDKCPSG------YFK
PBANKA_0309400   407 NIKNKQENKDKQFENNQKIIKTILNVQEKNKVDVYYNENNFTQIYKEAKGEKGGPIDTHK

PF3D7_0212500    390 DKIKEYDYLDNEKQKNVNKMIHPKDGNNNNNNNILLSQNSSTILSHVVQEDYADGIKKFN
PBANKA_0309400   467 INKQHKTEKKNEADSIKQNVIEKQIYELRDNEKYCFIKDKNDLFVGNDNEEFTEIKKNYN

PF3D7_0212500    450 KNSFYNNLENRKLININNIYDKYKIILSEIKSGDHLNNVEKKLNVIENSLLCIFDLQDGD
PBANKA_0309400   527 KIYEATKLEKRKIMNLNYIYNKYKILLSEIKSNEYLNSIEMKLNLIENSLFCISDLYKEG

PF3D7_0212500    510 NNNNINDDDDNDDDGHHDDGHHDHVGNNMKGDKLDIKKRFSELFDDALNTLIENYEDVKK
PBANKA_0309400   587 NDGVVDSKLD--------------ITQKLSELFENVTENVLDTYDEAIKGKDVDNLNINK

PF3D7_0212500    570 KEHSENINKKAQLNLSCDFNLYLRICKVVLNDEKHIHTSNKKNMLYYNNIYKINYIKSNN
PBANKA_0309400   633 KNNNTEKKKNEQLNLSCDFNLFLRVCKIIINDENVVYANSKKNILFYNNIYKINYLFNCV

PF3D7_0212500    630 KNKDILYNIHCRNIHLYLILSSHEGIERKKQQMHILKDKDKDKNKNKDKNKNKENILNEN
PBANKA_0309400   693 KNNDILFNVYCKELIHPFEKLFKG------------------------------------

PF3D7_0212500    690 IRKIQKQNKKQNQNFIDIDNIYYTNKLRNINNVNDMNDMYHIHNDENDVINQKLYYDEID
PBANKA_0309400   717 -----------------------------------YKEIKHEHVNRSLSDTNQIKWPNFS

PF3D7_0212500    750 YTKKGILDKIHVLWFYEKKNEVHYLHLYNLNNINSKMLSIIIDGYLECIDVVYLNNFFPL
PBANKA_0309400   742 YTKKGIIDKINILWFYETFDEIHYLHLYNLNNINSKMLTISIDSPVECIDIVFLNSLIPL

PF3D7_0212500    810 ESFYNSVKNYYLMLITENSFYFFNIHLYFNCEEDYNISENEKLIYQNIYPFYFNIYLFLL
PBANKA_0309400   802 DSFYNSIKNYYLMLITEQTFYFFNIHLYFNCEEDYNISLGERLIYQNIYPFYFNIYLFLL

PF3D7_0212500    870 FKVPKDSKMKFEYIRSHEKSERIFLVVNDGNVYEFIYEKKNLFEPFNIFKNIIVNSISSL
PBANKA_0309400   862 FKLPKGSKKKYEYIKSIEKSERIFLVQDDGNVLEFVYEKEDILKPLNIFKHIIINTLSIL

PF3D7_0212500    930 INVVNEKIFHHSRNYMIEHTANDDNSSFNYYDNGKSSINSYNISNTYP------------
PBANKA_0309400   922 VNKVNNAFFSNNRNIEYYSNRDEEDDELIKSDRSQYKQNFNNFHKLRSSPKISNQSVISG

PF3D7_0212500    978 ---------------------------EYMDNKSFIDEYNKNIYCDDLHDYPIKFYLKKI
PBANKA_0309400   982 DEYDYANNSKVSLIDESDKNYSENKTQKRKEDDQYLYQYNKNIYCDNIQQLPIKFYLKKI

PF3D7_0212500   1011 ISTYFIKYFAFFKNKVKKIIIDNERSILYVLYENSDLYVKLLSNRADVNNKKKNYLSDTI
PBANKA_0309400  1042 INPYFIPYFYFHKNRVKKILIDSERSILYVLYKNSDLYIKLLSN---PSNKNKNYTSETI

PF3D7_0212500   1071 IFTKGELIKELNNIYFIDDMNILYNDMYNMNNINNMVNMNNINNMVNMNN----------
PBANKA_0309400  1099 IFSKNDLVREVGNIFFVDDTKASHHDIYMGSNSNNNMKISNDQNFYPLNVNIDGIGNSDN

PF3D7_0212500   1121 -MNNMNNMVNMHNMVNMHNMVNMHNMHNMHNMHNIPNMHNMNSNTNNKFG------FAHN
PBANKA_0309400  1159 YIDSNNVAHFGEKHFSNSNNFASYGNRNIHNRQGINNDITGNSNMKNRNNNNINKFMYPS

PF3D7_0212500   1174 NIDPTTFHKLNIIDIHINHIHERNNIFLKMVDNNFNIYYLSLVKNMDTNNYKLILKDFQN
PBANKA_0309400  1219 SMDQGLLNKLEIIDIHINYVYERNNIFLKMIDNNLNIYYISLVKNIENNSFKVVLKDFQN

PF3D7_0212500   1234 YPHKKGLKINDKNSDVIFTHHLKNLYIILKKKNLKKKEPNKAVVHKDDAQAKKKTFFFFN
PBANKA_0309400  1279 YPHKKGLKINEKECQVVFTHQLKNLFIVLKKVTKGKKK---------NTDEKEGSSENYG
```

```
PF3D7_0212500  1294 KDKMDSQKIRKTHAHKRRSTILRRDTSRRVDKNTQGKATYKMMSKQKRGKIRKRGEEVGE
PBANKA_0309400 1330 NDEWNATKKHDGVKTKGRDMFDKSNNFGFSQPPPFKFMGDNIMNSQEINYKNSENEKKKK

PF3D7_0212500  1354 EEEEEDEEEEDEEDEYEEEDEYEQEDEYDEEDEYDEEDEYDEEEEEEEGRRRKKSYRIKK
PBANKA_0309400 1390 NVNKKIHKEGNTDYEIDWEDAHGYFDPEDENKDQDKNDEINKRASSDNKQNK--------

PF3D7_0212500  1414 HSSNKNLNDDEEKNTYQEEDQDSYDDISDNANDYNNNYNNNYNNNYNNNYNNNYDNNYDN
PBANKA_0309400 1442 ------------------------------------------------------------

PF3D7_0212500  1474 FNFEQDNESEEEKVCYKLKLLTCADDIG---NTNQMSSSNKNKFIFKQINEYYINEEIIG
PBANKA_0309400 1442 ---------ETEEVYYKLKLLTSSDENGNFLNSKNSSNSPKSKFIFKNVNEYIINEEIIG

PF3D7_0212500  1531 VIYKKKNYYFHEFFEHTENDNILKDFYVNKMKQDNISNIMARNEKNRFHLNSQVRSEPNL
PBANKA_0309400 1493 IMYKKKNYYFHEFFETAQKNNAIKDLYLDKMKYNNIN---------------EIPSVGRI

PF3D7_0212500  1591 RTTLGNNLTNTNMMPNNMSTMSIPNMNVNHMNVNHMNVNNMNINNMNINNMNVPNRNMPN
PBANKA_0309400 1538 GNIPPINEPNPNLGMSHHNSIPNNLINNYTKSSSSGTTNNMNFLKNERNGISMHYYDNSK

PF3D7_0212500  1651 RNIPNMMHVDSQNNLYNSGYNSKPSGNLYDERNFMNMGEVRDKMESIPPFYLYENTVSEY
PBANKA_0309400 1598 KNLP--YYLNSQNNFMPNKSNLIINDNLLFKPNDRSSSDHKDNLKNIPPFYLNENTVSEY

PF3D7_0212500  1711 QEYYDLMIITKKHIYYISKNTRTQKIEKMINNYLPYKISYENRNIESVVLKEIKIKEYGK
PBANKA_0309400 1656 QEYYDVLIITNKSIYYISRNTKVQKIEKMINNYLPYIISFENRNIKNIFINELKIKEAMI

PF3D7_0212500  1771 KNQED-----------FYKSIKQNMLMKINNDKNNIISKQLINSVSGGIPIDIPPSPTSL
PBANKA_0309400 1716 TNESTNLEGNHFPTQILEKQVLEYVKERSNNGNIPFNKNLDGNNMGKGENDSTSNSMAVF

PF3D7_0212500  1820 IKEKNEYEEIYEYFINQIIEISSTEEFLFIIWSILLNHVYKYEIMCFSNIQSSNQRDIKN
PBANKA_0309400 1776 LKKKNEYELIYEYFINQIIQMSTSEEFLFIIWCLLLNHVHKYEIMCFNNIHNVADSRNSN

PF3D7_0212500  1880 KLNDDKSKRNMDAIHN-MDNTNYYNSYGIPRMSMNIAGSSTGQDRKKLFLQNFKKNDMNN
PBANKA_0309400 1836 ISGKHSDYRSNSKIENSQTAYDLGFSKGYDNNFKNNFNYSIQGKNQHNRIFEFNHDFNIT

PF3D7_0212500  1939 NNNNNNNDDDELLSQNEDEQNMFENSYIKKDWNNYNNNNNNVDYDVLNKIYKRTPFKFGF
PBANKA_0309400 1896 DKSLTHKHPFNRIDMNSINYSNNNNNSFKLKEGNHDIDNGHIDYDILNRIYKRTPFKFGF

PF3D7_0212500  1999 VDKSADYIIKQGKLQKAMELEIKTRKVKGINHLFNDNIIDDLENELTKSG--GNNKNGDY
PBANKA_0309400 1956 IDNNADYIIKQGKLQKAMELEIKTRKVKGINHLFNDNIN-ELDNEIGKNGNKGNINSIES

PF3D7_0212500  2057 KNKNDNVLDNENILLNDAAYSNLYNAYQKNSSKQKLLEAERNKSGTFAFEYLTNKNFDIV
PBANKA_0309400 2015 KYQGTNDSINKKIVYSDAGYTNLYNMYQKKNTRNKLLTSEKNKSGNFAFEYLTNKNFDIL

PF3D7_0212500  2117 FLKNLKCYNNSLHMLSRGLLLLLSRLLKPVMFINLFSYEKYRDVNVFKCSSN--------
PBANKA_0309400 2075 FLKNVNMYNSSLHIISRGLLLLLSRLLKPIMFINIFTYEKYRDTQFFKFLNYSNEKKESK

PF3D7_0212500  2169 -----------------VPYKSRKYMKVSEASRGVGISIISKEKIEEDEEESFYNMKDFC
PBANKA_0309400 2135 YNDKNYTEQNNKYRSYTKGIKFQKIGEKHSKNYYKNSISIICNDKDDEIEESFYSMNDYC

PF3D7_0212500  2212 IIRMSDYMYS-----RRKRKRNILEDDSNMLILTGNLPLDFCLNLVEKLKCLSLCVSIVL
PBANKA_0309400 2195 IVRINDDYIVNFTKKGNKKRKRNIYESNDILILKGVIPLDCCLNLIEKLKCLSICVSIVL

PF3D7_0212500  2267 RSLIKKKNEYTTKIKNIKDVSIINLYYDYTLIMINEINEFYSILNFINFSIEYMLIYCII
PBANKA_0309400 2255 TNLLKRKKDYANKMRGITDVSIINIYYDYTVIMINEINEFYSVLNFINFSIEYLLIYSII

PF3D7_0212500  2327 ICDYYKNKSIIKKKNIGNKKN-FIYLSSTLFDLLIKCNFMRTYLSSTYRNILKQCLFNII
PBANKA_0309400 2315 ICDYYRCKKIIKKKNVDKKKNNFLYLPSSLFDLLIKCNFLKVYMSKTYREILKQCLFYLI

PF3D7_0212500  2386 RSGKYIPIEFFQGYIIHKNEFLALFLTKRIEEEVFLKKKMKVKEYYDYNNNKSKTDNNLL
PBANKA_0309400 2375 KSGKYIPVDFFQGYIIHKTEFLALFLIKKIEEEVFLKKKMKIKDNFEYYKNDGRYDT---

PF3D7_0212500  2446 THHHKYGDFMASSEKRAEHFINENLKNAMISINIYKLIILLYKADYYRCTSTLICLFIDS
PBANKA_0309400 2432 ----KYDEFLAS-EKRAEHFINANLKDSLVNINIFKLVILLYKADYYRCASTLISFYIDS

PF3D7_0212500  2506 FYKTLGGDKHKNLILKDEVITDTKQGKHDMNDYNMNKKNNMDINITINNNNNNNNKIYN
PBANKA_0309400 2487 YYKTLP------------------------TYEKDVNKDNQNGNIRDSRHGHYIDKNLNA

PF3D7_0212500  2566 DNTLNVYNNSYNIHSNHLMNDKRKNAQVLEKHLKMLCDNFFNLEEFYSSNIIINNMDIEY
PBANKA_0309400 2523 YNYENMLFSNDNGHIDNKMGG------------------------IYNNEMIISNIGIKY

PF3D7_0212500  2626 TYDYFILYEKCFLPIERIVHVNYMKYLYKNNERKKNKIRKFLITLLEYSRDINFHFFIFN
PBANKA_0309400 2559 NKYFYDLYTSCFLPVDKIVHVNYLKYLFKHDERKKNKIKRFLITLLEYSNDINFHFFIFN

PF3D7_0212500  2686 LILYKCKNEFPCSIFELHISQYLYFFVKLNELNIKDAYIYYFNNFKYQDMIIYFSRKAFY
PBANKA_0309400 2619 LILYKCKNEFPCSIFDFTISPYLYFYVKFNEMNIKDAYIYYFKELKYQDIVIFFSRKAFR

PF3D7_0212500  2746 PWETNVEQQKKQTLSYIYNDKIKKNKKNNSYYEMNNNTYMNEHGYTDIENERLNKKNKRL
PBANKA_0309400 2679 KWDISPDHITSLKSISKILNLKKDNPINKNEGSKGP------------------------

PF3D7_0212500  2806 NVRGRTNTLDDIIVSDHGNSYDKYNTSKHNRRKNHINEMKKKQNNKKKNTLFVDGKDMEG
PBANKA_0309400 2715 ------------------------------------------------------------

PF3D7_0212500  2866 IGKEKEKENKNMNNNIFYNNSYSNINNSSYSNINNDIYSVDNMTSVNNTKYVSGVPSYAH
```

```
PBANKA_0309400 2715 ------------------HNSVRKIENFEKPKIDNQYITKEKQFDIEHDKMNKISNDKFL

PF3D7_0212500  2926 VLINKQVNEYYQGLPNYNNMMIKGSHIINELPKNNYIYENNYIGQNYLMTNPLYNKETKD
PBANKA_0309400 2757 DPFHFSVKMANQHKNYEQEINIKNMLLNELARNNYLFENNFIDNNNLTKNPLYNNETKE

PF3D7_0212500  2986 IFYTIYKYLFKIISYPSLKKRMEFIDNCMKTKIFVIRKVCNFKNRPFSSNKKNNKMNRDS
PBANKA_0309400 2817 IFCSIYKYLFRIISYPSLKNRLEYIDNCMKAKIFFIQKIFYFKKK---------------

PF3D7_0212500  3046 SYVDNISSYYDDDNNNNNNNINILKKKKKKREVGLGGIRLRNGVDNKRTHDDTIDEKYKN
PBANKA_0309400 2862 --------------------------------------------QNKNEPQNSIPINYIN

PF3D7_0212500  3106 NRNYLFMNGVDVLYNKDQLGYYKNSLDDNNNNNNNYNNDNIRRSHVSSCSYRRAHNNIKY
PBANKA_0309400 2878 GEIYESNNEVKSKNTDYNRLEFVRRNGNKKNNNEYFINDEVVKVPRN-------------

PF3D7_0212500  3166 DIKEGGSDNIYTSNIKRNKKKIKNIEEIFNINSMLNKEALKNYYTVDKTILYDESFSKLL
PBANKA_0309400 2925 -------------------GKYSSVQDILNINCYLKKEVLKNYYLVDKTISYDNIYSNSV

PF3D7_0212500  3226 KGIFEKNKCLFKLKENYWSKQNSYYLHLKDIKKCRTCLNYQRLLFHEVINLFVFYVYKFC
PBANKA_0309400 2966 KLLFANNKCLFKLKDEYISNHNNYYLNLKDIKKFRTSLNYQKMLFYEIVNLFVFYVFKFC

PF3D7_0212500  3286 NWDVLKNYFDILINGSEEAIIKVLEHFRNINKEQIDVIRKSYNNMYEYLSKSKYEHIDDI
PBANKA_0309400 3026 DWNFLKNYFDILMNGSKEDVKKVLEHFNNINNEEIDVINKSFSNMYDYLCRNRYEYVDDI

PF3D7_0212500  3346 INDYNNKINNMERKININRIIDIIDIFKEYLLLIQQEIHTKEGLKNHIYGKSKILFKNFL
PBANKA_0309400 3086 INDYNKKIKNVNSKINISRIIDIIDTFKEYLIVIQTNIYTKEELKNNIFFKSNFLFKNFL

PF3D7_0212500  3406 PSFNLLKLIILCDKKKEKIENLNTNCFSTVNNMLRNDMIKGSTFYFSKYSYCFERLLDYA
PBANKA_0309400 3146 PSFNLLRLIILCDKKKEKIDNLNNNCFSTVSAMLKRDPKCN-SFYFSKYSFCFERLIDLS

PF3D7_0212500  3466 FSLTITFENINFIINYIGDVLKLYEVDFKNSLYLLVIIKLHKFINNLFEITKVREILKTK
PBANKA_0309400 3205 LSLAICFENVHFIINYIGDILKLYDKNFNISLYLLVVLKLHKFMNNLFEIKKIRLLLKKK

PF3D7_0212500  3526 VSILKNNKYYERIKLLYLLNCIPLIYLDPHMNVILINESYEYKMNDDDKIIFSKLSPFSL
PBANKA_0309400 3265 IDYIKSQKYNEQMKLIYFLNCIPLIYLDPQMNVILINESYEYKGNQD-QIIFSKLSPFSL

PF3D7_0212500  3586 VSKVVNHKLRSVYTYHDYTDNLENEEPIHKNKTSKSMNDDTKSVSHYEETKKKNDDDDMS
PBANKA_0309400 3324 ISKMVNNQLRSVYSYHDYTDNYNDDE-------EEGIKNDSKKSNQTKQDKKNERNSDIS

PF3D7_0212500  3646 YDSSSDYPKDISYDTSDGSYRDNNNNGSGPNDVKQMKEKGIPKVSKENAKNKKKNVNVNI
PBANKA_0309400 3377 DVEN-----------------------------------------------------IQS

PF3D7_0212500  3706 NINNKNDESYNIHNKIKKDNIIAIDKDDRKTLYYLYNVNYCFNDQNNNNNNNNNNMNNSN
PBANKA_0309400 3384 NSDNESNDVGVLKRKIKYGDTTSINKEGRKALYYLYNVNYCFNKKENGLKRKEN------

PF3D7_0212500  3766 IFGNPHNPELVVLSYKNYCFYIVWISNLLLNHKIEYESLIYVYLKIYNNTKHQKASLLEE
PBANKA_0309400 3438 ------YPEVIVLSHQNYCFYITWISNLLLNHKVEYKSLINVYLKIHNNLKHKKASNLEE

PF3D7_0212500  3826 HEITIILYYLFTMWINGDKNNSSFFFYNEEKSYHEKNNLGYFLKDTYGNLQYINNYSLIT
PBANKA_0309400 3492 HEISIIIYYLFTMWINGDKNNTSFFFYNDDSSYNEKNNIGYFLKDKYGKLEYINNYSLIT

PF3D7_0212500  3886 LLKELLSRAEFYFEYTSDAASRNYDISCIILDSSIYDNEKVKKSSVDILKKFFDNIYITF
PBANKA_0309400 3552 LLKELLSRAEFYFEYTSDAASRNYDISCIILDSSIYDHENFKKSSVDILAKFFDEIYVTF

PF3D7_0212500  3946 NDVLIKIPFLNQILEFQKIHEFLKRFKIYLDEIICRITLTEGNYI
PBANKA_0309400 3612 NDSLTKIPFLNHIIEFQNIHEFLKKFKIYLEEIAYKVALSEQAYI
```
